# Genetically Determined Lifestyle and Cardiometabolic Risk Factors Mediate the Association of Genetically Predicted Age at Menarche With Genetic Predisposition to Myocardial Infarction: A Two-Step, Two-Sample Mendelian Randomization Study

**DOI:** 10.3389/fcvm.2022.821068

**Published:** 2022-04-25

**Authors:** Jilin Zheng, Ken Chen, Tao Huang, Chunli Shao, Ping Li, Jingjia Wang, Wenyao Wang, Kuo Zhang, Xiangbin Meng, Jun Gao, Xuliang Wang, Yupeng Liu, Jingjing Song, Eran Dong, Yi-Da Tang

**Affiliations:** ^1^State Key Laboratory of Cardiovascular Disease, Department of Cardiology, National Center for Cardiovascular Diseases, Fuwai Hospital, Peking Union Medical College, Chinese Academy of Medical Sciences, Beijing, China; ^2^Graduate School of Peking Union Medical College, Peking Union Medical College, Chinese Academy of Medical Sciences, Beijing, China; ^3^Key Laboratory of Molecular Cardiovascular Sciences, Department of Epidemiology and Biostatistics, Center for Intelligent Public Health, Academy for Artificial Intelligence, School of Public Health, Ministry of Education, Peking University, Beijing, China; ^4^Key Laboratory of Molecular Cardiovascular Sciences, Department of Cardiology, Institute of Vascular Medicine, Ministry of Education, Peking University Third Hospital, Beijing, China; ^5^NHC Key Laboratory of Cardiovascular Molecular Biology and Regulatory Peptides, Beijing Key Laboratory of Cardiovascular Receptors Research, Department of Cardiology, Institute of Vascular Medicine, Peking University Third Hospital, Beijing, China; ^6^Key Laboratory of Molecular Cardiovascular Sciences, Institute of Cardiovascular Sciences, Ministry of Education, Peking University, Beijing, China

**Keywords:** age at menarche, cardiometabolic risk factors, myocardial infarction, Mendelian randomization, lifestyle, mediation analysis

## Abstract

**Background:**

Observational studies have shown an association between early age at menarche (AAM) and myocardial infarction (MI) with recorded cases. In this Mendelian randomization (MR) study, we used large amounts of summary data from genome-wide association studies (GWASs) to further estimate the association of genetically predicted AAM with genetically predicated risk of MI and investigate to what extent this association is mediated by genetically determined lifestyles, cardiometabolic factors, and estrogen exposure.

**Methods:**

A two-step, two-sample MR study was performed by mediation analysis. Genetic variants identified by GWAS meta-analysis of reproductive genetics consortium (*n* = 182,416) were selected for genetically predicted AAM. Genetic variants identified by the Coronary ARtery DIsease Genome-wide Replication and Meta-analysis plus The Coronary Artery Disease Genetics Consortium (*n* = 184,305) were selected for genetically predicted risk of MI. Genetic variants from other international GWAS summary data were selected for genetically determined mediators.

**Results:**

This MR study showed that increase in genetically predicted AAM was associated with lower risk of genetically predicted MI (odds ratio 0.91, 95% confidence interval 0.84–0.98). Inverse variance weighted (IVW) MR analysis also showed that decrease in genetically predicted AAM was associated with higher genetically predicted alcohol intake frequency, current smoking behavior, higher waist-to-hip ratio, and higher levels of systolic blood pressure (SBP), fasting blood glucose, hemoglobin A1c (HbA1c), and triglycerides (TGs). Furthermore, increase in genetically predicted AAM was associated with genetically predicted longer sleep duration, higher levels of high-density lipoproteins, and older age at which hormone replacement therapy was started. The most essential mediators identified were genetically predicted current smoking behavior and levels of HbA1c, SBP, and TGs, which were estimated to genetically mediate 13.9, 12.2, 10.5, and 9.2%, respectively, with a combined mediation proportion of 37.5% in the association of genetically predicted AAM with genetically predicted increased risk of MI in an MR framework.

**Conclusion:**

Our MR analysis showed that increase in genetically predicted AAM was associated with lower genetically predicted risk of MI, which was substantially mediated by genetically determined current smoking behavior and levels of HbA1c, SBP, and TGs. Intervening on the above mediators may reduce the risk of MI.

## Introduction

Cardiovascular disease (CVD), with myocardial infarction (MI) as one of the most acute and severe manifestations, continues to be the dominant cause of deaths worldwide and accounts for over 17 million deaths annually (1). Notably, women were reported to suffer a greater burden of ischemic symptoms caused by MI, with higher rates of functional disability and more adverse outcomes compared to men (2). However, conventional precautions against CVD demonstrate a persistent gap in effective recognition of gender-specific risk factors and understanding how these factors result in poorer outcomes for women (3). Thus, identifying gender-specific risk factors for adverse CVD events, especially MI, during women’s lifetime could yield more timely prevention and improved clinical prognosis.

Menarche signifies the puberty and start of reproductive capacity, and is followed by the onset of cyclic ovarian function and increased secretion and exposure to endogenous estradiol. As a reproductive physiologic landmark of the female body, menarche is dependent on a tightly orchestrated process of neurohormonal alterations through the hypothalamus-pituitary-ovarian axis (4). Previous large-population cohort studies have reported that early age at menarche (AAM) was associated with increased risks of coronary heart disease (CHD) in both adolescent and adult women (5–11). Glucose intolerance, blood pressure, and serum lipids were reported to be increased in adolescent girls with early AAM, which might play an essential role in the development of ischemic heart disease (IHD) (12). Inconsistent results were obtained concerning the roles of smoking and estrogen exposure in mediating the association of AAM with IHD (6, 8, 9, 13, 14). As previous approaches for testing potential mediators in observational studies led to biased results by calculating the reduction in strength of the multivariable association between exposure variable and disease with adjustment for confounding factors (15, 16), it remains unclear whether the correlation of AAM with CHD is modified by diverse potential confounding factors, including lifestyles along with cardiometabolic factors, and the extent of their potential impacts.

Mendelian randomization (MR) has emerged as a new investigative approach that utilizes genetic variants as instrumental variables to investigate the association of an exposure of interest with an outcome. In brief, MR refers to a random combination of alleles in which DNA is transferred from parent to offspring as gametes formed during meiosis, a process known as Mendel’s second law. This means that the inheritance of any particular genetic variant in an individual’s DNA should be independent of other traits. Therefore, when individuals in a population are grouped by a specific genotype associated with biomarker differences, they should be similar in all aspects except for one group with genetically higher or lower biomarker levels. For this reason, MR has been described as “nature’s randomized trial.” MR is less prone to confounder bias, measurement error, and reverse causality; thus, it can provide more efficient and robust results that closely resemble those from randomized controlled studies (17–20). Based on the well-established MR framework, the two-step approach has higher sensitivity in evaluating potential mediators and is less likely to cause inherent bias compared with the traditional multivariable approach (21).

In this study, we aimed to estimate the association of genetically predicted AAM with genetic susceptibility of MI, and to further estimate whether and to what extent the association was mediated by lifestyles, anthropometric characteristics, cardiometabolic risk factors, and age at which hormone replacement therapy (HRT) was started.

## Materials and Methods

### Overall Study Design

Large two-step, two-sample MR analyses involving over 647,920 participants selected from publicly available, summary-level genetic datasets were performed using a two-step strategy to investigate the association of genetically predicted AAM with genetically predicted risk of MI and whether this association could be mediated by lifestyles, conventional cardiometabolic risk factors, and age at which hormone replacement therapy was started. Two-sample MR analyses refer to the utilization of distinct data sets to ascertain the associations of same genetic variants with the exposure (e.g., AAM) and outcome (e.g., MI) of interest (22). The association of genetically predicted AAM with genetic disposition to MI was tested first, and the two-step approach was applied in mediation analyses. The two-step approach investigated the association of genetically predicted AAM with each genetically determined mediator in the first step. In the second step, the approach investigated the association of these genetically determined mediators with genetically predicted MI risk after adjusting for AAM. The proportion of mediation was also estimated for each mediator following the second step.

### Data Sources

#### Selection of Genetic Instruments for Genetically Predicted Age at Menarche

Genetic association estimates for AAM were obtained from genome-wide association study (GWAS) meta-analysis derived from 182,416 participants of European ancestry that included 2,441,815 autosomal single nucleotide polymorphisms (SNPs) provided by the Reproductive Genetics (ReproGen) Consortium (23). A set of SNPs that reached GWAS significance (*p* < 5 × 10^–8^) in association with AAM were selected as genetic instruments. SNPs with linkage disequilibrium (*r*^2^ < 0.001) were ruled out.

#### Selection of Genetic Instruments for Genetically Predicted Potential Mediators

We obtained SNPs for waist-to-hip ratio (WHR) from the Genetic Investigation of Anthropometric Traits (GIANT) consortium’s 2015 GWAS meta-analysis including 2,542,447 SNPs from 210,088 participants of European descent (24). SNPs for fasting blood glucose (FBG) were extracted from Meta-Analyses of Glucose and Insulin-related traits Consortium (MAGIC), which provided a GWAS meta-analysis of 2,445,760 SNPs from 46,186 participants of European descent in 2010 (25). SNPs for high-density lipoprotein (HDL) were obtained from a GWAS meta-analysis provided by Kettunen et al. (26) comprising 12,133,295 SNPs from 24,925 participants of European descent in 2016. SNPs for alcohol intake frequency, sleep duration, and age at which HRT was started were all acquired from an online public GWAS provided by Elsworth et al., and totaled 9,851,867 SNPs from participants of European descent in 2018. In addition, SNPs for current smoking behavior and systolic blood pressure (SBP) were selected from an online public GWAS provided by Neale et al., including 10,894,596 SNPs from participants of European descent in 2017. SNPs for hemoglobin A1c (HbA1c) were extracted from an online public GWAS provided by Neale et al. including 13,586,180 SNPs from participants of European descent in 2018. SNPs for triglycerides (TGs) were identified from an online public GWAS provided by Neale et al. comprised of 13,586,007 SNPs from participants of European descent in 2018. Detailed results of genetically predicted alcohol intake frequency, sleep duration, current smoking behavior, SBP, HbA1c, TGs, and age at which HRT was started could be obtained through online public GWAS database^1^ using the R 4.0.3 software TwoSampleMR package. The SNPs were clumped, and those with linkage disequilibrium (*r*^2^ < 0.001) were ruled out. SNPs that reached GWAS significance (*p* < 5 × 10^–8^) in association with potential mediators were selected as genetic instruments.

#### Selection of Genetic Instruments for Genetically Predicted Myocardial Infarction

For genetically predicted MI, we used genetic association estimates that were publicly available from the Coronary ARtery DIsease Genome wide Replication and Meta-analysis plus The Coronary Artery Disease Genetics Consortium (CARDIoGRAMplusC4D) 1000 Genomes-based GWAS meta-analysis of 123,504 controls and 60,801 cases including 8,600,000 SNPs (27). SNPs that reached GWAS significance (*p* < 5 × 10^–8^) in association with MI were selected as genetic instruments. The SNPs were clumped, and those with linkage disequilibrium (*r*^2^ < 0.001) were ruled out.

### Statistical Analysis

#### Association of Genetically Predicted Age at Menarche With Genetically Predicted Myocardial Infarction Risk

Two-sample MR analyses were performed to investigate the association of genetically predicted AAM with genetically predicted MI risk. We assumed that summarized GWAS data were available for multiple genetic variants that satisfied the following assumptions: (i) genetic variants were associated with exposure; (ii) genetic variants were independent of any confounder of the exposure-outcome association; (iii) genetic variants were independent of outcome and could only be associated with outcome through gene expression of exposure.

The genetic association of each effect allele with genetically predicted AAM was represented by X_*k*_ (*k* = 1, 2, 3…) with a standard error (SE) as σX_*k*_. X_*k*_ (*k* = 1, 2, 3…) representing the effect size per allele of SNP_*k*_ (*k* = 1, 2, 3…) in AAM (in years). The data of X_*k*_ and σX_*k*_ were both extracted from beta value and SE in a relevant GWAS study with SNP characteristics (23), respectively, by applying the R 4.0.3 software TwoSampleMR package. The genetic association of each allele with genetically predicted MI was represented by Y_*k*_ (*k* = 1, 2, 3….) with an SE as σY_*k*_. For a binary outcome as MI, Y_*k*_ (*k* = 1, 2, 3…) represented the effect size per allele of SNP_*k*_ (*k* = 1, 2, 3…) in the log-odds or the log probability of MI. The data of Y_*k*_ and σY_*k*_ were both extracted from OR and SE in a relevant GWAS study with SNP characteristics (27) and were harmonized, respectively, by applying the R 4.0.3 software TwoSampleMR package. The ratio estimate of the association of genetically predicted AAM with genetically predicted MI could be calculated by formula Y_*k*_/X_*k*_ (*k* = 1, 2, 3…). The SE of the ratio estimate could be approximated using the delta method, and the leading term is σY_*k*_/X_*k*_ (*k* = 1, 2, 3…) (28). To investigate the association of genetically predicted AAM with genetically predicted MI risk, an inverse variance weighted (IVW) meta-analysis using a fixed-effects model was implemented to pool estimates of MR as ${\hat{\beta }}_{\text{IVW}}$ across individual SNPs with the R 4.0.3 software (29). The calculation of ${\hat{\beta }}_{\text{IVW}}$ is shown by the following formula:


$${\hat{\beta }}_{\text{IVW}}=\frac{\Sigma_{k}\,X_{k}\,Y_{k}\,\sigma_{Y_{k}}^{-2}}{\Sigma_{k\,X_{k}}^{2}\,\sigma_{Y_{k}}^{-2}}$$

$s\,e\,\left({\hat{\beta }}_{\text{IVW}}\right)$, the approximate standard error of the pooled estimates, was also calculated with the R 4.0.3 software using the following formula:


$$s\,e\,\left({\hat{\beta }}_{\text{IVW}}\right)=\sqrt{\frac{1}{\Sigma_{k}\,X_{k}^{2}\,\sigma_{Y_{k}}^{-2}}}$$

#### Mediation With Genetically Predicted Lifestyles, Anthropometric and Cardiometabolic Parameters, and Age at Which Hormone Replacement Therapy Was Started

The genetic association of each effect allele with genetically determined potential mediators was represented by Z_*k*_ (*k* = 1, 2, 3….) with standard error (SE) σZ_*k*_. Z_*k*_ (*k* = 1, 2, 3…) representing the effect size per allele of SNP_*k*_ (*k* = 1, 2, 3…) in potential mediators. The data of Z_*k*_ and σZ_*k*_ were extracted from beta value and SE in relevant GWAS studies on potential mediators with SNP characteristics as mentioned above in section “Materials and Methods” and were harmonized, by applying the R 4.0.3 software TwoSampleMR package. To investigate the association of genetically predicted AAM with each genetically determined potential mediator, the IVW meta-analysis, which used a fixed-effects model, was also implemented through the R 4.0.3 software to pool estimates of MR as ${\hat{\beta }}_{{\mathrm{IVW}}^{*}}$ across individual SNPs. The calculation of ${\hat{\beta }}_{{\mathrm{IVW}}^{*}}$ is shown by the following formula:


$${\hat{\beta }}_{{\mathrm{IVW}}^{*}}=\frac{\Sigma_{k\,X_{k}\,Z_{k}}\,\sigma_{Z_{k}}^{-2}}{\Sigma_{k\,X_{k}}^{2}\,\sigma_{Z_{k}}^{-2}}$$

$S\,e\,\left({\hat{\beta }}_{{\mathrm{IVW}}^{*}}\right)$, the approximate standard error of the above pooled estimates, was calculated with R 4.0.3 software using the following formula:


$$s\,e\,\left({\hat{\beta }}_{{\mathrm{IVW}}^{*}}\right)=\sqrt{\frac{1}{\Sigma_{k\,X_{k}}^{2}\,\sigma_{Z_{k}}^{-2}}}$$

To determine the mediation of each factor, we multiplied coefficients of association of genetically predicted AAM with each mediator, and coefficients of association of each mediator with MI after adjusting for genetically predicted AAM. In order to estimate the association of t genetically determined potential mediators with genetically predicted risk of MI adjusting for influences from AAM, a regression-based MR was also conducted with the R 4.0.3 software (30).

#### Sensitivity Analyses

MR-Egger, MR-Egger intercept, and MR-PRESSO analyses were performed to estimate whether there was pleiotropy, which might affect the results of the MR analysis (30–32). The Robust Adjusted Profile Score (RAPS) was applied to test weak instrumental variables, and weighted median sensitivity analyses were conducted to estimate invalid instrumental variables (32).

#### Statistical Software

We carried out the two-step, two-sample MR analyses by utilizing R version 4.0.3 (R Foundation for Statistical Computing, Vienna, Austria, 2008) and R studio version 1.3.1093 (Boston, MA, United States). We used the TwoSampleMR package for R to facilitate the MR analyses.

## Results

### Association of Genetically Predicted Age at Menarche With Genetically Predicted Myocardial Infarction Risk

**FIGURE 1:**
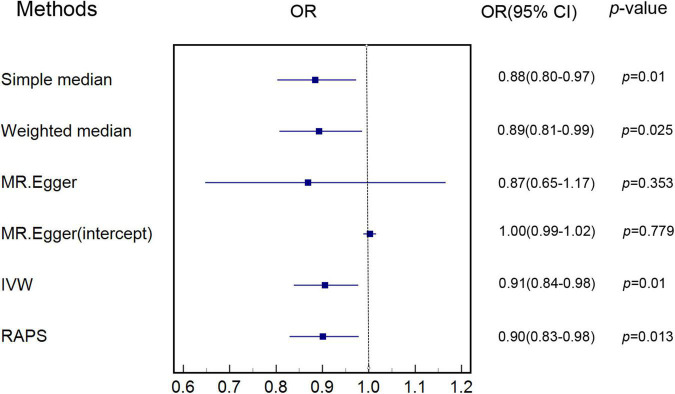
Estimate of associations of genetically predicted AAM with genetically predicted MI. IVW was applied as main analysis for estimating the association of genetically predicted AAM with genetically predicted MI. AAM, age at menarche; MI, myocardial infarction; IVW, inverse variance weighted.

Characteristics of SNPs used as instrumental variables for AAM are listed in Supplementary Table 1. Genetic estimates for the association of genetically predicted AAM with genetically predicted MI are shown in Supplementary Table 2. Figure 1 shows that increase in genetically predicted AAM was associated with lower risk of genetically predicted MI, with an odds ratio (OR) of 0.91 [95% confidence interval (CI) 0.84–0.98] in the main IVW MR analysis (also shown in Supplementary Table 3).

### Association of Genetically Predicted Age at Menarche With Genetically Predicted Lifestyles, Anthropometric and Cardiometabolic Parameters, and Age at Which Hormone Replacement Therapy Was Started

**FIGURE 2:**
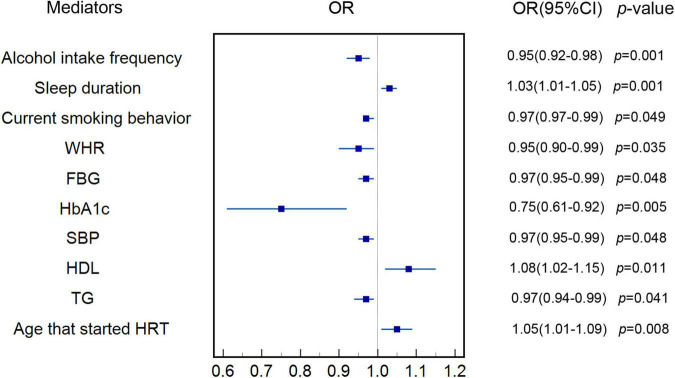
Estimate of the association of genetically predicted AAM with each genetically determined potential mediator. IVW was applied as main analysis. The results showed that increase in genetically predicted AAM was associated with genetically determined longer sleep duration, higher levels of HDL and older at which HRT was started. The results also showed that increase in genetically predicted AAM was inversely associated genetically determined alcohol intake frequency, current smoking behavior, WHR, FBG, HbA1c, SBP, and TGs. AAM, age at menarche; IVW, inverse variance weighted; HDL, high-density lipoprotein; HRT, hormone replacement therapy; WHR, waist-hip-ratio; FBG, fasting blood glucose; HbA1c, hemoglobin A1c; SBP, systolic blood pressure; TG, triglycerides.

Figure 2 indicates that increase in genetically predicted AAM was associated with lower genetically predicted alcohol intake frequency (OR: 0.95, 95% CI: 0.92–0.98, *p* < 0.001), current smoking behavior (OR: 0.99, 95% CI: 0.97–0.99, *p* = 0.049), WHR (OR: 0.95, 95% CI: 0.90–0.99, *p* = 0.035), FBG (OR: 0.97, 95% CI: 0.94–0.99, *p* = 0.048), HbA1c (OR: 0.75, 95% CI: 0.61–0.92, *p* = 0.005), SBP (OR: 0.97, 95% CI: 0.95–0.99, *p* = 0.048), and TGs (OR: 0.97, 95% CI: 0.94–0.99, *p* = 0.041) in the IVW MR analysis. Figure 2 also shows that increase in genetically predicted AAM is associated with genetically predicted longer time of sleep duration (OR: 1.03, 95% CI: 1.01–1.05, *p* = 0.001), higher levels of HDL (OR: 1.08, 95% CI: 1.02–1.15, *p* = 0.011), and older age at which HRT was started (OR: 1.05, 95% CI: 1.01–1.09, *p* = 0.008) in the IVW MR analysis. The above potential genetically predicted mediators that were associated with genetically predicted AAM (*p* < 0.05) were all involved in the MR analyses of association of each genetically predicted mediator with genetically predicted risk of MI. Characteristics of SNPs used as instrumental variables for genetically determined potential mediators are shown in Supplementary Tables 4–13. Genetic estimates for the association of genetically predicted AAM with genetically determined mediators can be seen in Supplementary Tables 14–23. MR estimates of associations of genetically predicted AAM with genetically determined risk factors are shown in Supplementary Table 24.

### Association of Genetically Predicted Lifestyles, Anthropometric and Cardiometabolic Parameters, and Age at Which Hormone Replacement Therapy Was Started With Genetically Predicted Risk of Myocardial Infarction

**FIGURE 3:**
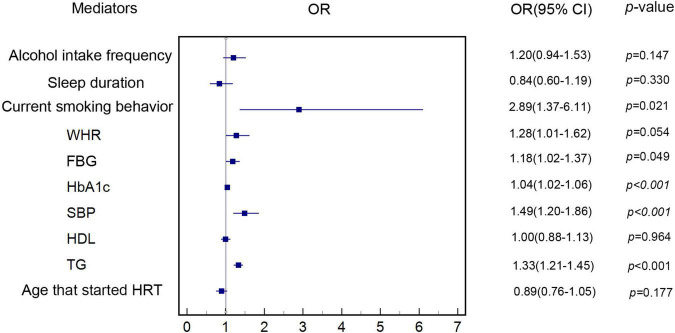
Estimate of the association of each genetically determined potential mediator with genetically predicted MI. The association of genetically determined potential mediators with genetically predicted MI was estimated by linear regression-based MR method. MI, myocardial infarction; HDL, high-density lipoprotein; HRT, hormone replacement therapy; WHR, waist-hip-ratio; FBG, fasting blood glucose; HbA1c, hemoglobin A1c; SBP, systolic blood pressure; TG, triglycerides.

Figure 3 shows that our MR analyses supported that genetically predicted increased risk of MI was associated with genetically predicted current smoking behavior (OR: 2.89, 95% CI: 1.37–6.11, *p* = 0.021) and genetically predicted higher levels of FBG (OR: 1.18, 95% CI: 1.02–1.37, *p* = 0.049), HbA1c (OR: 1.04, 95% CI: 1.02–1.06, *p* < 0.001), SBP (OR: 1.49, 95% CI: 1.20–1.86, *p* < 0.001), and TGs (OR: 1.33, 95% CI: 1.21–1.45, *p* < 0.001) in the IVW MR analysis, after adjusting for genetically predicted AAM. Because there was no evidence of association of genetically predicted alcohol intake frequency, sleep duration, WHR, HDL, and age at which HRT was started with genetically predicted MI risk in the IVW MR analysis (*p* > 0.05), these potential mediators were excluded. Genetic estimates for the association of genetically determined mediators with genetically predicted MI after adjusting for genetically predicted AAM are shown in Supplementary Tables 25–34.

### Mediation of Genetically Predicted Lifestyles and Cardiometabolic Parameters

The two-sample MR analyses showed that the percentage mediated by genetically predicted current smoking behavior and genetically predicted higher level of HbA1c, SBPs, and TGs was at a proportion of 13.9, 12.2, 10.5, and 9.2%, respectively (As is shown in Table 1). FBG was not involved in the final mediation analyses, because the mediation proportion of FBG was comparatively lower than the other four mediators, which indicated that it may not be a core mediator. The combined mediation proportion of the four risk factors accounted for 37.5% of the association of genetically predicted AAM with genetic predisposition to MI risk.

**TABLE 1:** MR analyses of association of genetically predicted AAM with genetically predicted risk of MI and mediation explained by risk factors.

	Beta	Proportion (%)
Estimated association of AAM with MI	−0.0990	1.0
**Estimated association of mediators with MI**		
Current smoking behavior	−0.0138	13.9
HbA1c	−0.0121	12.2
SBP	−0.0104	10.5
TG	−0.0091	9.2
Total	−0.0370	37.5

*MR, Mendelian randomization; AAM, age at menarche; MI, myocardial infarction; HbA1c, hemoglobin A1c; SBP, systolic blood pressure; TG, triglycerides.*

### Sensitivity Analyses

Simple median (*p* < 0.05, Supplementary Table 3) and weighted median sensitivity analyses (*p* < 0.05, Supplementary Table 3) showed that our results were less likely to be biased with invalid instruments. RAPS (*p* < 0.05, Supplementary Table 3) indicated that weak instruments were unlikely to affect our results. Other sensitivity analyses including single SNP analysis (Supplementary Table 35) and leave-one-out analysis (Supplementary Table 36) provided results consistent with the main IVW MR analysis. MR-Egger (*p* > 0.05, Supplementary Table 3) and MR-PRESSO analysis (*p* < 0.05, Supplementary Table 37) also showed that our MR analysis was not significantly affected by pleiotropy.

## Discussion

The MR analyses conducted in this study provided strong evidence to support the association of genetically predicted AAM with genetically predicted risk of MI. This study also found that the association was mediated by genetically predicted current smoking behavior and levels of HbA1c, SBP, and TGs at a proportion of 13.9, 12.2, 10.5, and 9.2%, respectively. Together, all these genetically determined mediators account for more than one-third of the association of genetically predicted AAM with genetically predicted risk of MI.

Our findings are consistent with previous studies conducted in different settings (5, 9, 33, 34). A hospital-based case-control study conducted by La Vecchia et al. (33) enrolled 576 women (202 cases of acute MI) below 55 years of age from 1983 to 1986 in Northern Italy and found that women with menarcheal age of 12–14 years showed reduced risk of MI (relative risk: 0.49, 95% CI: 0.31–0.75) compared with women whose menarche occurred younger than 12 years old. Another population-based prospective study enrolled 15,807 women aged 40–79 years from 1993 to 1997 and evaluated them for CVD events for 10.6 years, showing an elevated risk of incident CHD [1.23 (1.06–1.43)] in women with early AAM (<12 years) compared with those with later menarche (5). Gallagher et al. conducted a cohort study in Shanghai, China from 1989 to 1991 involving 267,400 women textile workers who were followed up through 2000. Their results indicated that women with menarche at 13 years or earlier had an increased risk of IHD with a hazard ratio (HR) = 1.44 (95% CI: 1–2.05) adjusted for age compared with those with later menarche at 15 years (8). In addition, a prospective study with 34,022 Chinese females aged 45–74 at enrollment (1993–1998) found a significant inverse association between menarcheal age and risk for CHD mortality across different AAM categories. HRs for CHD mortality of different age groups (≤12, 13–14, 15–16, ≥17 years old) were 1.06 (0.80–1.34), 1 (referent), 0.76 (0.65–0.9), and 72 (0.58–0.88), respectively (*p* trend < 0.001) (9). Cooper et al. also reported that the risk of IHD decreased with increasing age of menarche onset (age-adjusted RR 0.76 per year, 95% CI: 0.6–0.95) in a cohort study involving 867 White college-educated women (34).

Given the progress shown in our understanding of the association between AAM and MI based on recorded cases, previous observational studies were still limited by relatively small sample sizes and bias due to adjustment of various factors, which might have influenced the association of AAM with MI risk. Several MR studies with relatively large sample sizes had been undertaken to further genetically estimate the association of AAM with cardiovascular risk factors and cardiometabolic diseases (35, 36). Our results are also in accordance with Cao and Cui, who observed that later AAM decreased the risk of CHD (OR: 0.92, 95% CI: 0.88–0.96). Early AAM was found to be associated with the rising trend of HDL but lower diastolic blood pressure (DBP) combined with lower levels of TGs, log fasting insulin, log homeostasis model assessment of insulin resistance (HOMA-IR), and log homeostasis model assessment of β cell function (HOMA-β) (36). Another MR analysis from the Guangzhou Biobank Cohort Study, which investigated the association between AAM and cardiovascular risk factors, revealed that AAM was inversely correlated with levels of FBG (35). However, none of the previous observational or MR studies examined which genetically determined mediators influenced the association of genetically predicted AAM with genetically predicted MI and to what extent in an MR framework.

Recent studies have indicated that early AAM might not be a simple independent determining factor of cardiovascular adverse events but might reflect negative metabolic imprinting during the pre-pubescence period, such as relatively high weight or body mass index (BMI) during childhood (37–40). There was, thus, an imperative need to investigate whether genetically predicted increased AAM is associated with genetically predicted lower risk of MI among women during puberty or later adulthood through lifestyles and cardiometabolic or other potential mediators as long-term impacts of metabolic imprinting.

### Smoking

With regard to smoking behavior, Mueller et al. enrolled 34,022 Chinese women aged 45–74 years at baseline (1993–1998) and followed them prospectively throughout 2009. They found that increased AAM was associated with lower risk for CHD and CVD mortality among non-smoking females; HRs (95% CI) for CVD mortality in non-smoking females across different age categories of menarche (≤12, 13–14, 15–16, and ≥17 years old) were: 1.06 (0.87–1.29), 1 (referent), 0.89 (0.79–1), and 0.8 (0.69–0.93), respectively (*p* trend < 0.001), while for CHD mortality in non-smoking females the results were 1.06 (0.80–1.34), 1 (referent), 0.76 (0.65–0.9), and 0.72 (0.58–0.88), respectively (*p* trend < 0.001). However, no association was found between AAM and CHD or CVD mortality among smokers (9). Similarly, Gallagher et al. found that HRs (95% CI) for CVD mortality across different AAM groups (≤13, 14, 15, 16, and ≥17) were: 1.44 (1–2.05), 1.06 (0.76–1.47), 1 (referent), 1.09 (0.82–1.45), and 0.85 (0.65–1.12), respectively (*p* trend < 0.001) among non-smoking females vs. smokers (8).

The above results differ from the findings presented here, which showed that current smoking behavior might play a vital role in the association of genetically predicted AAM with genetically predicted MI. Consistent with our findings, Jacobsen made a similar point in his study, which suggested 1-year earlier AAM was associated with a mean 17.6% higher IHD mortality among women who smoked compared with 5.2% in women who never smoked (6). In addition, another cross-sectional study including 2,030 postmenopausal females 55–81 years old from England suggested that smoking behavior increased free estradiol significantly only in overweight postmenopausal females. These findings indicated that smoking-related increases in testosterone were translated into higher levels of estradiol through fat cells as an important peripheral conversion approach for postmenopausal overweight women. In the meantime, compared with lifetime smoking, current smoking behavior was found to significantly affect sex hormone levels; however, after a 1- to 2-year period of smoking cessation, sex hormone levels were the same as those of never smokers (41). Thus, relatively smaller sample size, low frequency of smoking among enrolled participants, lack of control of number of overweight women among enrolled participants, and inaccurate self-reported smoking time and cessation history might explain the different results between previous specific cohorts and our MR analyses. Given that sex hormone levels were associated with AAM, whether changes in current smoking behavior might modify CVD risks in a favorable direction through modification of endogenous sex hormones needs to be further investigated and results should be interpreted with caution.

### SBP/HbA1c/TGs

Another clear finding to emerge from our MR analyses was that SBP, HbA1c, and TGs played vital roles in mediating the association of early genetically predicted AAM with genetically predicted MI, which was consistent with the results from Remsberg et al., who found an inverse relationships among AAM and SBP (β estimates: −1.24, SE: 0.27), insulin (β estimates: −0.1, SE: 0.03), TGs (β estimates: −2.68, SE: 1.7) independent of fat-free mass and percent body fat during adolescence. Statistically significant associations were not found between AAM and glucose, total cholesterol, DBP, low-density lipoprotein, or HDL, which further supports the results from our MR analyses (12). In addition, another study that recruited 9,097 females aged 25–64 from China in 2004–2005 revealed that early AAM was associated with increased TGs, body fatness, and homeostasis model assessment of insulin resistance instead of FBG after adjusting for age (37). Lakshman et al. also reported that women with early AAM (<12 years) had a higher risk of hypertension [OR (95% CI):1.13 (1.02–1.24)] than those with later AAM in a prospective study that involved 15,807 females aged 40–79 years from 1993 to 1997 and followed for more than 10 years. Each 1-year delay of AAM was associated with 5% lower risk of hypertension (95% CI: 3–7%) in linear models, and hypertension risk could only be partially attenuated after adjusting for adult BMI and waist circumference (5).

The above studies support the finding that increased levels of HbA1c, which indicated that long-term hyperglycemia, in contrast to FBG, were more likely to mediate the association of early AAM with MI risk. Moreover, SBP may play a more crucial role in mediating the association of AAM with MI compared to DBP. Although both SBP and DBP could independently predict cardiovascular events, a cohort study involving 1.3 million adults from a general outpatient population suggested that SBP elevation (≥140 mmHg) had a greater effect on cardiovascular outcomes [HR (95% CI): 1.18 (1.17–1.18)] than DBP elevation [≥90 mmHg; HR (95% CI): 1.06 (1.06–1.07)], which further supported the findings of our MR analyses (42). Another interesting finding was that TGs, instead of other types of blood lipids, served a more essential role in mediation of the association of genetically predicted AAM with genetically predicted MI, which was further supported by results of the Newcastle Thousand Families Study, which recruited 1,142 children who were followed for 50 years. The study indicated that TGs were significantly associated with BMI at age 9 and were closely related with glucose metabolism in adult women after adjusting for adult percentage fat (43). Thus, the specific role of TGs and long-term impact on women need to be further investigated.

### Potential Mechanism

Although the inverse association of AAM and MI was supported by a number of previous cohort studies, several other studies provided a J shape or U shape association between AAM and CVD or CHD (44–46). These inconsistent results indicated that early or late AAM might lead to increased cardiovascular risk by triggering different risk factors or mediators. Lee et al. reported that early AAM was more likely to be related to overnutrition, psychosocial stress, and metabolic syndrome, and late AAM is usually due to polycystic ovary syndrome, undernutrition, and excessive exercise, which might be related to hypercortisolism and hypoestrogenism (13). Thus, SBP, HbA1c, and TGs, which are closely related to metabolic syndrome, might mediate the association of genetically predicted AAM with genetically predicted MI through oxidative stress causing vascular damage and disruption of plaques by chronic inflammation. Simultaneously, early AAM may lead to female precocious puberty and early onset of smoking, which could result in insulin resistance of adipocytes, vessel damage, and oxidative stress in adolescent girls and significant changes in sex hormones through adipocytes in postmenopausal overweight women as a long-term effect (47, 48). Further studies with big data and experimental evidence might be necessary to delineate the above possible mechanisms.

### Implications for Public Health and Clinical Management

Our study provides new insights from genetic estimates in an MR framework to further reveal the association of genetically predicted AAM with genetic predisposition to MI through a series of mediators that are closely related to metabolic syndrome. These innovative findings provided the potential targets for future pharmaceutical therapies and practical interventions such as lifestyle modifications on current smoking behavior and high-fat and high-sugar diets. Furthermore, our findings will also increase awareness of the importance of education for harmful habit cessation especially on girls during their early puberty to lower the MI risk by avoiding long duration of exposure to these cardiometabolic risk factors.

### Innovations and Limitations

Our study made several remarkable advances. The major strength of our study is that it presented an MR framework to genetically assess the association of AAM with MI and genetically estimate the potential mediation proportion of different risk factors in mediating this relationship. This study provides more comprehensive and stronger evidence for further exploring the pathogenic mechanism of early AAM in MI. Moreover, more than 647,920 participants were involved in the two-sample MR analyses, which had a larger sample size than previous observational studies and MR analyses. The greater statistical power of this study also complements the imprecision that might arise in one-sample MR analyses when estimating mediation proportions. In addition, as genetic variants were immutable, naturally and randomly allocated at conception, MR analyses provide robust estimates to reduce bias. The genetic instruments that represented AAM and the mediators investigate the lifetime rather than short-term effects.

Note that our MR analyses also might be somewhat limited in some respect. Even though the results of our MR analyses were sufficiently robust to estimate the association of genetically predicted AAM with genetically predicted MI, we believe that conducting prospective cohort studies in the future will enable us to provide valuable information about the association of AAM with MI from more comprehensive perspectives and help future researchers formulate a broader view for making clinical decisions and public health policies. Additionally, not all aspects of the exposure (AAM) phenotype can necessarily be deduced by these genetic variants. A notable example is that the genetic instruments for TG and SBP might capture average levels of TG and SBP but might not necessarily reflect the variability in serum lipid and blood pressure. The validity of our MR analyses relied on three key assumptions: (i) genetic variants were associated with AAM (exposure); (ii) genetic variants were independent of any confounder of AAM (exposure)-MI (outcome) association; (iii) genetic variants were only associated with MI (outcome) through gene expression instead of independent biological pathways (49). Because the second assumption was not easy to confirm because of potential unknown confounders, and the third assumption could be violated by pleiotropy, we performed various types of sensitivity analyses to further investigate whether the presence of pleiotropy, as well as weak instrumental variables and other invalid instrumental variables, influenced our results. The results of the sensitivity analysis were in accordance with those from our main IVW MR analysis. The analyses in this study were carried out on participants mostly of European descent, which may limit the generalizability of our findings to other ethnicities and populations.

## Conclusion

Our MR analyses indicate that increase in genetically predicted AAM was associated with lower risk of genetically predicted MI, and that this association was found at least partially mediated through current smoking behaviors, SBP, and levels of HbA1c and TGs. In other words, early AAM might reflect adverse metabolic imprinting during the pre-pubertal phase. Given the increasing number of adolescent females globally who are currently challenged by earlier AAM currently as a result of social-economic progress, this study may help public health policymakers and doctors formulate more scalable and effective strategies to reduce the incidence of MI due to early AAM without political and social reforms. Further interventional studies should be performed to examine whether controlling for the above mediators in adolescent females is effective in reducing their increased MI risk, and to develop a clearer picture of other related mediators as well as the interplay among them.

## Data Availability Statement

The datasets presented in this study can be found in online repositories. The names of the repository/repositories and accession number(s) can be found in the article/Supplementary Material.

## Author Contributions

JZ and KC designed the study, performed the statistical analyses, and drafted the manuscript. Y-DT, TH, and ED participated in the design of the study, helped to perform the statistical analyses, and critically revised the manuscript. CS, PL, JW, WW, KZ, XM, XW, JG, YL, and JS helped to revise the manuscript in detail. All authors approved the final version of the manuscript and ensured that all aspects of the study are complete and accurate.

## Conflict of Interest

The authors declare that the research was conducted in the absence of any commercial or financial relationships that could be construed as a potential conflict of interest.

## Publisher’s Note

All claims expressed in this article are solely those of the authors and do not necessarily represent those of their affiliated organizations, or those of the publisher, the editors and the reviewers. Any product that may be evaluated in this article, or claim that may be made by its manufacturer, is not guaranteed or endorsed by the publisher.

## Footnotes

1
https://gwas-api.mrcieu.ac.uk/
