# Supplementary material for: Genetically Determined Lifestyle and Cardiometabolic Risk Factors Mediate the Association of Genetically Predicted Age at Menarche With Genetic Predisposition to Myocardial Infarction: A Two-Step, Two-Sample Mendelian Randomization Study
Source: Front Cardiovasc Med. 2022 Apr 25;9:821068. doi: 10.3389/fcvm.2022.821068 (PMC9081496; doi:10.3389/fcvm.2022.821068)
Supplement: Supplementary file 1 [file Data_Sheet_1.docx]

**Supplemental Materials**

[Table S1. Characteristics of SNPs used as instrumental variables for genetically predicted AAM. 4](#_Toc96856753)

[Table S2. Genetic estimates for the association of genetically predicted AAM with genetically predicted MI. 10](#_Toc96856754)

[Table S3. IVW MR analysis and sensitivity analyses for estimate of the association of genetically predicted AAM with genetically predicted MI. 16](#_Toc96856755)

[Table S4. Characteristics of SNPs used as instrumental variables for genetically-determined current smoking behavior. 17](#_Toc96856756)

[Table S5. Characteristics of SNPs used as instrumental variables for genetically-determined HbA1c. 18](#_Toc96856757)

[Table S6. Characteristics of SNPs used as instrumental variables for genetically-determined SBP. 34](#_Toc96856758)

[Table S7. Characteristics of SNPs used as instrumental variables for genetically-determined TG. 47](#_Toc96856759)

[Table S8. Characteristics of SNPs used as instrumental variables for genetically-determined alcohol intake frequency. 66](#_Toc96856760)

[Table S9. Characteristics of SNPs used as instrumental variables for genetically-determined sleep duration. 74](#_Toc96856761)

[Table S10. Characteristics of SNPs used as instrumental variables for genetically-determined WHR. 80](#_Toc96856762)

[Table S11. Characteristics of SNPs used as instrumental variables for genetically-determined FBG. 83](#_Toc96856763)

[Table S12. Characteristics of SNPs used as instrumental variables for genetically-determined HDL. 84](#_Toc96856764)

[Table S13. Characteristics of SNPs used as instrumental variables for genetically-determined age that started HRT. 86](#_Toc96856765)

[Table S14. Genetic estimates for the association of genetically predicted AAM with genetically-determined current smoking behavior. 88](#_Toc96856766)

[Table S15. Genetic estimates for the association of genetically predicted AAM with genetically-determined HbA1c. 94](#_Toc96856767)

[Table S16. Genetic estimates for the association of genetically predicted AAM with genetically-determined SBP. 100](#_Toc96856768)

[Table S17. Genetic estimates for the association of genetically predicted AAM with genetically-determined TG. 106](#_Toc96856769)

[Table S18. Genetic estimates for the association of genetically predicted AAM with genetically-determined alcohol intake frequency. 112](#_Toc96856770)

[Table S19. Genetic estimates for the association of genetically predicted AAM with genetically-determined sleep duration. 118](#_Toc96856771)

[Table S20. Genetic estimates for the association of genetically predicted AAM with genetically-determined WHR. 124](#_Toc96856772)

[Table S21. Genetic estimates for the association of genetically predicted AAM with genetically-determined FBG. 130](#_Toc96856773)

[Table S22. Genetic estimates for the association of genetically predicted AAM with genetically-determined HDL. 136](#_Toc96856774)

[Table S23. Genetic estimates for the association of genetically predicted AAM with genetically-determined age that started HRT. 142](#_Toc96856775)

[Table S24. MR estimates of associations of genetically predicted AAM with each genetically-determined risk factor. 148](#_Toc96856776)

[Table S25. Genetic estimates for the association of genetically-determined current smoking behavior with genetically predicted MI after adjusting for genetically predicted AAM. 151](#_Toc96856777)

[Table S26. Genetic estimates for the association of genetically-determined HbA1c with genetically predicted MI after adjusting for genetically predicted AAM. 153](#_Toc96856778)

[Table S27. Genetic estimates for the association of genetically-determined SBP with genetically predicted MI after adjusting for genetically predicted AAM. 163](#_Toc96856779)

[Table S28. Genetic estimates for the association of genetically-determined TG with genetically predicted MI after adjusting for genetically predicted AAM. 173](#_Toc96856780)

[Table S29. Genetic estimates for the association of genetically-determined alcohol intake frequency with genetically predicted MI after adjusting for genetically predicted AAM. 185](#_Toc96856781)

[Table S30. Genetic estimates for the association of genetically-determined sleep duration with genetically predicted MI after adjusting for genetically predicted AAM. 192](#_Toc96856782)

[Table S31. Genetic estimates for the association of genetically-determined WHR with genetically predicted MI after adjusting for genetically predicted AAM. 197](#_Toc96856783)

[Table S32. Genetic estimates for the association of genetically-determined FBG with genetically predicted MI after adjusting for genetically predicted AAM. 200](#_Toc96856784)

[Table S33. Genetic estimates for the association of genetically-determined HDL with genetically predicted MI after adjusting for genetically predicted AAM. 202](#_Toc96856785)

[Table S34. Genetic estimates for the association of genetically-determined age that started HRT with genetically predicted MI after adjusting for genetically predicted AAM. 204](#_Toc96856786)

[Table S35. Single SNP analysis for estimate of the association of genetically predicted AAM with genetically predicted MI. 206](#_Toc96856787)

[Table S36. Leave-one-out analysis for estimate of the association of genetically predicted AAM with genetically predicted MI. 212](#_Toc96856788)

[Table S37. MR-PRESSO analysis for estimate of the association of genetically predicted AAM with genetically predicted MI. 217](#_Toc96856789)

## Table S1. Characteristics of SNPs used as instrumental variables for genetically predicted AAM.

|  | SNP | Effect allele | Other allele | EAF | X_k_^a^ | σX_k_^b^ | *P*-value | F-statistics |
| --- | --- | --- | --- | --- | --- | --- | --- | --- |
| 1 | rs2344508 | A | G | 0.56 | 0.034 | 0.005747 | 3.30E-09 | 34.99828183 |
| 2 | rs466639 | C | T | 0.87 | 0.075 | 0.008706 | 7.00E-18 | 74.21661875 |
| 3 | rs618678 | T | C | 0.33 | -0.034 | 0.006148 | 3.20E-08 | 30.58231361 |
| 4 | rs633715 | C | T | 0.2 | -0.051 | 0.00725 | 2.00E-12 | 49.48394768 |
| 5 | rs6694738 | A | C | 0.18 | -0.044 | 0.007885 | 2.40E-08 | 31.14062281 |
| 6 | rs6747380 | A | G | 0.17 | 0.065 | 0.007658 | 2.10E-17 | 72.04821259 |
| 7 | rs6758290 | C | T | 0.51 | -0.04 | 0.006322 | 2.50E-10 | 40.0306958 |
| 8 | rs1518080 | G | C | 0.43 | -0.051 | 0.006004 | 2.00E-17 | 72.14463039 |
| 9 | rs4369815 | G | T | 0.05 | -0.08 | 0.011888 | 1.70E-11 | 45.28888279 |
| 10 | rs895526 | C | T | 0.8 | 0.044 | 0.007553 | 5.70E-09 | 33.93447105 |
| 11 | rs2947411 | G | A | 0.87 | -0.052 | 0.007655 | 1.10E-11 | 46.14170137 |
| 12 | rs2687729 | G | A | 0.23 | 0.044 | 0.006575 | 2.20E-11 | 44.78415736 |
| 13 | rs6770162 | A | G | 0.44 | 0.036 | 0.005733 | 3.40E-10 | 39.4302016 |
| 14 | rs3870341 | G | A | 0.69 | -0.043 | 0.006449 | 2.60E-11 | 44.45698801 |
| 15 | rs11715566 | T | C | 0.51 | 0.052 | 0.005631 | 2.60E-20 | 85.27227854 |
| 16 | rs3914188 | C | G | 0.74 | 0.044 | 0.006702 | 5.20E-11 | 43.1004942 |
| 17 | rs7642134 | G | A | 0.56 | 0.038 | 0.005912 | 1.30E-10 | 41.30851441 |
| 18 | rs17351680 | G | C | 0.13 | 0.044 | 0.007557 | 5.80E-09 | 33.90062132 |
| 19 | rs1482853 | A | C | 0.39 | -0.038 | 0.006043 | 3.20E-10 | 39.5485896 |
| 20 | rs10938397 | G | A | 0.43 | -0.038 | 0.005923 | 1.40E-10 | 41.16371137 |
| 21 | rs3733632 | G | A | 0.23 | 0.049 | 0.007862 | 4.60E-10 | 38.84004112 |
| 22 | rs9997604 | C | A | 0.7 | 0.039 | 0.006511 | 2.10E-09 | 35.87857266 |
| 23 | rs9647570 | G | T | 0.12 | 0.046 | 0.008427 | 4.80E-08 | 29.79595481 |
| 24 | rs740077 | C | A | 0.21 | -0.046 | 0.007045 | 6.60E-11 | 42.63400525 |
| 25 | rs13179411 | T | G | 0.16 | 0.06 | 0.007813 | 1.60E-14 | 58.97122796 |
| 26 | rs3115627 | G | A | 0.4 | 0.038 | 0.006453 | 3.90E-09 | 34.67299602 |
| 27 | rs13215865 | T | C | 0.15 | -0.042 | 0.007472 | 1.90E-08 | 31.59411941 |
| 28 | rs2153127 | C | T | 0.46 | -0.077 | 0.002077 | 1.00E-200 | 1373.869167 |
| 29 | rs4840086 | G | A | 0.48 | -0.036 | 0.00562 | 1.50E-10 | 41.0288266 |
| 30 | rs11756454 | A | T | 0.45 | 0.034 | 0.005703 | 2.50E-09 | 35.53901616 |
| 31 | rs2179786 | T | G | 0.32 | -0.039 | 0.00572 | 9.20E-12 | 46.49182974 |
| 32 | rs9373571 | A | T | 0.56 | 0.034 | 0.005926 | 9.60E-09 | 32.92063868 |
| 33 | rs2184968 | C | T | 0.41 | -0.036 | 0.005672 | 2.20E-10 | 40.28044631 |
| 34 | rs6933660 | A | C | 0.3 | -0.036 | 0.006331 | 1.30E-08 | 32.33131228 |
| 35 | rs1079866 | G | C | 0.12 | 0.072 | 0.008444 | 1.50E-17 | 72.71225827 |
| 36 | rs11767400 | A | C | 0.31 | 0.035 | 0.006295 | 2.70E-08 | 30.91196995 |
| 37 | rs7821178 | A | C | 0.41 | -0.045 | 0.006196 | 3.80E-13 | 52.74396456 |
| 38 | rs888345 | A | G | 0.82 | -0.044 | 0.007315 | 1.80E-09 | 36.17899564 |
| 39 | rs2617056 | T | A | 0.39 | -0.036 | 0.005933 | 1.30E-09 | 36.81332683 |
| 40 | rs4242496 | A | T | 0.5 | -0.033 | 0.005696 | 6.90E-09 | 33.56274094 |
| 41 | rs1516883 | A | G | 0.3 | -0.091 | 0.002455 | 1.00E-200 | 1373.877306 |
| 42 | rs913588 | A | G | 0.55 | -0.034 | 0.005683 | 2.20E-09 | 35.78794847 |
| 43 | rs12003641 | T | C | 0.07 | 0.082 | 0.010555 | 7.90E-15 | 60.36043713 |
| 44 | rs7853970 | C | T | 0.57 | -0.037 | 0.00626 | 3.40E-09 | 34.94010137 |
| 45 | rs1874984 | C | G | 0.44 | 0.037 | 0.006005 | 7.20E-10 | 37.96574175 |
| 46 | rs7103411 | T | C | 0.78 | -0.043 | 0.007056 | 1.10E-09 | 37.13905474 |
| 47 | rs7944630 | A | G | 0.57 | 0.047 | 0.005728 | 2.30E-16 | 67.32706727 |
| 48 | rs10840031 | A | G | 0.24 | 0.038 | 0.006406 | 3.00E-09 | 35.18383358 |
| 49 | rs12291726 | G | A | 0.11 | 0.057 | 0.008333 | 7.90E-12 | 46.79035378 |
| 50 | rs11022756 | C | A | 0.74 | -0.048 | 0.006481 | 1.30E-13 | 54.85139918 |
| 51 | rs16938437 | T | C | 0.05 | -0.067 | 0.010348 | 9.50E-11 | 41.92149532 |
| 52 | rs7119712 | A | G | 0.19 | -0.041 | 0.006445 | 2.00E-10 | 40.46668343 |
| 53 | rs9565073 | C | T | 0.52 | 0.034 | 0.005881 | 7.40E-09 | 33.4267287 |
| 54 | rs9555810 | G | C | 0.26 | 0.047 | 0.006492 | 4.50E-13 | 52.41183053 |
| 55 | rs10144321 | G | A | 0.22 | -0.042 | 0.006618 | 2.20E-10 | 40.28038545 |
| 56 | rs10483727 | C | T | 0.64 | -0.037 | 0.005785 | 1.60E-10 | 40.90267197 |
| 57 | rs12148769 | A | G | 0.12 | -0.055 | 0.009752 | 1.70E-08 | 31.81020705 |
| 58 | rs3743266 | C | T | 0.33 | -0.045 | 0.006233 | 5.20E-13 | 52.12784768 |
| 59 | rs12915845 | T | C | 0.42 | -0.035 | 0.005729 | 1.00E-09 | 37.32485713 |
| 60 | rs1659127 | A | G | 0.3 | 0.044 | 0.006372 | 5.00E-12 | 47.68701809 |
| 61 | rs12598642 | G | A | 0.44 | 0.044 | 0.005681 | 9.50E-15 | 59.99694907 |
| 62 | rs9939609 | A | T | 0.45 | -0.042 | 0.00572 | 2.10E-13 | 53.90896192 |
| 63 | rs9635759 | A | G | 0.33 | 0.058 | 0.006362 | 7.70E-20 | 83.12574655 |
| 64 | rs1398217 | C | G | 0.58 | 0.046 | 0.005767 | 1.50E-15 | 63.63162227 |
| 65 | rs2303100 | T | C | 0.55 | 0.038 | 0.005684 | 2.30E-11 | 44.69705617 |
| 66 | rs4801589 | G | C | 0.43 | 0.032 | 0.005628 | 1.30E-08 | 32.33137611 |
| 67 | rs852069 | G | A | 0.62 | 0.036 | 0.005907 | 1.10E-09 | 37.13897286 |
| 68 | rs2836950 | G | C | 0.32 | -0.035 | 0.006206 | 1.70E-08 | 31.81015113 |

SNPs: Single nucleotide polymorphisms; AAM: Age at menarche; EAF: Effect allele frequency; SE：Standard error. F-statistics= (Effect/SE)^2^

^a^ Effect size per allele in AAM (in years)

^b^ Standard error of the genetic association of each effect allele with genetically predicted AAM

Table S2. Genetic estimates for the association of genetically predicted AAM with genetically predicted MI.

|  | SNPs | Effect allele | X_k_^a^ | σX_k_^b^ | Y_k_ ^c^ | σY_k_ ^d^ |
| --- | --- | --- | --- | --- | --- | --- |
| 1 | rs10144321 | G | -0.042 | 0.006618 | 0.008671 | 0.012078 |
| 2 | rs10483727 | C | -0.037 | 0.005785 | 0.020817 | 0.010598 |
| 3 | rs1079866 | G | 0.072 | 0.008444 | 0.010109 | 0.014712 |
| 4 | rs10840031 | A | 0.038 | 0.006406 | -0.00831 | 0.011034 |
| 5 | rs10938397 | G | -0.038 | 0.005923 | 0.022473 | 0.010345 |
| 6 | rs11022756 | C | -0.048 | 0.006481 | 0.034106 | 0.011348 |
| 7 | rs11715566 | T | 0.052 | 0.005631 | -0.01126 | 0.010206 |
| 8 | rs11756454 | A | 0.034 | 0.005703 | -0.01131 | 0.010182 |
| 9 | rs11767400 | A | 0.035 | 0.006295 | -0.00763 | 0.012338 |
| 10 | rs12003641 | T | 0.082 | 0.010555 | -0.01636 | 0.017105 |
| 11 | rs12148769 | A | -0.055 | 0.009752 | -0.01288 | 0.016633 |
| 12 | rs12291726 | G | 0.057 | 0.008333 | -0.03578 | 0.012923 |
| 13 | rs12598642 | G | 0.044 | 0.005681 | -0.02195 | 0.010668 |
| 14 | rs12915845 | T | -0.035 | 0.005729 | 0.003662 | 0.010614 |
| 15 | rs13179411 | T | 0.06 | 0.007813 | 0.007297 | 0.015377 |
| 16 | rs13215865 | T | -0.042 | 0.007472 | -0.00587 | 0.014387 |
| 17 | rs1398217 | C | 0.046 | 0.005767 | -0.00272 | 0.010282 |
| 18 | rs1482853 | A | -0.038 | 0.006043 | 0.01563 | 0.010696 |
| 19 | rs1516883 | A | -0.091 | 0.002455 | 0.003408 | 0.011156 |
| 20 | rs1518080 | G | -0.051 | 0.006004 | 0.010725 | 0.010587 |
| 21 | rs1659127 | A | 0.044 | 0.006372 | -0.00428 | 0.011338 |
| 22 | rs16938437 | T | -0.067 | 0.010348 | 0.023337 | 0.017954 |
| 23 | rs17351680 | G | 0.044 | 0.007557 | -0.03242 | 0.014302 |
| 24 | rs1874984 | C | 0.037 | 0.006005 | 0.006292 | 0.010634 |
| 25 | rs2153127 | C | -0.077 | 0.002077 | 0.016059 | 0.010489 |
| 26 | rs2179786 | T | -0.039 | 0.00572 | 0.007768 | 0.010325 |
| 27 | rs2184968 | C | -0.036 | 0.005672 | -0.03391 | 0.010648 |
| 28 | rs2303100 | T | 0.038 | 0.005684 | -0.00477 | 0.010467 |
| 29 | rs2344508 | A | 0.034 | 0.005747 | 0.004186 | 0.010372 |
| 30 | rs2617056 | T | -0.036 | 0.005933 | 0.004999 | 0.01142 |
| 31 | rs2687729 | G | 0.044 | 0.006575 | 0.015564 | 0.01143 |
| 32 | rs2836950 | G | -0.035 | 0.006206 | -0.01041 | 0.010699 |
| 33 | rs2947411 | G | -0.052 | 0.007655 | 0.008336 | 0.013304 |
| 34 | rs3733632 | G | 0.049 | 0.007862 | -0.00951 | 0.012618 |
| 35 | rs3743266 | C | -0.045 | 0.006233 | 0.014377 | 0.011535 |
| 36 | rs3870341 | G | -0.043 | 0.006449 | -0.01288 | 0.012718 |
| 37 | rs3914188 | C | 0.044 | 0.006702 | -0.00073 | 0.01171 |
| 38 | rs4242496 | A | -0.033 | 0.005696 | 0.016421 | 0.011176 |
| 39 | rs4369815 | G | -0.08 | 0.011888 | 0.033404 | 0.022874 |
| 40 | rs466639 | C | 0.075 | 0.008706 | 0.001761 | 0.014643 |
| 41 | rs4801589 | G | 0.032 | 0.005628 | 0.004878 | 0.010269 |
| 42 | rs4840086 | G | -0.036 | 0.00562 | 0.013269 | 0.010346 |
| 43 | rs618678 | T | -0.034 | 0.006148 | -0.00568 | 0.011329 |
| 44 | rs633715 | C | -0.051 | 0.00725 | 0.02877 | 0.012958 |
| 45 | rs6694738 | A | -0.044 | 0.007885 | -0.00683 | 0.013923 |
| 46 | rs6747380 | A | 0.065 | 0.007658 | 0.029116 | 0.013281 |
| 47 | rs6758290 | C | -0.04 | 0.006322 | 0.001158 | 0.010519 |
| 48 | rs6770162 | A | 0.036 | 0.005733 | 0.003953 | 0.01068 |
| 49 | rs6933660 | A | -0.036 | 0.006331 | -0.00471 | 0.010842 |
| 50 | rs7103411 | T | -0.043 | 0.007056 | 0.027164 | 0.012082 |
| 51 | rs7119712 | A | -0.041 | 0.006445 | 0.012709 | 0.012647 |
| 52 | rs740077 | C | -0.046 | 0.007045 | -0.00424 | 0.011868 |
| 53 | rs7642134 | G | 0.038 | 0.005912 | 0.008629 | 0.01044 |
| 54 | rs7821178 | A | -0.045 | 0.006196 | -0.01153 | 0.010597 |
| 55 | rs7853970 | C | -0.037 | 0.00626 | 0.020347 | 0.010659 |
| 56 | rs7944630 | A | 0.047 | 0.005728 | 0.011748 | 0.010472 |
| 57 | rs852069 | G | 0.036 | 0.005907 | -0.00617 | 0.010631 |
| 58 | rs888345 | A | -0.044 | 0.007315 | -0.01156 | 0.012805 |
| 59 | rs895526 | C | 0.044 | 0.007553 | -0.0089 | 0.01346 |
| 60 | rs913588 | A | -0.034 | 0.005683 | 0.013862 | 0.01038 |
| 61 | rs9373571 | A | 0.034 | 0.005926 | 0.008282 | 0.010637 |
| 62 | rs9555810 | G | 0.047 | 0.006492 | -0.00184 | 0.011521 |
| 63 | rs9565073 | C | 0.034 | 0.005881 | 0.008895 | 0.010603 |
| 64 | rs9635759 | A | 0.058 | 0.006362 | -0.01379 | 0.012186 |
| 65 | rs9647570 | G | 0.046 | 0.008427 | 0.017301 | 0.013834 |
| 66 | rs9939609 | A | -0.042 | 0.00572 | 0.024013 | 0.010729 |
| 67 | rs9997604 | C | 0.039 | 0.006511 | 0.022968 | 0.011822 |

AAM: Age at menarche; MI: Myocardial infarction; SNPs: Single nucleotide polymorphisms.

^a^ Effect size per allele of SNP_k_ (k=1,2,3…) in AAM (in years)

^b^ Standard error of the genetic association of each effect allele with genetically predicted AAM

^c^ Effect size per allele of SNP_k_ (k=1,2,3…) in the log-odds or the log probability of MI

^d^ Standard error of the genetic association of each effect allele with genetically predicted MI

Table S3. IVW MR analysis and sensitivity analyses for estimate of the association of genetically predicted AAM with genetically predicted MI.

| Method | OR | 95%CI | *p*-value |
| --- | --- | --- | --- |
| Simple median | 0.88 | 0.80-0.97 | 0.010 |
| Weighted median | 0.89 | 0.81-0.98 | 0.025 |
| MR Egger | 0.87 | 0.65-1.17 | 0.353 |
| MR Egger intercept | 0.002 | 0.007 | 0.779 |
| IVW MR analysis | 0.91 | 0.84-0.98 | 0.010 |
| Robust adjusted profile score | 0.90 | 0.83-0.98 | 0.013 |

IVW: Inverse variance weighted; MR: Mendelian randomization; AAM: Age at menarche; MI: Myocardial infarction; OR: Odds ratio; CI: Confidence interval;

## Table S4. Characteristics of SNPs used as instrumental variables for genetically-determined current smoking behavior.

|  | SNP | Effect allele | Other allele | EAF | Z_k_^a^ | σZ_k_^b^ | *P*-value | F-statistics |
| --- | --- | --- | --- | --- | --- | --- | --- | --- |
| 1 | rs11210887 | A | G | 0.700334 | -0.00955 | 0.001441 | 3.46E-11 | 43.89903 |
| 2 | rs3773814 | C | A | 0.153285 | 0.010087 | 0.001838 | 4.04E-08 | 30.13051 |
| 3 | rs11096777 | C | T | 0.182487 | 0.009903 | 0.001713 | 7.37E-09 | 33.43698 |
| 4 | rs1549214 | C | T | 0.640579 | -0.00903 | 0.001378 | 5.62E-11 | 42.9501 |
| 5 | rs7726560 | T | G | 0.438569 | 0.007457 | 0.001333 | 2.20E-08 | 31.31223 |
| 6 | rs7807019 | G | A | 0.460379 | 0.007598 | 0.001327 | 1.03E-08 | 32.79024 |
| 7 | rs62474713 | A | G | 0.498591 | -0.00868 | 0.001324 | 5.60E-11 | 42.9596 |
| 8 | rs17730481 | A | G | 0.323033 | 0.008563 | 0.001422 | 1.71E-09 | 36.27694 |
| 9 | rs73227362 | C | A | 0.195714 | -0.00982 | 0.001665 | 3.67E-09 | 34.79314 |
| 10 | rs113382419 | A | C | 0.11211 | 0.021268 | 0.002099 | 4.00E-24 | 102.6664 |
| 11 | rs10891481 | G | A | 0.382581 | 0.008446 | 0.001363 | 5.82E-10 | 38.38346 |
| 12 | rs8033799 | C | A | 0.211359 | 0.008913 | 0.001622 | 3.90E-08 | 30.20126 |
| 13 | rs56113850 | C | T | 0.577736 | -0.01141 | 0.001337 | 1.37E-17 | 72.90358 |
| 14 | rs4809542 | G | C | 0.070307 | 0.015336 | 0.002593 | 3.32E-09 | 34.98634 |
| 15 | rs6062496 | A | G | 0.544683 | 0.007318 | 0.001326 | 3.39E-08 | 30.47388 |
| 16 | rs9607805 | T | C | 0.727753 | 0.009444 | 0.001489 | 2.26E-10 | 40.22853 |

SNP: Single nucleotide polymorphisms; EAF: Effect allele frequency; SE：Standard error. F-statistics= (Effect/SE)^2^

^a^ Effect size of each allele on genetically-determined current smoking behavior (pack-years per allele)

^b^ Standard error of the genetic association of each effect allele with genetically-determined current smoking behavior

## Table S5. Characteristics of SNPs used as instrumental variables for genetically-determined HbA1c.

|  | SNP | Effect allele | Other allele | EAF | Z_k_^a^ | σZ_k_^b^ | *P*-value | F-statistics |
| --- | --- | --- | --- | --- | --- | --- | --- | --- |
| 1 | rs3927482 | G | T | 0.22404 | 0.12184 | 0.020197 | 1.62E-09 | 36.39201 |
| 2 | rs115340137 | C | T | 0.008882 | -0.60097 | 0.081779 | 2.01E-13 | 54.00351 |
| 3 | rs2487569 | T | A | 0.11067 | 0.15133 | 0.024673 | 8.60E-10 | 37.6189 |
| 4 | rs348330 | A | G | 0.63441 | -0.11927 | 0.015983 | 8.52E-14 | 55.68598 |
| 5 | rs9701805 | C | G | 0.089535 | -0.22065 | 0.026826 | 1.95E-16 | 67.6544 |
| 6 | rs7528296 | C | T | 0.3863 | -0.09937 | 0.015818 | 3.34E-10 | 39.46696 |
| 7 | rs1044145 | C | T | 0.54025 | 0.085473 | 0.015431 | 3.04E-08 | 30.68102 |
| 8 | rs7513688 | A | G | 0.36059 | 0.10972 | 0.016014 | 7.33E-12 | 46.94312 |
| 9 | rs34440965 | TA | T | 0.70607 | -0.13281 | 0.0171 | 8.09E-15 | 60.32111 |
| 10 | rs12136762 | G | A | 0.50348 | -0.09448 | 0.016457 | 9.40E-09 | 32.96143 |
| 11 | rs11204535 | G | A | 0.26676 | -0.10312 | 0.017401 | 3.11E-09 | 35.11862 |
| 12 | rs1175550 | G | A | 0.23081 | -0.17719 | 0.01848 | 9.04E-22 | 91.93365 |
| 13 | rs201886961 | T | C | 0.28077 | -0.11206 | 0.01727 | 8.68E-11 | 42.10334 |
| 14 | rs857721 | A | T | 0.26691 | 0.37763 | 0.017303 | 1.58E-105 | 476.3105 |
| 15 | rs11580608 | T | C | 0.31639 | 0.12292 | 0.016481 | 8.78E-14 | 55.62602 |
| 16 | rs198325 | T | C | 0.22163 | -0.12324 | 0.01847 | 2.52E-11 | 44.52148 |
| 17 | rs340882 | G | C | 0.62158 | 0.18618 | 0.015886 | 1.02E-31 | 137.3526 |
| 18 | rs560887 | C | T | 0.70132 | 0.40038 | 0.016716 | 1.14E-126 | 573.6936 |
| 19 | rs7572278 | A | T | 0.20375 | 0.11723 | 0.019068 | 7.86E-10 | 37.79786 |
| 20 | rs838717 | A | G | 0.56555 | 0.10064 | 0.0155 | 8.42E-11 | 42.15779 |
| 21 | rs72781658 | G | A | 0.13461 | 0.20186 | 0.022514 | 3.09E-19 | 80.38874 |
| 22 | rs780093 | C | T | 0.61816 | 0.15748 | 0.015777 | 1.85E-23 | 99.63271 |
| 23 | rs77981966 | T | C | 0.076555 | -0.36057 | 0.028944 | 1.30E-35 | 155.1894 |
| 24 | rs143667358 | T | TATACATACATAC | 0.19569 | 0.13058 | 0.019519 | 2.24E-11 | 44.75465 |
| 25 | rs6726007 | T | A | 0.46539 | -0.10831 | 0.015378 | 1.88E-12 | 49.60636 |
| 26 | rs4972439 | C | T | 0.22005 | -0.14035 | 0.018551 | 3.88E-14 | 57.23876 |
| 27 | rs45504994 | G | C | 0.46745 | -0.09728 | 0.015389 | 2.60E-10 | 39.95597 |
| 28 | rs13019832 | A | G | 0.41735 | -0.12765 | 0.015588 | 2.64E-16 | 67.05958 |
| 29 | rs2943640 | C | A | 0.6518 | 0.1491 | 0.016089 | 1.92E-20 | 85.88102 |
| 30 | rs1496653 | G | A | 0.20435 | -0.13138 | 0.019011 | 4.84E-12 | 47.75827 |
| 31 | rs113851927 | GT | G | 0.31224 | 0.16937 | 0.016665 | 2.92E-24 | 103.291 |
| 32 | rs10865977 | T | C | 0.62465 | -0.08881 | 0.015835 | 2.04E-08 | 31.45343 |
| 33 | rs11708067 | G | A | 0.24549 | -0.26157 | 0.01782 | 9.22E-49 | 215.4569 |
| 34 | rs1981767 | A | G | 0.2775 | 0.095852 | 0.017197 | 2.50E-08 | 31.06683 |
| 35 | rs6777684 | G | A | 0.60967 | 0.13445 | 0.01579 | 1.68E-17 | 72.50323 |
| 36 | rs9826367 | G | A | 0.44872 | -0.17191 | 0.015431 | 8.01E-29 | 124.1121 |
| 37 | rs6785881 | T | C | 0.4812 | -0.10646 | 0.015374 | 4.38E-12 | 47.95117 |
| 38 | rs1905505 | A | G | 0.28363 | -0.19336 | 0.017013 | 6.31E-30 | 129.1729 |
| 39 | rs13063578 | A | T | 0.40027 | 0.10195 | 0.015652 | 7.36E-11 | 42.42626 |
| 40 | rs6784925 | T | C | 0.55167 | -0.0934 | 0.015409 | 1.35E-09 | 36.74048 |
| 41 | rs9866749 | T | A | 0.70539 | 0.1395 | 0.017252 | 6.20E-16 | 65.3837 |
| 42 | rs4611812 | T | C | 0.40428 | -0.0987 | 0.015682 | 3.10E-10 | 39.61162 |
| 43 | rs12497133 | A | G | 0.53317 | 0.098225 | 0.015458 | 2.09E-10 | 40.37732 |
| 44 | rs56330132 | CTCA | C | 0.18642 | 0.14506 | 0.019847 | 2.70E-13 | 53.42021 |
| 45 | rs12633493 | T | C | 0.58662 | 0.11715 | 0.015626 | 6.54E-14 | 56.20681 |
| 46 | rs4689394 | G | C | 0.59864 | 0.14488 | 0.015673 | 2.39E-20 | 85.45011 |
| 47 | rs28690107 | C | G | 0.70867 | -0.1245 | 0.018514 | 1.76E-11 | 45.22079 |
| 48 | rs4698874 | C | T | 0.48539 | -0.10502 | 0.015359 | 8.05E-12 | 46.75393 |
| 49 | rs28504375 | A | T | 0.63745 | -0.23087 | 0.016693 | 1.72E-43 | 191.2785 |
| 50 | rs34835465 | C | T | 0.070365 | -0.17232 | 0.030144 | 1.09E-08 | 32.67906 |
| 51 | rs146302237 | AT | A | 0.13331 | -0.17036 | 0.022956 | 1.16E-13 | 55.07352 |
| 52 | rs6878122 | A | G | 0.68018 | -0.13225 | 0.016459 | 9.39E-16 | 64.56311 |
| 53 | rs6885132 | G | C | 0.099832 | -0.18425 | 0.025997 | 1.37E-12 | 50.23062 |
| 54 | rs145762933 | A | G | 0.050019 | 0.21073 | 0.035389 | 2.61E-09 | 35.45816 |
| 55 | rs7720275 | C | T | 0.17005 | 0.12178 | 0.02043 | 2.51E-09 | 35.53164 |
| 56 | rs67131976 | T | C | 0.17231 | 0.28395 | 0.020297 | 1.86E-44 | 195.7132 |
| 57 | rs998584 | A | C | 0.48165 | 0.12424 | 0.015384 | 6.73E-16 | 65.22053 |
| 58 | rs12210538 | G | A | 0.23618 | -0.12788 | 0.018075 | 1.50E-12 | 50.05514 |
| 59 | rs6920313 | C | T | 0.30591 | 0.095312 | 0.016651 | 1.04E-08 | 32.76533 |
| 60 | rs9258357 | C | T | 0.83632 | 0.16138 | 0.020723 | 6.84E-15 | 60.64488 |
| 61 | rs9264277 | C | T | 0.63199 | 0.15204 | 0.015963 | 1.67E-21 | 90.71659 |
| 62 | rs1131114 | C | T | 0.2651 | 0.19015 | 0.017397 | 8.39E-28 | 119.4659 |
| 63 | rs9273363 | A | C | 0.30378 | 0.289 | 0.016685 | 3.53E-67 | 300.0152 |
| 64 | rs11759026 | G | A | 0.22712 | 0.13598 | 0.01838 | 1.38E-13 | 54.73424 |
| 65 | rs62440928 | A | G | 0.60464 | -0.11048 | 0.015875 | 3.42E-12 | 48.43283 |
| 66 | rs78588343 | A | G | 0.17915 | -0.1647 | 0.020022 | 1.94E-16 | 67.66628 |
| 67 | rs1800562 | A | G | 0.078843 | -0.5108 | 0.028482 | 6.90E-72 | 321.6332 |
| 68 | rs6901903 | C | T | 0.27105 | 0.16167 | 0.017719 | 7.27E-20 | 83.24928 |
| 69 | rs9376091 | T | C | 0.26036 | -0.17628 | 0.01755 | 9.77E-24 | 100.8909 |
| 70 | rs376563 | C | T | 0.53313 | 0.096419 | 0.01539 | 3.73E-10 | 39.25075 |
| 71 | rs60238952 | G | A | 0.21923 | -0.10404 | 0.018626 | 2.33E-08 | 31.2005 |
| 72 | rs76323047 | G | A | 0.11843 | 0.17521 | 0.023796 | 1.81E-13 | 54.2138 |
| 73 | rs34518086 | CT | C | 0.53976 | -0.09224 | 0.015626 | 3.58E-09 | 34.84218 |
| 74 | rs17168486 | T | C | 0.17349 | 0.16157 | 0.020347 | 2.01E-15 | 63.05517 |
| 75 | rs10487796 | A | T | 0.45053 | -0.16103 | 0.015411 | 1.49E-25 | 109.1822 |
| 76 | rs76702117 | G | A | 0.040151 | 0.2271 | 0.039114 | 6.40E-09 | 33.71086 |
| 77 | rs1708302 | T | C | 0.50097 | -0.15899 | 0.015348 | 3.83E-25 | 107.309 |
| 78 | rs1004558 | T | C | 0.17898 | 0.44709 | 0.020003 | 1.39E-110 | 499.5738 |
| 79 | rs6953344 | G | A | 0.24663 | 0.10983 | 0.017821 | 7.16E-10 | 37.982 |
| 80 | rs5888710 | CT | C | 0.34724 | -0.10772 | 0.016104 | 2.25E-11 | 44.74301 |
| 81 | rs12668254 | G | A | 0.079671 | -0.20599 | 0.028435 | 4.35E-13 | 52.47903 |
| 82 | rs77258375 | CT | C | 0.36037 | 0.12124 | 0.01614 | 5.85E-14 | 56.42672 |
| 83 | rs2737263 | T | G | 0.28427 | -0.11708 | 0.01701 | 5.87E-12 | 47.37583 |
| 84 | rs35859536 | T | C | 0.31344 | -0.2774 | 0.01656 | 5.85E-63 | 280.603 |
| 85 | rs2954021 | G | A | 0.50565 | 0.095504 | 0.015352 | 4.95E-10 | 38.7002 |
| 86 | rs66593272 | T | A | 0.037563 | -0.54055 | 0.040328 | 5.88E-41 | 179.6629 |
| 87 | rs4977200 | G | A | 0.61379 | -0.09701 | 0.015963 | 1.22E-09 | 36.93286 |
| 88 | rs7820334 | T | C | 0.31109 | -0.10923 | 0.016643 | 5.28E-11 | 43.07454 |
| 89 | rs4737010 | A | G | 0.22802 | 0.33164 | 0.018313 | 2.90E-73 | 327.9554 |
| 90 | rs73565707 | G | A | 0.16528 | -0.1499 | 0.020641 | 3.82E-13 | 52.7402 |
| 91 | rs35467189 | CT | C | 0.69364 | 0.094866 | 0.016751 | 1.49E-08 | 32.07301 |
| 92 | rs61750929 | T | C | 0.055145 | -0.43693 | 0.033677 | 1.75E-38 | 168.3282 |
| 93 | rs10733564 | G | T | 0.86481 | -0.12746 | 0.022684 | 1.92E-08 | 31.57247 |
| 94 | rs10811660 | A | G | 0.17332 | -0.30869 | 0.020265 | 2.25E-52 | 232.0341 |
| 95 | rs1929915 | A | G | 0.2095 | 0.14594 | 0.018823 | 8.99E-15 | 60.11336 |
| 96 | rs550057 | T | C | 0.25461 | 0.23038 | 0.017649 | 6.22E-39 | 170.3921 |
| 97 | rs28641468 | C | T | 0.75584 | 0.17046 | 0.017815 | 1.09E-21 | 91.55315 |
| 98 | rs1574285 | T | G | 0.60147 | -0.09232 | 0.01569 | 4.02E-09 | 34.61924 |
| 99 | rs10758658 | A | G | 0.19951 | -0.13066 | 0.019186 | 9.77E-12 | 46.37847 |
| 100 | rs2796441 | A | G | 0.42001 | -0.09636 | 0.015513 | 5.25E-10 | 38.58433 |
| 101 | rs2002905 | A | C | 0.22518 | 0.1819 | 0.018426 | 5.56E-23 | 97.45481 |
| 102 | rs34872471 | C | T | 0.29127 | 0.47526 | 0.016882 | 3.61E-174 | 792.5282 |
| 103 | rs142196758 | A | G | 0.014002 | -1.2439 | 0.068451 | 9.35E-74 | 330.2261 |
| 104 | rs7077479 | A | C | 0.33125 | 0.10373 | 0.016555 | 3.71E-10 | 39.26001 |
| 105 | rs11257655 | T | C | 0.20825 | 0.21882 | 0.018898 | 5.32E-31 | 134.0733 |
| 106 | rs4745982 | G | T | 0.079637 | -1.0375 | 0.029209 | 1.00E-200 | 1261.661 |
| 107 | rs7898054 | T | C | 0.38032 | -0.18945 | 0.015808 | 4.35E-33 | 143.6268 |
| 108 | rs61850681 | A | G | 0.23828 | -0.16614 | 0.018084 | 4.06E-20 | 84.4033 |
| 109 | rs555895 | G | T | 0.35197 | 0.10458 | 0.016063 | 7.49E-11 | 42.3881 |
| 110 | rs2305196 | A | G | 0.2647 | -0.15241 | 0.017432 | 2.28E-18 | 76.44208 |
| 111 | rs12419995 | T | A | 0.63117 | -0.1227 | 0.016956 | 4.63E-13 | 52.36514 |
| 112 | rs2237895 | C | A | 0.41525 | 0.17623 | 0.015527 | 7.51E-30 | 128.8204 |
| 113 | rs147208676 | T | C | 0.081564 | 0.15993 | 0.028231 | 1.47E-08 | 32.09278 |
| 114 | rs174549 | A | G | 0.30997 | -0.10537 | 0.016576 | 2.06E-10 | 40.40866 |
| 115 | rs3020069 | A | G | 0.68286 | 0.093396 | 0.01649 | 1.48E-08 | 32.07859 |
| 116 | rs7105853 | A | G | 0.12674 | 0.13366 | 0.023019 | 6.39E-09 | 33.71553 |
| 117 | rs7124355 | G | A | 0.67508 | -0.1 | 0.016451 | 1.21E-09 | 36.94786 |
| 118 | rs11039165 | G | A | 0.27103 | -0.13326 | 0.017253 | 1.13E-14 | 59.65823 |
| 119 | rs11602873 | T | A | 0.15759 | -0.18717 | 0.021037 | 5.76E-19 | 79.15983 |
| 120 | rs10830963 | G | C | 0.27517 | 0.29735 | 0.017141 | 2.22E-67 | 300.9287 |
| 121 | rs12365580 | A | G | 0.15047 | -0.12988 | 0.021566 | 1.72E-09 | 36.26982 |
| 122 | rs415895 | G | C | 0.64515 | -0.13765 | 0.016036 | 9.21E-18 | 73.68182 |
| 123 | rs1787663 | A | C | 0.29016 | 0.10095 | 0.016889 | 2.27E-09 | 35.72768 |
| 124 | rs4760682 | A | C | 0.81301 | 0.3561 | 0.019665 | 2.95E-73 | 327.911 |
| 125 | rs2258238 | T | A | 0.10482 | 0.19058 | 0.025119 | 3.28E-14 | 57.56387 |
| 126 | rs76895963 | G | T | 0.021026 | -1.0374 | 0.058949 | 2.76E-69 | 309.6989 |
| 127 | rs10842987 | C | A | 0.20089 | -0.1624 | 0.019136 | 2.13E-17 | 72.02275 |
| 128 | rs10784889 | T | C | 0.58987 | -0.08716 | 0.015611 | 2.36E-08 | 31.17474 |
| 129 | rs77530740 | T | A | 0.058589 | -0.1937 | 0.032747 | 3.32E-09 | 34.98777 |
| 130 | rs73226260 | A | G | 0.034445 | -0.23455 | 0.042635 | 3.77E-08 | 30.26484 |
| 131 | rs117709065 | T | C | 0.017646 | -0.37667 | 0.060928 | 6.33E-10 | 38.21978 |
| 132 | rs4238013 | T | C | 0.79599 | -0.11913 | 0.01927 | 6.33E-10 | 38.21896 |
| 133 | rs11065979 | T | C | 0.43738 | -0.10915 | 0.015494 | 1.86E-12 | 49.62727 |
| 134 | rs1215468 | G | A | 0.29294 | -0.13265 | 0.016939 | 4.85E-15 | 61.3252 |
| 135 | rs12876143 | C | T | 0.088704 | 0.34765 | 0.026984 | 5.68E-38 | 165.9861 |
| 136 | rs8000868 | T | C | 0.15829 | 0.16963 | 0.020974 | 6.10E-16 | 65.4098 |
| 137 | rs368865 | G | A | 0.72505 | 0.1045 | 0.017151 | 1.11E-09 | 37.12391 |
| 138 | rs8002606 | G | C | 0.16767 | 0.17541 | 0.020599 | 1.66E-17 | 72.51309 |
| 139 | rs6602900 | G | C | 0.2209 | -0.16892 | 0.018688 | 1.59E-19 | 81.70272 |
| 140 | rs229587 | T | C | 0.32452 | 0.12193 | 0.016377 | 9.70E-14 | 55.43097 |
| 141 | rs4905989 | A | G | 0.8373 | -0.14001 | 0.020779 | 1.61E-11 | 45.40136 |
| 142 | rs72681698 | C | T | 0.011004 | -0.41066 | 0.073465 | 2.27E-08 | 31.24668 |
| 143 | rs10873398 | A | G | 0.64641 | -0.09381 | 0.016088 | 5.52E-09 | 33.99829 |
| 144 | rs7151822 | C | T | 0.24754 | -0.15893 | 0.017827 | 4.90E-19 | 79.47952 |
| 145 | rs4516170 | C | T | 0.04993 | -0.26401 | 0.035198 | 6.36E-14 | 56.26065 |
| 146 | rs12912009 | A | G | 0.72643 | -0.14568 | 0.017227 | 2.77E-17 | 71.51227 |
| 147 | rs34715063 | C | T | 0.1297 | 0.132 | 0.02295 | 8.84E-09 | 33.08129 |
| 148 | rs1550026 | T | A | 0.40767 | 0.10777 | 0.015667 | 6.04E-12 | 47.31775 |
| 149 | rs12910361 | G | A | 0.71349 | 0.1614 | 0.016961 | 1.81E-21 | 90.55327 |
| 150 | rs72802358 | C | G | 0.10168 | -0.15063 | 0.025538 | 3.68E-09 | 34.78958 |
| 151 | rs2925979 | C | T | 0.6991 | -0.11956 | 0.016725 | 8.80E-13 | 51.1022 |
| 152 | rs7193384 | G | C | 0.18126 | -0.15264 | 0.019983 | 2.21E-14 | 58.34657 |
| 153 | rs1121980 | A | G | 0.42204 | 0.15492 | 0.015526 | 1.92E-23 | 99.5625 |
| 154 | rs4889490 | T | G | 0.39039 | -0.09002 | 0.015729 | 1.05E-08 | 32.75414 |
| 155 | rs551118 | G | C | 0.57809 | 0.22727 | 0.015781 | 5.20E-47 | 207.4031 |
| 156 | rs181204 | G | A | 0.33299 | 0.11418 | 0.016399 | 3.35E-12 | 48.47807 |
| 157 | rs8050500 | C | T | 0.44602 | -0.11337 | 0.015435 | 2.06E-13 | 53.94895 |
| 158 | rs7196917 | G | A | 0.43176 | 0.095553 | 0.015504 | 7.13E-10 | 37.98404 |
| 159 | rs12448999 | A | T | 0.57972 | -0.08609 | 0.01556 | 3.16E-08 | 30.60877 |
| 160 | rs9901806 | T | C | 0.58304 | -0.08609 | 0.01568 | 4.02E-08 | 30.14136 |
| 161 | rs594398 | C | G | 0.51943 | 0.086064 | 0.015375 | 2.18E-08 | 31.33378 |
| 162 | rs3834968 | AG | A | 0.2224 | 0.31649 | 0.0186 | 6.64E-65 | 289.5304 |
| 163 | rs8068844 | C | T | 0.34071 | 0.097508 | 0.016183 | 1.69E-09 | 36.30466 |
| 164 | rs371297493 | AT | A | 0.47101 | -0.09051 | 0.015826 | 1.07E-08 | 32.70848 |
| 165 | rs10908278 | A | T | 0.51534 | -0.12471 | 0.01543 | 6.38E-16 | 65.32369 |
| 166 | rs10853047 | C | T | 0.14524 | 0.13064 | 0.021923 | 2.54E-09 | 35.51014 |
| 167 | rs12451511 | G | T | 0.19536 | 0.10704 | 0.019364 | 3.24E-08 | 30.55639 |
| 168 | rs2263122 | G | T | 0.53736 | -0.27571 | 0.015386 | 9.01E-72 | 321.1098 |
| 169 | rs7206953 | C | T | 0.7368 | -0.1082 | 0.017398 | 5.01E-10 | 38.67728 |
| 170 | rs12602109 | A | G | 0.23551 | -0.15085 | 0.018779 | 9.54E-16 | 64.52763 |
| 171 | rs12600858 | A | G | 0.22395 | -0.1311 | 0.01843 | 1.13E-12 | 50.60049 |
| 172 | rs1641523 | T | C | 0.56645 | -0.12694 | 0.015571 | 3.58E-16 | 66.46056 |
| 173 | rs9900803 | T | C | 0.69138 | -0.16892 | 0.016657 | 3.67E-24 | 102.8415 |
| 174 | rs1995138 | A | G | 0.075947 | 0.17294 | 0.028937 | 2.28E-09 | 35.71773 |
| 175 | rs56397034 | C | G | 0.39134 | -0.11569 | 0.015683 | 1.63E-13 | 54.41684 |
| 176 | rs33978622 | C | G | 0.33313 | -0.11579 | 0.016391 | 1.62E-12 | 49.90352 |
| 177 | rs75372982 | G | A | 0.28732 | -0.10827 | 0.017165 | 2.84E-10 | 39.78585 |
| 178 | rs34358015 | TA | T | 0.70595 | 0.10535 | 0.017024 | 6.10E-10 | 38.29533 |
| 179 | rs12984096 | C | G | 0.39175 | 0.21315 | 0.015735 | 8.54E-42 | 183.5005 |
| 180 | rs7507912 | A | G | 0.41897 | -0.13184 | 0.015743 | 5.57E-17 | 70.13251 |
| 181 | rs55966194 | G | C | 0.28404 | -0.13601 | 0.017023 | 1.36E-15 | 63.83656 |
| 182 | rs4809556 | A | G | 0.59545 | -0.08715 | 0.015637 | 2.50E-08 | 31.06189 |
| 183 | rs67897819 | A | G | 0.21621 | 0.10557 | 0.019365 | 5.00E-08 | 29.71981 |
| 184 | rs1883932 | T | A | 0.50792 | -0.10275 | 0.015364 | 2.27E-11 | 44.72549 |
| 185 | rs6014993 | G | A | 0.48674 | 0.10483 | 0.015391 | 9.71E-12 | 46.3914 |
| 186 | rs13042148 | T | C | 0.15498 | 0.11857 | 0.021217 | 2.30E-08 | 31.2307 |
| 187 | rs6518681 | G | A | 0.907019 | 0.14997 | 0.026571 | 1.66E-08 | 31.85613 |
| 188 | rs855791 | G | A | 0.56146 | -0.25462 | 0.015509 | 1.51E-60 | 269.5364 |
| 189 | rs117721418 | T | C | 0.081009 | -0.1915 | 0.028111 | 9.62E-12 | 46.40716 |
| 190 | rs2143918 | C | A | 0.471 | -0.12093 | 0.015384 | 3.82E-15 | 61.79162 |

SNP: Single nucleotide polymorphisms; HbA1c: Hemoglobin A1c; EAF: Effect allele frequency; SE：Standard error. F-statistics= (Effect/SE)^2^

^a^ Effect size of each allele on genetically-determined HbA1c (percentage point per allele)

^b^ Standard error of the genetic association of each effect allele with genetically-determined HbA1c

## Table S6. Characteristics of SNPs used as instrumental variables for genetically-determined SBP.

|  | SNP | Effect allele | Other allele | EAF | Z_k_^a^ | σZ_k_^b^ | *P*-value | F-statistics |
| --- | --- | --- | --- | --- | --- | --- | --- | --- |
| 1 | rs2076328 | T | G | 0.500298 | -0.01405 | 0.002466 | 1.23E-08 | 32.44634 |
| 2 | rs2493296 | T | C | 0.137053 | 0.024408 | 0.003582 | 9.49E-12 | 46.43506 |
| 3 | rs3790604 | A | C | 0.073854 | 0.036549 | 0.004712 | 8.71E-15 | 60.17368 |
| 4 | rs4639796 | A | G | 0.159456 | 0.019328 | 0.003356 | 8.44E-09 | 33.17242 |
| 5 | rs2004776 | T | C | 0.240432 | 0.019916 | 0.002882 | 4.82E-12 | 47.76274 |
| 6 | rs17535443 | A | G | 0.27653 | -0.02169 | 0.002754 | 3.41E-15 | 62.02279 |
| 7 | rs71654213 | T | C | 0.38459 | -0.01713 | 0.002554 | 1.99E-11 | 44.9861 |
| 8 | rs786910 | G | C | 0.588893 | -0.01633 | 0.002499 | 6.27E-11 | 42.73826 |
| 9 | rs6690557 | G | T | 0.284079 | -0.01511 | 0.002728 | 3.03E-08 | 30.68818 |
| 10 | rs488834 | T | C | 0.766564 | -0.01687 | 0.002904 | 6.24E-09 | 33.75911 |
| 11 | rs55857306 | A | G | 0.163974 | -0.04376 | 0.003323 | 1.39E-39 | 173.3658 |
| 12 | rs35479618 | A | G | 0.01735 | 0.069691 | 0.009386 | 1.13E-13 | 55.12609 |
| 13 | rs6732308 | G | A | 0.208384 | -0.01823 | 0.003046 | 2.18E-09 | 35.80692 |
| 14 | rs12694861 | A | G | 0.604693 | 0.013713 | 0.002511 | 4.76E-08 | 29.81544 |
| 15 | rs116734066 | T | C | 0.092508 | -0.02785 | 0.004265 | 6.59E-11 | 42.63992 |
| 16 | rs6734118 | A | C | 0.2163 | -0.01896 | 0.002993 | 2.40E-10 | 40.11536 |
| 17 | rs268263 | A | T | 0.75424 | 0.030114 | 0.002881 | 1.46E-25 | 109.2269 |
| 18 | rs1250259 | A | T | 0.735684 | -0.01885 | 0.002795 | 1.57E-11 | 45.45145 |
| 19 | rs17759661 | A | C | 0.498101 | 0.017636 | 0.002461 | 7.66E-13 | 51.3721 |
| 20 | rs6733889 | C | T | 0.623065 | -0.01505 | 0.002537 | 2.99E-09 | 35.19233 |
| 21 | rs2249105 | G | A | 0.361063 | -0.01803 | 0.002555 | 1.68E-12 | 49.82712 |
| 22 | rs1438898 | C | A | 0.251051 | 0.015766 | 0.002848 | 3.10E-08 | 30.64645 |
| 23 | rs1047891 | A | C | 0.315673 | -0.01612 | 0.002646 | 1.10E-09 | 37.13434 |
| 24 | rs79349366 | T | C | 0.032519 | -0.04076 | 0.006998 | 5.74E-09 | 33.92163 |
| 25 | rs6544667 | T | C | 0.383035 | -0.01393 | 0.002532 | 3.82E-08 | 30.23923 |
| 26 | rs1530558 | C | T | 0.136004 | -0.02269 | 0.003587 | 2.54E-10 | 39.99987 |
| 27 | rs2892796 | A | G | 0.077703 | -0.027 | 0.0046 | 4.36E-09 | 34.45899 |
| 28 | rs12714414 | C | T | 0.160321 | -0.01966 | 0.00356 | 3.36E-08 | 30.4895 |
| 29 | rs35021474 | G | C | 0.617081 | -0.0224 | 0.002536 | 1.03E-18 | 78.01076 |
| 30 | rs13016772 | T | C | 0.761577 | 0.016147 | 0.002892 | 2.37E-08 | 31.16434 |
| 31 | rs34727427 | C | T | 0.318561 | 0.01478 | 0.00264 | 2.16E-08 | 31.34359 |
| 32 | rs6768542 | A | G | 0.158366 | -0.02054 | 0.003395 | 1.45E-09 | 36.60471 |
| 33 | rs3796205 | C | G | 0.351128 | -0.01726 | 0.002585 | 2.46E-11 | 44.56798 |
| 34 | rs3817581 | T | C | 0.493028 | 0.018524 | 0.002463 | 5.44E-14 | 56.57082 |
| 35 | rs2447607 | T | C | 0.617609 | 0.017354 | 0.002538 | 8.01E-12 | 46.76546 |
| 36 | rs2643826 | T | C | 0.4525 | 0.015996 | 0.002477 | 1.07E-10 | 41.69042 |
| 37 | rs743395 | T | C | 0.374686 | 0.014613 | 0.002585 | 1.58E-08 | 31.95321 |
| 38 | rs35593046 | T | G | 0.269679 | -0.01635 | 0.002814 | 6.23E-09 | 33.76258 |
| 39 | rs262986 | A | G | 0.467816 | -0.01377 | 0.002477 | 2.68E-08 | 30.92449 |
| 40 | rs17011002 | G | C | 0.1407 | 0.028501 | 0.003551 | 1.02E-15 | 64.40123 |
| 41 | rs1229984 | C | T | 0.977549 | 0.05189 | 0.008257 | 3.29E-10 | 39.49426 |
| 42 | rs73098804 | A | T | 0.129962 | 0.021141 | 0.003688 | 9.87E-09 | 32.86837 |
| 43 | rs7439366 | C | T | 0.454524 | 0.015315 | 0.002469 | 5.52E-10 | 38.48802 |
| 44 | rs10857147 | T | A | 0.292191 | 0.037123 | 0.002713 | 1.35E-42 | 187.1751 |
| 45 | rs4835266 | C | T | 0.483575 | -0.01481 | 0.0025 | 3.13E-09 | 35.10125 |
| 46 | rs10029530 | T | A | 0.658635 | -0.01903 | 0.002597 | 2.40E-13 | 53.65332 |
| 47 | rs231708 | C | G | 0.686651 | -0.01476 | 0.00265 | 2.55E-08 | 31.02675 |
| 48 | rs28667801 | T | A | 0.406256 | 0.013983 | 0.002515 | 2.72E-08 | 30.902 |
| 49 | rs2102397 | C | A | 0.498839 | -0.0178 | 0.002464 | 5.09E-13 | 52.17626 |
| 50 | rs12656497 | C | T | 0.596357 | 0.031728 | 0.002508 | 1.11E-36 | 160.0804 |
| 51 | rs158172 | G | A | 0.219112 | -0.01743 | 0.002974 | 4.60E-09 | 34.35333 |
| 52 | rs17056301 | C | T | 0.254256 | 0.018768 | 0.002833 | 3.46E-11 | 43.89964 |
| 53 | rs62368019 | C | T | 0.290266 | 0.015966 | 0.002714 | 4.04E-09 | 34.60784 |
| 54 | rs709668 | G | A | 0.797922 | 0.01783 | 0.003072 | 6.49E-09 | 33.68293 |
| 55 | rs17677603 | G | A | 0.394587 | 0.017989 | 0.002528 | 1.12E-12 | 50.62139 |
| 56 | rs13154549 | G | A | 0.067583 | 0.030453 | 0.004909 | 5.51E-10 | 38.49054 |
| 57 | rs1644318 | C | T | 0.384598 | 0.019146 | 0.002539 | 4.63E-14 | 56.88641 |
| 58 | rs13436194 | G | A | 0.425995 | -0.01855 | 0.002486 | 8.49E-14 | 55.69459 |
| 59 | rs6911827 | T | C | 0.454272 | 0.014685 | 0.00247 | 2.76E-09 | 35.34684 |
| 60 | rs1925148 | G | A | 0.560365 | -0.01397 | 0.00248 | 1.78E-08 | 31.72635 |
| 61 | rs2971608 | C | T | 0.221343 | 0.020858 | 0.002975 | 2.35E-12 | 49.16788 |
| 62 | rs210630 | G | A | 0.500672 | -0.01553 | 0.002462 | 2.85E-10 | 39.77789 |
| 63 | rs62434129 | T | A | 0.071464 | -0.03048 | 0.004781 | 1.82E-10 | 40.65279 |
| 64 | rs9385405 | C | G | 0.435707 | 0.022515 | 0.002475 | 9.43E-20 | 82.73681 |
| 65 | rs9349379 | G | A | 0.404893 | -0.01381 | 0.002505 | 3.55E-08 | 30.38506 |
| 66 | rs75391241 | G | A | 0.072076 | 0.028158 | 0.004761 | 3.33E-09 | 34.9801 |
| 67 | rs9294987 | C | T | 0.502206 | 0.013856 | 0.002479 | 2.29E-08 | 31.23559 |
| 68 | rs1052486 | G | A | 0.474786 | 0.019453 | 0.002463 | 2.88E-15 | 62.35602 |
| 69 | rs9476307 | G | A | 0.410838 | -0.0144 | 0.002503 | 8.75E-09 | 33.103 |
| 70 | rs7753358 | A | T | 0.487051 | 0.014439 | 0.002503 | 7.97E-09 | 33.2856 |
| 71 | rs7765526 | G | A | 0.541864 | -0.01419 | 0.002496 | 1.30E-08 | 32.33882 |
| 72 | rs57301765 | A | G | 0.154163 | 0.024666 | 0.00339 | 3.45E-13 | 52.93926 |
| 73 | rs6461992 | G | A | 0.926979 | 0.035501 | 0.00473 | 6.16E-14 | 56.32526 |
| 74 | rs10269774 | A | G | 0.323543 | -0.02078 | 0.002631 | 2.85E-15 | 62.37556 |
| 75 | rs62481856 | A | G | 0.196947 | 0.043879 | 0.00309 | 9.56E-46 | 201.6194 |
| 76 | rs17173238 | G | A | 0.28611 | 0.015442 | 0.002721 | 1.38E-08 | 32.21408 |
| 77 | rs2721800 | C | G | 0.180727 | -0.01812 | 0.003224 | 1.90E-08 | 31.59634 |
| 78 | rs1543270 | T | C | 0.455016 | -0.01586 | 0.002475 | 1.47E-10 | 41.06791 |
| 79 | rs7798991 | C | T | 0.262473 | 0.0157 | 0.002799 | 2.03E-08 | 31.46827 |
| 80 | rs891511 | A | G | 0.319374 | -0.01797 | 0.002666 | 1.59E-11 | 45.41778 |
| 81 | rs1813742 | T | C | 0.588185 | 0.018487 | 0.0025 | 1.42E-13 | 54.68762 |
| 82 | rs34896506 | C | T | 0.176744 | 0.021064 | 0.003223 | 6.32E-11 | 42.7204 |
| 83 | rs2977334 | T | G | 0.594551 | 0.016376 | 0.002508 | 6.64E-11 | 42.62531 |
| 84 | rs2469997 | C | G | 0.815428 | -0.0196 | 0.003172 | 6.44E-10 | 38.18577 |
| 85 | rs4736135 | T | C | 0.71422 | -0.0169 | 0.002751 | 8.13E-10 | 37.73039 |
| 86 | rs73563812 | T | G | 0.235901 | -0.02106 | 0.0029 | 3.85E-13 | 52.72528 |
| 87 | rs6983129 | A | C | 0.522069 | -0.0198 | 0.002479 | 1.39E-15 | 63.79503 |
| 88 | rs6271 | T | C | 0.073838 | -0.02886 | 0.004699 | 8.24E-10 | 37.70484 |
| 89 | rs10817007 | G | T | 0.128175 | 0.022057 | 0.00368 | 2.06E-09 | 35.92152 |
| 90 | rs2177843 | T | C | 0.146396 | 0.023575 | 0.003496 | 1.55E-11 | 45.47294 |
| 91 | rs10883948 | T | G | 0.503542 | -0.01354 | 0.00246 | 3.71E-08 | 30.29913 |
| 92 | rs57946343 | C | T | 0.15191 | -0.02953 | 0.003426 | 6.75E-18 | 74.29823 |
| 93 | rs56092448 | T | C | 0.114447 | 0.022238 | 0.003859 | 8.28E-09 | 33.20922 |
| 94 | rs12258967 | G | C | 0.297673 | -0.03144 | 0.002693 | 1.73E-31 | 136.3177 |
| 95 | rs11527181 | G | A | 0.433889 | -0.01837 | 0.002489 | 1.61E-13 | 54.43087 |
| 96 | rs2274224 | C | G | 0.432255 | -0.02466 | 0.002482 | 2.93E-23 | 98.72381 |
| 97 | rs1779240 | A | G | 0.763859 | -0.01893 | 0.002902 | 6.89E-11 | 42.553 |
| 98 | rs10883543 | T | G | 0.887598 | 0.031002 | 0.003921 | 2.65E-15 | 62.51898 |
| 99 | rs11191580 | C | T | 0.076747 | -0.0511 | 0.004608 | 1.42E-28 | 122.989 |
| 100 | rs2782980 | C | T | 0.721774 | 0.020191 | 0.002741 | 1.75E-13 | 54.27526 |
| 101 | rs604723 | C | T | 0.724339 | 0.033938 | 0.00277 | 1.64E-34 | 150.1423 |
| 102 | rs4937515 | C | G | 0.594982 | -0.02023 | 0.002523 | 1.06E-15 | 64.32845 |
| 103 | rs55925664 | A | T | 0.185723 | 0.025681 | 0.00316 | 4.44E-16 | 66.03799 |
| 104 | rs3781885 | T | C | 0.312544 | -0.01468 | 0.002654 | 3.20E-08 | 30.58364 |
| 105 | rs7938342 | A | T | 0.583824 | 0.025458 | 0.0025 | 2.41E-24 | 103.6719 |
| 106 | rs7123754 | C | T | 0.327459 | -0.018 | 0.002622 | 6.65E-12 | 47.13315 |
| 107 | rs2052691 | A | G | 0.287854 | 0.018941 | 0.002717 | 3.16E-12 | 48.59196 |
| 108 | rs10769253 | A | G | 0.176767 | -0.02394 | 0.003231 | 1.27E-13 | 54.90097 |
| 109 | rs4930676 | T | C | 0.10783 | -0.02433 | 0.003963 | 8.39E-10 | 37.6687 |
| 110 | rs11214436 | T | G | 0.382494 | -0.0155 | 0.002538 | 1.03E-09 | 37.26251 |
| 111 | rs3184504 | C | T | 0.517816 | -0.02148 | 0.002461 | 2.54E-18 | 76.23019 |
| 112 | rs4767332 | A | C | 0.581181 | 0.015379 | 0.002499 | 7.58E-10 | 37.86861 |
| 113 | rs60691990 | C | T | 0.342781 | -0.01968 | 0.002604 | 4.15E-14 | 57.10341 |
| 114 | rs4759062 | T | C | 0.298453 | -0.01527 | 0.002697 | 1.49E-08 | 32.06894 |
| 115 | rs2004283 | G | T | 0.578123 | -0.01367 | 0.00249 | 3.98E-08 | 30.15913 |
| 116 | rs11170386 | T | C | 0.2876 | -0.01691 | 0.002723 | 5.26E-10 | 38.57911 |
| 117 | rs73437338 | C | T | 0.166798 | -0.03765 | 0.003308 | 5.21E-30 | 129.5514 |
| 118 | rs35443 | C | G | 0.382529 | -0.01831 | 0.002525 | 4.06E-13 | 52.61957 |
| 119 | rs138110118 | G | T | 0.114477 | -0.0239 | 0.003908 | 9.61E-10 | 37.40401 |
| 120 | rs11616710 | T | C | 0.100975 | 0.026193 | 0.004324 | 1.39E-09 | 36.68801 |
| 121 | rs79621605 | C | T | 0.03444 | -0.03739 | 0.006801 | 3.85E-08 | 30.22398 |
| 122 | rs75989961 | G | T | 0.081642 | 0.024792 | 0.004493 | 3.44E-08 | 30.44334 |
| 123 | rs2240980 | G | C | 0.293396 | 0.015002 | 0.0027 | 2.74E-08 | 30.88254 |
| 124 | rs11629120 | C | T | 0.413676 | 0.013985 | 0.002508 | 2.46E-08 | 31.09025 |
| 125 | rs365990 | G | A | 0.369482 | -0.01665 | 0.002544 | 6.06E-11 | 42.80307 |
| 126 | rs9888615 | C | T | 0.710967 | 0.016954 | 0.002723 | 4.82E-10 | 38.75182 |
| 127 | rs11070245 | G | T | 0.530346 | 0.016523 | 0.002471 | 2.28E-11 | 44.71918 |
| 128 | rs4932373 | C | A | 0.326297 | 0.028522 | 0.002621 | 1.44E-27 | 118.3903 |
| 129 | rs74826317 | G | A | 0.028 | -0.05086 | 0.007431 | 7.70E-12 | 46.84423 |
| 130 | rs2627316 | G | A | 0.470869 | 0.019349 | 0.002465 | 4.20E-15 | 61.61027 |
| 131 | rs691830 | G | A | 0.476818 | 0.016941 | 0.002463 | 6.06E-12 | 47.31327 |
| 132 | rs2472299 | G | A | 0.727931 | -0.02167 | 0.002763 | 4.48E-15 | 61.48381 |
| 133 | rs3211995 | A | G | 0.158791 | -0.01992 | 0.003378 | 3.67E-09 | 34.79182 |
| 134 | rs11641308 | C | T | 0.653226 | 0.018085 | 0.002615 | 4.68E-12 | 47.82019 |
| 135 | rs2379829 | C | G | 0.732864 | -0.01678 | 0.002777 | 1.51E-09 | 36.525 |
| 136 | rs2303083 | A | G | 0.194525 | -0.02418 | 0.003111 | 7.86E-15 | 60.37694 |
| 137 | rs4843748 | A | G | 0.6687 | -0.01549 | 0.002619 | 3.35E-09 | 34.97037 |
| 138 | rs7200432 | A | G | 0.299598 | -0.01907 | 0.002693 | 1.42E-12 | 50.162 |
| 139 | rs77870048 | T | C | 0.05321 | 0.054451 | 0.005499 | 4.14E-23 | 98.03606 |
| 140 | rs4480845 | C | T | 0.639509 | -0.01994 | 0.002578 | 1.06E-14 | 59.78706 |
| 141 | rs35224044 | T | C | 0.584644 | 0.014976 | 0.002497 | 2.00E-09 | 35.97165 |
| 142 | rs55938136 | G | A | 0.226206 | -0.01693 | 0.002934 | 7.99E-09 | 33.27923 |
| 143 | rs1436138 | G | A | 0.358412 | -0.01566 | 0.002574 | 1.18E-09 | 37.00892 |
| 144 | rs11874 | A | G | 0.136912 | 0.026548 | 0.003575 | 1.12E-13 | 55.14136 |
| 145 | rs2288276 | C | G | 0.89739 | 0.02404 | 0.004059 | 3.16E-09 | 35.08486 |
| 146 | rs2301597 | C | T | 0.576399 | -0.023 | 0.002494 | 2.85E-20 | 85.10439 |
| 147 | rs3826537 | G | A | 0.427854 | 0.014316 | 0.002485 | 8.38E-09 | 33.18572 |
| 148 | rs2306526 | T | C | 0.524665 | -0.0157 | 0.002462 | 1.81E-10 | 40.66908 |
| 149 | rs78744936 | A | G | 0.268915 | 0.016205 | 0.002794 | 6.63E-09 | 33.64083 |
| 150 | rs1000423 | T | C | 0.735404 | 0.017808 | 0.002788 | 1.70E-10 | 40.78999 |
| 151 | rs6504213 | C | T | 0.591769 | 0.01545 | 0.002507 | 7.20E-10 | 37.96882 |
| 152 | rs12967060 | T | C | 0.267484 | 0.016679 | 0.002789 | 2.22E-09 | 35.77229 |
| 153 | rs11874246 | T | C | 0.293884 | 0.016884 | 0.0027 | 4.03E-10 | 39.09931 |
| 154 | rs10048404 | T | C | 0.366917 | -0.01493 | 0.002547 | 4.62E-09 | 34.34537 |
| 155 | rs144356415 | G | A | 0.0398 | 0.036359 | 0.00629 | 7.48E-09 | 33.40884 |
| 156 | rs2017199 | A | G | 0.300988 | -0.01472 | 0.002696 | 4.72E-08 | 29.82867 |
| 157 | rs10853912 | T | C | 0.399386 | 0.015436 | 0.00252 | 9.05E-10 | 37.52154 |
| 158 | rs10409243 | T | C | 0.602151 | -0.01536 | 0.002544 | 1.58E-09 | 36.43208 |
| 159 | rs167479 | T | G | 0.470348 | -0.02702 | 0.002461 | 4.76E-28 | 120.5851 |
| 160 | rs73046792 | A | G | 0.165198 | -0.02436 | 0.003307 | 1.74E-13 | 54.28005 |
| 161 | rs6026742 | A | G | 0.117761 | 0.031569 | 0.003833 | 1.79E-16 | 67.82884 |
| 162 | rs6031400 | T | C | 0.160422 | 0.019302 | 0.003366 | 9.82E-09 | 32.87873 |
| 163 | rs78473917 | C | T | 0.148716 | -0.01901 | 0.003462 | 4.03E-08 | 30.13531 |
| 164 | rs8121509 | C | T | 0.452031 | -0.01567 | 0.002471 | 2.29E-10 | 40.20385 |
| 165 | rs2423514 | G | A | 0.462868 | -0.01711 | 0.002463 | 3.75E-12 | 48.25364 |
| 166 | rs1887320 | A | G | 0.476784 | 0.018943 | 0.002466 | 1.57E-14 | 59.01726 |
| 167 | rs137923903 | T | C | 0.013495 | -0.06265 | 0.010751 | 5.65E-09 | 33.95365 |

SNP: Single nucleotide polymorphisms; SBP: systolic blood pressure; EAF: Effect allele frequency; SE：Standard error. F-statistics= (Effect/SE)^2^

^a^ Effect size of each allele on genetically-determined SBP (mmHg per allele)

^b^ Standard error of the genetic association of each effect allele with genetically-determined SBP

## Table S7. Characteristics of SNPs used as instrumental variables for genetically-determined TG.

|  | SNP | Effect allele | Other allele | EAF | Z_k_^a^ | σZ_k_^b^ | *P*-value | F-statistics |
| --- | --- | --- | --- | --- | --- | --- | --- | --- |
| 1 | rs1009590 | C | G | 0.914614 | 0.024721 | 0.004177 | 3.25E-09 | 35.02869 |
| 2 | rs114165349 | C | G | 0.023534 | 0.086252 | 0.007701 | 4.14E-29 | 125.4326 |
| 3 | rs213479 | T | G | 0.46666 | -0.01504 | 0.002336 | 1.22E-10 | 41.44261 |
| 4 | rs7529073 | C | T | 0.45914 | -0.01299 | 0.002329 | 2.42E-08 | 31.12612 |
| 5 | rs114052230 | T | C | 0.16619 | -0.0239 | 0.003218 | 1.10E-13 | 55.18288 |
| 6 | rs12088739 | G | A | 0.090568 | -0.02732 | 0.00404 | 1.36E-11 | 45.73209 |
| 7 | rs11122450 | G | T | 0.61312 | -0.04754 | 0.002383 | 1.60E-88 | 398.0883 |
| 8 | rs631106 | A | C | 0.35477 | -0.08064 | 0.002427 | 1.00E-200 | 1103.926 |
| 9 | rs10631642 | CTTT | C | 0.30981 | -0.01468 | 0.002564 | 1.03E-08 | 32.79333 |
| 10 | rs17311740 | T | C | 0.068033 | -0.02642 | 0.004631 | 1.17E-08 | 32.54069 |
| 11 | rs11206374 | A | G | 0.22477 | 0.025156 | 0.002781 | 1.49E-19 | 81.82409 |
| 12 | rs2114273 | C | T | 0.60228 | 0.017083 | 0.002366 | 5.23E-13 | 52.11811 |
| 13 | rs5005705 | A | C | 0.83382 | -0.0205 | 0.003117 | 4.85E-11 | 43.24212 |
| 14 | rs1044808 | C | G | 0.082428 | -0.02819 | 0.004206 | 2.06E-11 | 44.92126 |
| 15 | rs12749691 | T | A | 0.30337 | -0.02168 | 0.002556 | 2.22E-17 | 71.94639 |
| 16 | rs9425589 | A | G | 0.56622 | -0.01359 | 0.002341 | 6.44E-09 | 33.6976 |
| 17 | rs12119979 | G | C | 0.45854 | 0.020736 | 0.002328 | 5.26E-19 | 79.33851 |
| 18 | rs6547692 | A | G | 0.55637 | -0.08511 | 0.00233 | 1.00E-200 | 1334.298 |
| 19 | rs17326656 | T | G | 0.23895 | 0.016275 | 0.002733 | 2.60E-09 | 35.46715 |
| 20 | rs35602378 | CT | C | 0.42912 | -0.0199 | 0.002349 | 2.42E-17 | 71.76673 |
| 21 | rs77342729 | G | A | 0.030786 | 0.038828 | 0.006708 | 7.12E-09 | 33.50456 |
| 22 | rs10210970 | T | C | 0.12892 | 0.029922 | 0.00346 | 5.20E-18 | 74.80912 |
| 23 | rs6708784 | G | A | 0.49804 | -0.0131 | 0.002324 | 1.73E-08 | 31.77786 |
| 24 | rs676210 | A | G | 0.20412 | -0.07658 | 0.002874 | 3.02E-156 | 709.9116 |
| 25 | rs17496249 | G | A | 0.44515 | -0.01379 | 0.002344 | 4.03E-09 | 34.61477 |
| 26 | rs11688682 | C | G | 0.27053 | -0.0166 | 0.002701 | 7.94E-10 | 37.77629 |
| 27 | rs1128249 | T | G | 0.39297 | -0.03706 | 0.002373 | 5.70E-55 | 243.9357 |
| 28 | rs377352335 | AT | A | 0.3389 | -0.01413 | 0.002558 | 3.32E-08 | 30.51379 |
| 29 | rs35549624 | CA | C | 0.49467 | -0.01391 | 0.002449 | 1.33E-08 | 32.29002 |
| 30 | rs4675812 | A | G | 0.58767 | -0.01314 | 0.002355 | 2.43E-08 | 31.11373 |
| 31 | rs1473886 | T | G | 0.47846 | -0.01856 | 0.002323 | 1.36E-15 | 63.82381 |
| 32 | rs1275519 | C | T | 0.930255 | 0.065466 | 0.00455 | 6.20E-47 | 207.0547 |
| 33 | rs11442987 | TA | T | 0.55436 | -0.01302 | 0.002344 | 2.81E-08 | 30.83734 |
| 34 | rs2723067 | G | A | 0.40962 | -0.01981 | 0.002359 | 4.57E-17 | 70.52469 |
| 35 | rs73948269 | G | A | 0.015046 | 0.057243 | 0.009551 | 2.06E-09 | 35.92164 |
| 36 | rs1420384 | T | G | 0.66745 | -0.01438 | 0.002465 | 5.40E-09 | 34.04277 |
| 37 | rs3731696 | G | A | 0.12156 | 0.020535 | 0.003546 | 6.96E-09 | 33.54551 |
| 38 | rs2943645 | T | C | 0.64638 | 0.040295 | 0.002424 | 5.15E-62 | 276.2902 |
| 39 | rs9844972 | C | G | 0.071312 | 0.04177 | 0.004587 | 8.50E-20 | 82.94041 |
| 40 | rs13066793 | G | A | 0.090236 | -0.02285 | 0.004049 | 1.66E-08 | 31.86348 |
| 41 | rs7631606 | G | T | 0.26794 | -0.01556 | 0.002644 | 3.96E-09 | 34.64161 |
| 42 | rs34389637 | CT | C | 0.065993 | -0.02908 | 0.004681 | 5.19E-10 | 38.60553 |
| 43 | rs6792725 | G | A | 0.69343 | -0.01609 | 0.002592 | 5.38E-10 | 38.54043 |
| 44 | rs9836434 | T | C | 0.24167 | 0.01498 | 0.00272 | 3.63E-08 | 30.33986 |
| 45 | rs17052058 | G | A | 0.18183 | -0.03187 | 0.003006 | 2.95E-26 | 112.3981 |
| 46 | rs1279840 | C | T | 0.75021 | 0.028006 | 0.002684 | 1.71E-25 | 108.9178 |
| 47 | rs79287178 | A | G | 0.031313 | 0.054891 | 0.006991 | 4.13E-15 | 61.64338 |
| 48 | rs3105363 | G | A | 0.22831 | 0.02364 | 0.002861 | 1.43E-16 | 68.26507 |
| 49 | rs4647214 | AT | A | 0.42499 | -0.01433 | 0.002433 | 3.85E-09 | 34.69999 |
| 50 | rs58284370 | A | G | 0.095358 | 0.026881 | 0.003959 | 1.12E-11 | 46.10434 |
| 51 | rs13108218 | G | A | 0.61828 | -0.03175 | 0.002408 | 1.10E-39 | 173.8385 |
| 52 | rs293435 | T | C | 0.28679 | 0.01785 | 0.002567 | 3.58E-12 | 48.34561 |
| 53 | rs1471251 | T | A | 0.39916 | 0.038485 | 0.002374 | 4.21E-59 | 262.9084 |
| 54 | rs10470884 | A | G | 0.23032 | -0.01625 | 0.002774 | 4.71E-09 | 34.30908 |
| 55 | rs3822072 | A | G | 0.44865 | 0.016627 | 0.002331 | 9.86E-13 | 50.87518 |
| 56 | rs71603401 | G | A | 0.13508 | 0.026641 | 0.003424 | 7.25E-15 | 60.53523 |
| 57 | rs73243877 | G | A | 0.16771 | 0.027698 | 0.003101 | 4.22E-19 | 79.77472 |
| 58 | rs11722924 | C | G | 0.53768 | 0.013532 | 0.002324 | 5.84E-09 | 33.89239 |
| 59 | rs12513202 | T | C | 0.60001 | 0.013606 | 0.002395 | 1.33E-08 | 32.28187 |
| 60 | rs6554198 | A | G | 0.59026 | -0.01327 | 0.002358 | 1.82E-08 | 31.67911 |
| 61 | rs35225200 | C | A | 0.082956 | 0.031323 | 0.004333 | 4.85E-13 | 52.26965 |
| 62 | rs2139980 | A | G | 0.36565 | -0.01488 | 0.00242 | 7.84E-10 | 37.80138 |
| 63 | rs4976033 | G | A | 0.40117 | 0.018832 | 0.002398 | 4.01E-15 | 61.69865 |
| 64 | rs1045241 | T | C | 0.27114 | -0.02232 | 0.002625 | 1.85E-17 | 72.30766 |
| 65 | rs154735 | A | G | 0.06345 | 0.028025 | 0.004793 | 5.01E-09 | 34.18964 |
| 66 | rs112424890 | T | C | 0.18048 | 0.018175 | 0.003024 | 1.86E-09 | 36.11834 |
| 67 | rs10040328 | A | C | 0.26183 | 0.015304 | 0.002638 | 6.62E-09 | 33.64563 |
| 68 | rs7735249 | G | C | 0.11275 | 0.026359 | 0.003688 | 8.82E-13 | 51.09404 |
| 69 | rs593979 | C | T | 0.40133 | -0.01954 | 0.002496 | 4.90E-15 | 61.30191 |
| 70 | rs34580448 | C | T | 0.040976 | -0.03674 | 0.00586 | 3.59E-10 | 39.32346 |
| 71 | rs6882076 | C | T | 0.63568 | 0.036111 | 0.002407 | 7.25E-51 | 225.0935 |
| 72 | rs11429307 | GT | G | 0.1908 | 0.046725 | 0.00298 | 2.20E-55 | 245.8312 |
| 73 | rs142047875 | T | A | 0.57481 | -0.01339 | 0.002372 | 1.64E-08 | 31.87913 |
| 74 | rs1644005 | C | T | 0.36191 | -0.01437 | 0.002413 | 2.64E-09 | 35.43436 |
| 75 | rs72801474 | A | G | 0.092722 | -0.03138 | 0.004002 | 4.48E-15 | 61.48391 |
| 76 | rs9264277 | C | T | 0.63166 | 0.014514 | 0.002414 | 1.84E-09 | 36.1403 |
| 77 | rs28359800 | C | CAA | 0.28494 | 0.029511 | 0.00257 | 1.62E-30 | 131.8668 |
| 78 | rs998584 | A | C | 0.48163 | 0.03985 | 0.002326 | 9.14E-66 | 293.4944 |
| 79 | rs6916318 | T | A | 0.53269 | 0.02615 | 0.002329 | 3.01E-29 | 126.0679 |
| 80 | rs4710938 | G | A | 0.46191 | -0.0144 | 0.002328 | 6.15E-10 | 38.27642 |
| 81 | rs28752924 | C | T | 0.44037 | 0.031652 | 0.002416 | 3.19E-39 | 171.7071 |
| 82 | rs10872003 | A | T | 0.45283 | -0.01308 | 0.002337 | 2.17E-08 | 31.3324 |
| 83 | rs73025562 | A | G | 0.24549 | 0.015806 | 0.002705 | 5.12E-09 | 34.14613 |
| 84 | rs77009508 | G | A | 0.075135 | 0.045189 | 0.004407 | 1.13E-24 | 105.1618 |
| 85 | rs28383314 | C | T | 0.62442 | 0.041134 | 0.002393 | 3.42E-66 | 295.4721 |
| 86 | rs729761 | G | T | 0.71129 | 0.017988 | 0.002583 | 3.31E-12 | 48.49723 |
| 87 | rs1064173 | A | G | 0.28694 | -0.02486 | 0.002685 | 2.08E-20 | 85.72137 |
| 88 | rs707931 | G | A | 0.059246 | 0.032373 | 0.004915 | 4.50E-11 | 43.38646 |
| 89 | rs11752394 | G | C | 0.76426 | 0.017287 | 0.002735 | 2.63E-10 | 39.93904 |
| 90 | rs199607859 | T | G | 0.59411 | -0.02937 | 0.002368 | 2.53E-35 | 153.8597 |
| 91 | rs186696265 | T | C | 0.014913 | -0.10235 | 0.009619 | 1.95E-26 | 113.2299 |
| 92 | rs1835346 | G | A | 0.024081 | -0.04273 | 0.007611 | 1.98E-08 | 31.51713 |
| 93 | rs13234131 | G | A | 0.12909 | -0.12951 | 0.003448 | 1.00E-200 | 1410.494 |
| 94 | rs38205 | C | A | 0.62188 | -0.01486 | 0.002411 | 7.09E-10 | 37.99675 |
| 95 | rs4722551 | C | T | 0.15861 | -0.03714 | 0.00318 | 1.68E-31 | 136.3643 |
| 96 | rs2699805 | A | G | 0.40171 | -0.01851 | 0.002381 | 7.68E-15 | 60.42054 |
| 97 | rs2070971 | T | G | 0.13726 | 0.024664 | 0.00338 | 2.96E-13 | 53.24051 |
| 98 | rs2971669 | T | C | 0.21755 | 0.016266 | 0.002813 | 7.38E-09 | 33.43184 |
| 99 | rs4731701 | T | C | 0.49576 | -0.03368 | 0.002324 | 1.43E-47 | 209.9837 |
| 100 | rs112206063 | C | T | 0.20653 | 0.018934 | 0.00295 | 1.38E-10 | 41.19464 |
| 101 | rs852392 | A | G | 0.21724 | 0.01607 | 0.002811 | 1.08E-08 | 32.68443 |
| 102 | rs41749 | A | C | 0.452 | -0.015 | 0.002338 | 1.42E-10 | 41.14174 |
| 103 | rs4410790 | C | T | 0.63426 | 0.016122 | 0.00241 | 2.26E-11 | 44.73625 |
| 104 | rs17138358 | C | G | 0.39619 | 0.018621 | 0.002374 | 4.38E-15 | 61.52918 |
| 105 | rs917195 | T | C | 0.23078 | -0.01569 | 0.002781 | 1.69E-08 | 31.82346 |
| 106 | rs72555385 | G | A | 0.048707 | 0.066648 | 0.005405 | 6.24E-35 | 152.0656 |
| 107 | rs9692598 | G | A | 0.50737 | -0.01368 | 0.002322 | 3.82E-09 | 34.71335 |
| 108 | rs13273454 | T | C | 0.46899 | -0.05507 | 0.002327 | 1.11E-123 | 559.9193 |
| 109 | rs7826687 | G | C | 0.28447 | 0.032527 | 0.00257 | 1.05E-36 | 160.1974 |
| 110 | rs4841580 | C | T | 0.43423 | -0.02396 | 0.002347 | 1.87E-24 | 104.1668 |
| 111 | rs2081687 | C | T | 0.66344 | -0.02602 | 0.002453 | 2.75E-26 | 112.5373 |
| 112 | rs35859536 | T | C | 0.31321 | -0.01573 | 0.002506 | 3.48E-10 | 39.39118 |
| 113 | rs4646246 | G | A | 0.18492 | 0.030376 | 0.002987 | 2.73E-24 | 103.4236 |
| 114 | rs59347135 | G | C | 0.045638 | 0.074638 | 0.005702 | 3.81E-39 | 171.3607 |
| 115 | rs2954017 | C | T | 0.52034 | -0.08935 | 0.002321 | 1.00E-200 | 1482.54 |
| 116 | rs12541912 | C | G | 0.29104 | -0.09644 | 0.002547 | 1.00E-200 | 1433.356 |
| 117 | rs13269725 | G | A | 0.079506 | 0.03532 | 0.004292 | 1.88E-16 | 67.73341 |
| 118 | rs10957299 | G | T | 0.43483 | -0.01303 | 0.002342 | 2.66E-08 | 30.9428 |
| 119 | rs13275656 | C | T | 0.57013 | 0.013539 | 0.002478 | 4.68E-08 | 29.84939 |
| 120 | rs34081699 | A | G | 0.34124 | -0.0141 | 0.002491 | 1.54E-08 | 32.00944 |
| 121 | rs57994353 | C | T | 0.29973 | 0.014533 | 0.002529 | 9.18E-09 | 33.01228 |
| 122 | rs10797119 | C | T | 0.53651 | 0.017236 | 0.002338 | 1.68E-13 | 54.3527 |
| 123 | rs550057 | T | C | 0.25477 | -0.01938 | 0.002668 | 3.77E-13 | 52.76527 |
| 124 | rs296884 | T | G | 0.25504 | -0.02353 | 0.002666 | 1.07E-18 | 77.92322 |
| 125 | rs1853413 | G | C | 0.34534 | -0.01411 | 0.002446 | 8.14E-09 | 33.24503 |
| 126 | rs1924485 | T | G | 0.13227 | -0.0205 | 0.003431 | 2.28E-09 | 35.7207 |
| 127 | rs12686780 | T | C | 0.1742 | 0.016979 | 0.003061 | 2.90E-08 | 30.7719 |
| 128 | rs1800978 | G | C | 0.12415 | -0.02552 | 0.003533 | 5.11E-13 | 52.16753 |
| 129 | rs76669111 | T | G | 0.15652 | -0.02411 | 0.003198 | 4.75E-14 | 56.8366 |
| 130 | rs7896783 | A | G | 0.47239 | -0.03407 | 0.002324 | 1.21E-48 | 214.905 |
| 131 | rs11000468 | T | C | 0.25412 | -0.01696 | 0.002708 | 3.76E-10 | 39.23292 |
| 132 | rs2068888 | A | G | 0.44892 | -0.03081 | 0.002329 | 6.14E-40 | 174.9879 |
| 133 | rs2773469 | G | A | 0.73563 | -0.02027 | 0.002638 | 1.57E-14 | 59.0165 |
| 134 | rs1133400 | G | A | 0.22221 | 0.016796 | 0.002787 | 1.68E-09 | 36.31151 |
| 135 | rs11187019 | G | A | 0.54999 | -0.01302 | 0.00234 | 2.67E-08 | 30.93811 |
| 136 | rs74563318 | A | C | 0.035971 | -0.05058 | 0.006321 | 1.24E-15 | 64.01367 |
| 137 | rs34875072 | T | G | 0.038731 | -0.03742 | 0.006004 | 4.60E-10 | 38.84056 |
| 138 | rs113344423 | A | G | 0.061078 | 0.042829 | 0.004969 | 6.75E-18 | 74.29428 |
| 139 | rs10883026 | T | C | 0.52035 | -0.01507 | 0.002338 | 1.16E-10 | 41.53736 |
| 140 | rs17699425 | A | G | 0.058141 | -0.028 | 0.004949 | 1.54E-08 | 32.00449 |
| 141 | rs34931109 | TA | T | 0.23887 | -0.01763 | 0.002794 | 2.80E-10 | 39.8138 |
| 142 | rs45487899 | T | C | 0.048032 | -0.03648 | 0.005453 | 2.24E-11 | 44.75061 |
| 143 | rs57232565 | T | C | 0.055365 | 0.19444 | 0.005073 | 1.00E-200 | 1469.182 |
| 144 | rs6486122 | T | C | 0.69111 | 0.019909 | 0.002508 | 2.04E-15 | 63.03509 |
| 145 | rs11600815 | A | G | 0.052967 | -0.03652 | 0.005303 | 5.73E-12 | 47.42522 |
| 146 | rs10750766 | A | C | 0.71086 | 0.021577 | 0.00256 | 3.55E-17 | 71.02323 |
| 147 | rs7952521 | A | G | 0.10547 | -0.02914 | 0.003902 | 8.09E-14 | 55.78771 |
| 148 | rs7123454 | A | C | 0.88225 | -0.12778 | 0.0036 | 1.00E-200 | 1260.066 |
| 149 | rs11030107 | G | A | 0.26437 | 0.016587 | 0.00263 | 2.87E-10 | 39.76419 |
| 150 | rs12281051 | C | A | 0.013535 | 0.070032 | 0.010053 | 3.26E-12 | 48.52904 |
| 151 | rs12294913 | G | C | 0.048646 | 0.055264 | 0.005557 | 2.69E-23 | 98.89104 |
| 152 | rs7117238 | A | G | 0.15846 | -0.01894 | 0.003174 | 2.41E-09 | 35.61394 |
| 153 | rs10838681 | A | G | 0.2683 | -0.02629 | 0.002614 | 8.65E-24 | 101.1355 |
| 154 | rs174574 | C | A | 0.64696 | -0.0512 | 0.002429 | 1.47E-98 | 444.2703 |
| 155 | rs4930352 | T | G | 0.4955 | -0.01377 | 0.002361 | 5.48E-09 | 34.01378 |
| 156 | rs79291519 | T | C | 0.05259 | -0.06463 | 0.005209 | 2.46E-35 | 153.9145 |
| 157 | rs187929675 | T | C | 0.013492 | -0.17449 | 0.010185 | 9.13E-66 | 293.5074 |
| 158 | rs1790099 | T | C | 0.70835 | 0.016651 | 0.002564 | 8.40E-11 | 42.16411 |
| 159 | rs11057837 | T | C | 0.10263 | 0.021831 | 0.003838 | 1.28E-08 | 32.35807 |
| 160 | rs199795230 | T | C | 0.15826 | 0.026592 | 0.003184 | 6.77E-17 | 69.74749 |
| 161 | rs4761234 | C | T | 0.48647 | -0.01633 | 0.002328 | 2.29E-12 | 49.21853 |
| 162 | rs12424054 | A | G | 0.23414 | 0.019019 | 0.002739 | 3.83E-12 | 48.21249 |
| 163 | rs4930724 | C | T | 0.32972 | -0.02658 | 0.002467 | 4.51E-27 | 116.1221 |
| 164 | rs76895963 | G | T | 0.021076 | -0.09073 | 0.008901 | 2.14E-24 | 103.909 |
| 165 | rs10772947 | G | A | 0.529 | -0.01368 | 0.002324 | 4.02E-09 | 34.61548 |
| 166 | rs4760254 | C | G | 0.24187 | -0.02841 | 0.002705 | 8.18E-26 | 110.3723 |
| 167 | rs863750 | T | C | 0.60455 | 0.029472 | 0.002377 | 2.66E-35 | 153.7567 |
| 168 | rs7298844 | G | A | 0.20792 | 0.01571 | 0.002852 | 3.62E-08 | 30.34264 |
| 169 | rs11045171 | G | A | 0.1977 | -0.0286 | 0.002927 | 1.54E-22 | 95.42806 |
| 170 | rs2694913 | C | A | 0.64298 | 0.014079 | 0.002425 | 6.43E-09 | 33.70144 |
| 171 | rs1928496 | T | C | 0.74159 | 0.01667 | 0.002647 | 3.01E-10 | 39.66696 |
| 172 | rs2812208 | C | G | 0.021097 | -0.05154 | 0.008074 | 1.74E-10 | 40.74013 |
| 173 | rs12868517 | G | T | 0.24596 | -0.01482 | 0.002714 | 4.79E-08 | 29.80104 |
| 174 | rs1340819 | C | A | 0.347 | -0.01346 | 0.002439 | 3.42E-08 | 30.45447 |
| 175 | rs11274835 | CGAGTGTGGGAATCT | C | 0.17376 | -0.0285 | 0.003226 | 1.03E-18 | 78.00661 |
| 176 | rs6492721 | C | T | 0.68531 | -0.01482 | 0.002496 | 2.92E-09 | 35.23577 |
| 177 | rs7140110 | C | T | 0.29697 | 0.029279 | 0.002539 | 9.05E-31 | 133.0221 |
| 178 | rs12880341 | C | T | 0.15822 | 0.020424 | 0.003194 | 1.62E-10 | 40.87926 |
| 179 | rs61993685 | C | T | 0.077957 | -0.02388 | 0.004319 | 3.22E-08 | 30.57304 |
| 180 | rs139624990 | T | C | 0.009891 | 0.068628 | 0.012516 | 4.18E-08 | 30.06572 |
| 181 | rs11631625 | G | A | 0.25901 | 0.014743 | 0.00265 | 2.64E-08 | 30.95839 |
| 182 | rs7167078 | G | C | 0.31213 | -0.01537 | 0.002505 | 8.52E-10 | 37.6377 |
| 183 | rs139974673 | C | T | 0.024986 | 0.14437 | 0.007442 | 8.71E-84 | 376.3249 |
| 184 | rs422137 | A | G | 0.43734 | 0.026391 | 0.002352 | 3.21E-29 | 125.9354 |
| 185 | rs261342 | C | G | 0.78004 | -0.04704 | 0.002812 | 8.05E-63 | 279.9715 |
| 186 | rs2652812 | T | C | 0.76651 | -0.01825 | 0.002759 | 3.76E-11 | 43.73853 |
| 187 | rs10851698 | T | C | 0.26147 | 0.016157 | 0.002648 | 1.06E-09 | 37.21811 |
| 188 | rs3826043 | T | C | 0.42911 | -0.01364 | 0.002361 | 7.52E-09 | 33.40012 |
| 189 | rs8025505 | T | C | 0.2558 | 0.022162 | 0.002668 | 1.00E-16 | 68.97886 |
| 190 | rs34967613 | CTT | C | 0.46818 | 0.015088 | 0.002338 | 1.09E-10 | 41.66035 |
| 191 | rs12928099 | A | C | 0.29628 | -0.03073 | 0.002544 | 1.33E-33 | 145.9788 |
| 192 | rs2925979 | C | T | 0.69891 | -0.03151 | 0.002528 | 1.14E-35 | 155.44 |
| 193 | rs12446515 | T | C | 0.32444 | -0.03551 | 0.002486 | 2.88E-46 | 204.011 |
| 194 | rs1684608 | A | C | 0.19225 | 0.01824 | 0.002945 | 5.90E-10 | 38.35483 |
| 195 | rs4843754 | G | A | 0.49776 | 0.014204 | 0.002326 | 1.02E-09 | 37.278 |
| 196 | rs34682685 | A | G | 0.10519 | 0.033067 | 0.003795 | 2.97E-18 | 75.91772 |
| 197 | rs1549293 | T | C | 0.35553 | -0.01572 | 0.002426 | 9.22E-11 | 41.98279 |
| 198 | rs8051062 | C | T | 0.57547 | -0.01977 | 0.002396 | 1.56E-16 | 68.09901 |
| 199 | rs74456742 | A | G | 0.037384 | -0.04661 | 0.006311 | 1.52E-13 | 54.55177 |
| 200 | rs1801689 | C | A | 0.029886 | -0.06379 | 0.006811 | 7.60E-21 | 87.71676 |
| 201 | rs60856912 | T | G | 0.16247 | 0.025468 | 0.003162 | 7.96E-16 | 64.88561 |
| 202 | rs4969179 | G | T | 0.60395 | -0.01847 | 0.002374 | 7.23E-15 | 60.54408 |
| 203 | rs72836561 | T | C | 0.031831 | 0.14118 | 0.006603 | 2.37E-101 | 457.1415 |
| 204 | rs115271198 | T | C | 0.069597 | -0.0276 | 0.00457 | 1.54E-09 | 36.48483 |
| 205 | rs591939 | G | A | 0.25019 | 0.020518 | 0.002681 | 1.98E-14 | 58.56142 |
| 206 | rs11078597 | C | T | 0.18634 | 0.020328 | 0.002976 | 8.39E-12 | 46.6734 |
| 207 | rs56030759 | C | T | 0.060888 | 0.040682 | 0.00486 | 5.76E-17 | 70.06422 |
| 208 | rs1292065 | G | C | 0.7105 | -0.01624 | 0.002555 | 2.08E-10 | 40.38906 |
| 209 | rs4789182 | A | G | 0.73353 | 0.015239 | 0.002622 | 6.15E-09 | 33.78679 |
| 210 | rs11664106 | T | A | 0.37354 | -0.01479 | 0.002456 | 1.70E-09 | 36.2879 |
| 211 | rs6506033 | T | C | 0.071494 | -0.02948 | 0.004505 | 6.01E-11 | 42.81614 |
| 212 | rs867939 | A | G | 0.57662 | -0.01551 | 0.00236 | 4.97E-11 | 43.19354 |
| 213 | rs68033110 | A | G | 0.24701 | 0.017831 | 0.002731 | 6.63E-11 | 42.6324 |
| 214 | rs2510344 | C | T | 0.49298 | -0.01706 | 0.002323 | 2.06E-13 | 53.9526 |
| 215 | rs116843064 | A | G | 0.019519 | -0.2279 | 0.008364 | 2.67E-163 | 742.4209 |
| 216 | rs62117489 | A | C | 0.054659 | -0.04574 | 0.005116 | 3.88E-19 | 79.94057 |
| 217 | rs58542926 | T | C | 0.075445 | -0.10552 | 0.004391 | 1.73E-127 | 577.4087 |
| 218 | rs188247550 | T | C | 0.013291 | -0.13663 | 0.010583 | 4.02E-38 | 166.6766 |
| 219 | rs62118471 | C | T | 0.027197 | 0.048633 | 0.007581 | 1.41E-10 | 41.15589 |
| 220 | rs12151142 | C | T | 0.44222 | 0.019143 | 0.002342 | 3.00E-16 | 66.81073 |
| 221 | rs10405944 | C | T | 0.48383 | -0.01303 | 0.002358 | 3.32E-08 | 30.50921 |
| 222 | rs739320 | C | T | 0.60407 | -0.02129 | 0.002426 | 1.75E-18 | 76.96515 |
| 223 | rs62112763 | G | C | 0.44101 | 0.020615 | 0.002346 | 1.55E-18 | 77.20343 |
| 224 | rs62102718 | T | A | 0.28613 | 0.020836 | 0.002572 | 5.50E-16 | 65.61233 |
| 225 | rs1688043 | T | C | 0.933843 | 0.028631 | 0.004665 | 8.38E-10 | 37.6726 |
| 226 | rs34690548 | CAAA | C | 0.10754 | 0.023253 | 0.004034 | 8.18E-09 | 33.23486 |
| 227 | rs483082 | T | G | 0.23737 | 0.089363 | 0.002726 | 1.00E-200 | 1074.8 |
| 228 | rs5112 | G | C | 0.53202 | 0.068337 | 0.002486 | 3.29E-166 | 755.8131 |
| 229 | rs149142833 | T | C | 0.1562 | 0.01933 | 0.003222 | 1.99E-09 | 35.98585 |
| 230 | rs6066138 | A | G | 0.28398 | -0.01977 | 0.002577 | 1.69E-14 | 58.87204 |
| 231 | rs6093446 | A | G | 0.2869 | 0.014235 | 0.002566 | 2.91E-08 | 30.76806 |
| 232 | rs7274718 | A | G | 0.59909 | 0.015787 | 0.002364 | 2.43E-11 | 44.59309 |
| 233 | rs8126001 | T | C | 0.48973 | -0.01806 | 0.002327 | 8.43E-15 | 60.23345 |
| 234 | rs6073958 | C | T | 0.19849 | 0.055687 | 0.002908 | 1.00E-81 | 366.8328 |
| 235 | rs12480662 | T | C | 0.25075 | -0.01547 | 0.002687 | 8.49E-09 | 33.16241 |
| 236 | rs5755799 | G | C | 0.45279 | 0.013109 | 0.002333 | 1.92E-08 | 31.57796 |
| 237 | rs2267373 | T | C | 0.58124 | 0.022467 | 0.002352 | 1.29E-21 | 91.22308 |
| 238 | rs2071887 | A | T | 0.3433 | 0.016832 | 0.002444 | 5.70E-12 | 47.43562 |
| 239 | rs11705483 | A | C | 0.11078 | 0.023944 | 0.00369 | 8.69E-11 | 42.09655 |
| 240 | rs5965373 | G | T | 0.85712 | -0.01765 | 0.002742 | 1.22E-10 | 41.43513 |

SNP: Single nucleotide polymorphisms; TG: triglycerides; EAF: Effect allele frequency; SE：Standard error. F-statistics= (Effect/SE)^2^

^a^ Effect size of each allele on genetically-determined TG (mmol/l per allele)

^b^ Standard error of the genetic association of each effect allele with genetically-determined TG

## Table S8. Characteristics of SNPs used as instrumental variables for genetically-determined alcohol intake frequency.

|  | SNP | Effect allele | Other allele | EAF | Z_k_^a^ | σZ_k_^b^ | *P*-value | F-statistics | |
| --- | --- | --- | --- | --- | --- | --- | --- | --- | --- |
| 1 | rs780569 | A | T | 0.70882 | 0.019803 | 0.003365 | 4.00E-09 | 34.64366 |  |
| 2 | rs4503294 | T | C | 0.565333 | 0.018148 | 0.00307 | 3.40E-09 | 34.93197 |  |
| 3 | rs28787109 | A | G | 0.40423 | 0.017811 | 0.003085 | 7.70E-09 | 33.33968 |  |
| 4 | rs2244598 | C | T | 0.605114 | -0.01838 | 0.003119 | 3.80E-09 | 34.71732 |  |
| 5 | rs4417025 | A | G | 0.361153 | -0.01884 | 0.003165 | 2.70E-09 | 35.42205 |  |
| 6 | rs7514579 | C | A | 0.232457 | 0.019667 | 0.003598 | 4.60E-08 | 29.88077 |  |
| 7 | rs2717063 | A | C | 0.585731 | -0.02037 | 0.003085 | 4.00E-11 | 43.61239 |  |
| 8 | rs6727281 | T | C | 0.184023 | -0.02432 | 0.00392 | 5.50E-10 | 38.50403 |  |
| 9 | rs780094 | C | T | 0.615206 | -0.05099 | 0.003105 | 1.30E-60 | 269.7086 |  |
| 10 | rs13390019 | C | T | 0.134041 | 0.029612 | 0.004492 | 4.30E-11 | 43.45893 |  |
| 11 | rs10188314 | T | C | 0.470852 | -0.01979 | 0.003036 | 7.20E-11 | 42.47542 |  |
| 12 | rs4241258 | T | C | 0.13763 | 0.025064 | 0.004403 | 1.30E-08 | 32.39964 |  |
| 13 | rs72769229 | T | A | 0.154942 | -0.02314 | 0.004192 | 3.40E-08 | 30.46487 |  |
| 14 | rs17662759 | C | T | 0.089115 | 0.030135 | 0.00546 | 3.40E-08 | 30.45805 |  |
| 15 | rs1991083 | T | C | 0.679886 | -0.02239 | 0.003258 | 6.30E-12 | 47.23548 |  |
| 16 | rs473098 | T | C | 0.557689 | -0.02174 | 0.003043 | 9.10E-13 | 51.02825 |  |
| 17 | rs9829192 | T | G | 0.435133 | 0.016932 | 0.00305 | 2.80E-08 | 30.81387 |  |
| 18 | rs76082653 | T | C | 0.054327 | 0.046427 | 0.006687 | 3.80E-12 | 48.20719 |  |
| 19 | rs262240 | T | C | 0.468553 | -0.01721 | 0.003035 | 1.40E-08 | 32.14708 |  |
| 20 | rs9814516 | T | G | 0.237423 | -0.02511 | 0.003556 | 1.60E-12 | 49.87186 |  |
| 21 | rs7610856 | A | C | 0.429053 | -0.02386 | 0.00307 | 7.70E-15 | 60.41538 |  |
| 22 | rs1515591 | G | T | 0.383186 | 0.01823 | 0.003116 | 4.90E-09 | 34.2212 |  |
| 23 | rs1228589 | A | G | 0.246133 | 0.02107 | 0.003528 | 2.30E-09 | 35.66589 |  |
| 24 | rs28622224 | T | C | 0.280364 | -0.01862 | 0.003368 | 3.20E-08 | 30.55917 |  |
| 25 | rs13135092 | G | A | 0.083483 | 0.043834 | 0.005499 | 1.60E-15 | 63.54509 |  |
| 26 | rs11940694 | G | A | 0.604193 | -0.04371 | 0.003116 | 1.00E-44 | 196.7964 |  |
| 27 | rs362307 | T | C | 0.074582 | 0.043305 | 0.005802 | 8.40E-14 | 55.70397 |  |
| 28 | rs1229984 | C | T | 0.97277 | -0.26171 | 0.009185 | 1.40E-178 | 811.8568 |  |
| 29 | rs13102973 | C | T | 0.61881 | -0.01941 | 0.003119 | 4.90E-10 | 38.72261 |  |
| 30 | rs62339673 | A | C | 0.626705 | 0.018294 | 0.003154 | 6.60E-09 | 33.64277 |  |
| 31 | rs34811474 | A | G | 0.230728 | -0.02018 | 0.003593 | 1.90E-08 | 31.54674 |  |
| 32 | rs2159935 | A | G | 0.490369 | -0.01857 | 0.003026 | 8.30E-10 | 37.68201 |  |
| 33 | rs62305780 | G | C | 0.102253 | -0.04852 | 0.005066 | 9.90E-22 | 91.74144 |  |
| 34 | rs13178443 | T | C | 0.276349 | -0.01865 | 0.00339 | 3.80E-08 | 30.27446 |  |
| 35 | rs11750777 | A | G | 0.209454 | -0.02049 | 0.003726 | 3.80E-08 | 30.24789 |  |
| 36 | rs4916723 | C | A | 0.420617 | 0.023948 | 0.0031 | 1.10E-14 | 59.69649 |  |
| 37 | rs461599 | C | A | 0.462259 | -0.01919 | 0.00304 | 2.70E-10 | 39.8487 |  |
| 38 | rs56194430 | T | C | 0.16931 | 0.02254 | 0.004071 | 3.10E-08 | 30.64889 |  |
| 39 | rs9403297 | A | G | 0.372967 | 0.018823 | 0.00313 | 1.80E-09 | 36.15803 |  |
| 40 | rs9349379 | G | A | 0.405493 | -0.01935 | 0.003082 | 3.50E-10 | 39.39602 |  |
| 41 | rs12153855 | C | T | 0.10497 | 0.029444 | 0.004935 | 2.40E-09 | 35.59858 |  |
| 42 | rs9372625 | A | G | 0.381706 | -0.02556 | 0.003125 | 2.90E-16 | 66.90245 |  |
| 43 | rs62466318 | T | C | 0.202827 | -0.02549 | 0.003774 | 1.40E-11 | 45.61993 |  |
| 44 | rs2622167 | A | G | 0.428653 | -0.01912 | 0.003067 | 4.60E-10 | 38.83564 |  |
| 45 | rs73050128 | A | C | 0.164488 | -0.026 | 0.004091 | 2.10E-10 | 40.40858 |  |
| 46 | rs6943160 | C | T | 0.208646 | 0.020627 | 0.003728 | 3.10E-08 | 30.61829 |  |
| 47 | rs4726481 | T | G | 0.400576 | 0.021761 | 0.003102 | 2.30E-12 | 49.21797 |  |
| 48 | rs9648478 | A | G | 0.510245 | 0.01686 | 0.003029 | 2.60E-08 | 30.98545 |  |
| 49 | rs2160935 | T | C | 0.604293 | -0.01872 | 0.003091 | 1.40E-09 | 36.66681 |  |
| 50 | rs34440851 | T | C | 0.157151 | -0.02268 | 0.004151 | 4.60E-08 | 29.8667 |  |
| 51 | rs11787216 | T | C | 0.369127 | 0.024416 | 0.003201 | 2.40E-14 | 58.18948 |  |
| 52 | rs2977454 | G | C | 0.124072 | -0.02592 | 0.004599 | 1.70E-08 | 31.77647 |  |
| 53 | rs74679146 | C | T | 0.074515 | -0.03207 | 0.005758 | 2.50E-08 | 31.03232 |  |
| 54 | rs489062 | A | G | 0.437454 | 0.01665 | 0.003053 | 4.90E-08 | 29.74336 |  |
| 55 | rs34473884 | A | G | 0.24819 | -0.02036 | 0.003503 | 6.20E-09 | 33.77732 |  |
| 56 | rs61873510 | T | G | 0.32785 | 0.020374 | 0.003303 | 6.90E-10 | 38.04466 |  |
| 57 | rs4242715 | A | G | 0.680585 | -0.01865 | 0.003248 | 9.30E-09 | 32.97942 |  |
| 58 | rs10792669 | G | A | 0.505254 | 0.017432 | 0.003041 | 9.90E-09 | 32.86787 |  |
| 59 | rs11223617 | A | G | 0.206155 | 0.025091 | 0.003754 | 2.30E-11 | 44.67705 |  |
| 60 | rs550942 | T | C | 0.823865 | 0.022401 | 0.003989 | 2.00E-08 | 31.53854 |  |
| 61 | rs11039429 | T | C | 0.454624 | -0.02356 | 0.003037 | 8.70E-15 | 60.16401 |  |
| 62 | rs1666658 | C | T | 0.392206 | 0.017967 | 0.003099 | 6.70E-09 | 33.62193 |  |
| 63 | rs12312693 | C | T | 0.451772 | -0.01768 | 0.00305 | 6.80E-09 | 33.60043 |  |
| 64 | rs7302200 | A | G | 0.339998 | -0.01842 | 0.003198 | 8.40E-09 | 33.17529 |  |
| 65 | rs28768122 | C | T | 0.759525 | 0.0207 | 0.003552 | 5.60E-09 | 33.96135 |  |
| 66 | rs7298932 | G | A | 0.147849 | -0.02372 | 0.004312 | 3.80E-08 | 30.26855 |  |
| 67 | rs58905411 | A | G | 0.410052 | -0.02663 | 0.003078 | 5.10E-18 | 74.85312 |  |
| 68 | rs1937522 | G | A | 0.528054 | 0.016898 | 0.003032 | 2.50E-08 | 31.05897 |  |
| 69 | rs7330939 | T | C | 0.720352 | -0.02133 | 0.003405 | 3.70E-10 | 39.25126 |  |
| 70 | rs2535911 | T | C | 0.354749 | -0.01885 | 0.003168 | 2.70E-09 | 35.38404 |  |
| 71 | rs186347 | T | G | 0.463343 | 0.017949 | 0.003051 | 4.00E-09 | 34.61576 |  |
| 72 | rs80292319 | C | T | 0.057704 | -0.03937 | 0.006496 | 1.40E-09 | 36.73866 |  |
| 73 | rs117799466 | C | G | 0.336989 | -0.01967 | 0.00332 | 3.10E-09 | 35.11025 |  |
| 74 | rs34631026 | T | C | 0.446061 | -0.01691 | 0.003048 | 2.90E-08 | 30.78293 |  |
| 75 | rs72787062 | A | G | 0.162767 | -0.02819 | 0.004103 | 6.40E-12 | 47.21512 |  |
| 76 | rs35105141 | T | C | 0.401541 | 0.026345 | 0.003088 | 1.40E-17 | 72.79065 |  |
| 77 | rs1421085 | C | T | 0.403447 | 0.019939 | 0.003085 | 1.00E-10 | 41.77906 |  |
| 78 | rs1104608 | C | G | 0.426338 | 0.017421 | 0.003088 | 1.70E-08 | 31.81733 |  |
| 79 | rs8043563 | C | G | 0.737192 | 0.023365 | 0.003471 | 1.70E-11 | 45.30798 |  |
| 80 | rs2411453 | G | T | 0.597353 | -0.03508 | 0.00309 | 7.30E-30 | 128.8473 |  |
| 81 | rs728538 | G | T | 0.168868 | 0.022875 | 0.004063 | 1.80E-08 | 31.70471 |  |
| 82 | rs9906502 | A | G | 0.176998 | 0.023788 | 0.003962 | 1.90E-09 | 36.05339 |  |
| 83 | rs8614 | A | C | 0.182509 | 0.024781 | 0.003925 | 2.70E-10 | 39.85313 |  |
| 84 | rs4968391 | T | G | 0.674892 | -0.01927 | 0.003227 | 2.30E-09 | 35.6679 |  |
| 85 | rs9912298 | C | A | 0.239585 | 0.020589 | 0.00359 | 9.70E-09 | 32.8939 |  |
| 86 | rs17690703 | T | C | 0.262687 | 0.025034 | 0.00343 | 2.90E-13 | 53.26305 |  |
| 87 | rs650558 | T | C | 0.247918 | 0.020736 | 0.003508 | 3.40E-09 | 34.9433 |  |
| 88 | rs1893659 | A | C | 0.459939 | -0.02933 | 0.003053 | 7.60E-22 | 92.26034 |  |
| 89 | rs5022348 | T | C | 0.40703 | 0.020264 | 0.00357 | 1.40E-08 | 32.21855 |  |
| 90 | rs2043677 | T | C | 0.145599 | 0.026113 | 0.004327 | 1.60E-09 | 36.41745 |  |
| 91 | rs9958320 | C | T | 0.153147 | 0.024855 | 0.004271 | 5.90E-09 | 33.86844 |  |
| 92 | rs62097995 | A | T | 0.423591 | 0.020002 | 0.003067 | 6.90E-11 | 42.53904 |  |
| 93 | rs2924321 | A | G | 0.539592 | -0.01951 | 0.00305 | 1.60E-10 | 40.92514 |  |
| 94 | rs4940926 | C | T | 0.735045 | -0.0191 | 0.003441 | 2.80E-08 | 30.8171 |  |
| 95 | rs838145 | A | G | 0.542982 | 0.021955 | 0.003055 | 6.70E-13 | 51.62942 |  |
| 96 | rs6030200 | A | G | 0.31415 | -0.01953 | 0.003271 | 2.40E-09 | 35.65047 |  |
| 97 | rs11700855 | G | A | 0.093465 | -0.0298 | 0.005233 | 1.20E-08 | 32.41892 |  |
| 98 | rs71651683 | T | C | 0.0142 | -0.07046 | 0.012791 | 3.60E-08 | 30.34519 |  |
| 99 | rs1894544 | C | G | 0.454379 | 0.017393 | 0.003046 | 1.10E-08 | 32.60072 |  |

SNP: Single nucleotide polymorphisms; EAF: Effect allele frequency; SE：Standard error. F-statistics= (Effect/SE)^2^

^a^ Effect size of each allele on genetically-determined alcohol intake frequency (times per week/ allele)

^b^ Standard error of the genetic association of each effect allele with genetically-determined alcohol intake frequency

## Table S9. Characteristics of SNPs used as instrumental variables for genetically-determined sleep duration.

|  | SNP | Effect allele | Other allele | EAF | Z_k_^a^ | σZ_k_^b^ | *P*-value | F-statistics |
| --- | --- | --- | --- | --- | --- | --- | --- | --- |
| 1 | rs915416 | G | C | 0.70906 | -0.01273 | 0.00176 | 4.80E-13 | 52.27667 |
| 2 | rs2186122 | T | A | 0.559911 | -0.01083 | 0.001621 | 2.30E-11 | 44.68473 |
| 3 | rs2279681 | G | C | 0.341785 | 0.009299 | 0.001685 | 3.40E-08 | 30.46195 |
| 4 | rs7517981 | C | T | 0.601498 | -0.00997 | 0.001634 | 1.10E-09 | 37.19776 |
| 5 | rs12567114 | A | G | 0.276368 | 0.012338 | 0.001794 | 6.10E-12 | 47.28244 |
| 6 | rs1463053 | A | G | 0.639694 | 0.00927 | 0.001661 | 2.40E-08 | 31.13223 |
| 7 | rs6681755 | A | G | 0.199783 | 0.011527 | 0.002005 | 9.00E-09 | 33.05427 |
| 8 | rs374153 | T | C | 0.842567 | -0.0131 | 0.002197 | 2.50E-09 | 35.54779 |
| 9 | rs2863957 | A | C | 0.220537 | 0.028904 | 0.001929 | 9.60E-51 | 224.4592 |
| 10 | rs1972712 | C | T | 0.249455 | 0.011796 | 0.001848 | 1.70E-10 | 40.76271 |
| 11 | rs72831782 | A | T | 0.269409 | -0.01018 | 0.001844 | 3.40E-08 | 30.48996 |
| 12 | rs2683630 | G | C | 0.629076 | 0.014951 | 0.001656 | 1.70E-19 | 81.55656 |
| 13 | rs75539574 | C | A | 0.085774 | 0.023665 | 0.002874 | 1.80E-16 | 67.78963 |
| 14 | rs35662245 | A | T | 0.338713 | 0.010157 | 0.001691 | 1.90E-09 | 36.08806 |
| 15 | rs6783516 | T | G | 0.583817 | -0.00984 | 0.001631 | 1.60E-09 | 36.37506 |
| 16 | rs76258078 | G | A | 0.049994 | -0.02169 | 0.003681 | 3.80E-09 | 34.70532 |
| 17 | rs113021516 | C | G | 0.335898 | 0.011481 | 0.001697 | 1.30E-11 | 45.75571 |
| 18 | rs17732997 | G | C | 0.429679 | -0.00884 | 0.001618 | 4.60E-08 | 29.85779 |
| 19 | rs9810474 | T | C | 0.232184 | -0.01115 | 0.001894 | 3.90E-09 | 34.68591 |
| 20 | rs7644809 | C | T | 0.576033 | -0.01015 | 0.001625 | 4.10E-10 | 39.04384 |
| 21 | rs13107325 | T | C | 0.074905 | -0.02427 | 0.003039 | 1.40E-15 | 63.76732 |
| 22 | rs2192528 | G | A | 0.522493 | -0.00981 | 0.001601 | 9.10E-10 | 37.50263 |
| 23 | rs2839753 | C | T | 0.265347 | -0.01064 | 0.001812 | 4.40E-09 | 34.44163 |
| 24 | rs7711696 | T | G | 0.304987 | -0.00987 | 0.001735 | 1.30E-08 | 32.33131 |
| 25 | rs12518468 | C | T | 0.328685 | -0.01064 | 0.001703 | 4.10E-10 | 39.06617 |
| 26 | rs365663 | G | A | 0.45495 | -0.00928 | 0.00161 | 8.10E-09 | 33.24589 |
| 27 | rs6889592 | A | G | 0.332608 | 0.011766 | 0.001697 | 4.10E-12 | 48.06987 |
| 28 | rs151014368 | A | G | 0.20734 | 0.011379 | 0.001988 | 1.00E-08 | 32.75743 |
| 29 | rs9382445 | C | T | 0.375168 | -0.00948 | 0.001649 | 8.90E-09 | 33.07661 |
| 30 | rs1611719 | A | G | 0.209698 | -0.01321 | 0.002027 | 7.10E-11 | 42.50004 |
| 31 | rs113113059 | C | T | 0.219834 | -0.01113 | 0.001933 | 8.60E-09 | 33.12921 |
| 32 | rs7740402 | G | T | 0.3061 | -0.00951 | 0.001735 | 4.10E-08 | 30.09031 |
| 33 | rs9345234 | C | A | 0.57786 | 0.009192 | 0.001624 | 1.50E-08 | 32.05745 |
| 34 | rs34556183 | G | A | 0.279888 | -0.01335 | 0.001782 | 6.60E-14 | 56.18077 |
| 35 | rs11982852 | T | C | 0.243858 | -0.01173 | 0.001862 | 3.10E-10 | 39.63641 |
| 36 | rs62444917 | C | A | 0.222315 | 0.012963 | 0.001926 | 1.70E-11 | 45.29037 |
| 37 | rs2079070 | G | C | 0.734582 | -0.01344 | 0.001811 | 1.20E-13 | 55.05182 |
| 38 | rs7831557 | A | G | 0.517438 | -0.01057 | 0.001601 | 4.20E-11 | 43.53272 |
| 39 | rs7016314 | C | T | 0.655927 | 0.010001 | 0.001688 | 3.10E-09 | 35.09354 |
| 40 | rs17391944 | G | T | 0.04986 | 0.021852 | 0.003724 | 4.40E-09 | 34.43123 |
| 41 | rs112100783 | A | G | 0.033439 | -0.02529 | 0.004549 | 2.70E-08 | 30.9259 |
| 42 | rs10510128 | A | G | 0.207951 | 0.011403 | 0.001974 | 7.70E-09 | 33.35917 |
| 43 | rs2236295 | T | G | 0.403043 | -0.00908 | 0.001636 | 2.90E-08 | 30.76193 |
| 44 | rs1517572 | C | A | 0.581256 | 0.011659 | 0.001622 | 6.50E-13 | 51.67849 |
| 45 | rs7115856 | C | A | 0.461273 | 0.010819 | 0.001603 | 1.50E-11 | 45.56391 |
| 46 | rs11039216 | T | C | 0.532944 | -0.01028 | 0.001604 | 1.50E-10 | 41.06165 |
| 47 | rs2734831 | G | T | 0.606881 | -0.0098 | 0.001639 | 2.20E-09 | 35.75172 |
| 48 | rs174564 | G | A | 0.348602 | 0.009745 | 0.001678 | 6.30E-09 | 33.72951 |
| 49 | rs1939455 | T | G | 0.120291 | -0.0158 | 0.002517 | 3.50E-10 | 39.39315 |
| 50 | rs1553132 | G | A | 0.258638 | 0.010526 | 0.001825 | 8.10E-09 | 33.2532 |
| 51 | rs34354917 | A | C | 0.288625 | -0.01002 | 0.001768 | 1.40E-08 | 32.12088 |
| 52 | rs4767550 | G | A | 0.413171 | 0.010873 | 0.001633 | 2.70E-11 | 44.35411 |
| 53 | rs6561715 | A | T | 0.630636 | 0.009782 | 0.001661 | 3.90E-09 | 34.68536 |
| 54 | rs55658675 | T | C | 0.352906 | -0.00969 | 0.001675 | 7.10E-09 | 33.51381 |
| 55 | rs11621908 | T | C | 0.082807 | -0.01999 | 0.002943 | 1.10E-11 | 46.10501 |
| 56 | rs2748809 | C | T | 0.429258 | -0.00925 | 0.001645 | 1.90E-08 | 31.62421 |
| 57 | rs8038326 | G | A | 0.273169 | -0.01338 | 0.001793 | 8.40E-14 | 55.71152 |
| 58 | rs56367859 | G | A | 0.397585 | 0.011622 | 0.001636 | 1.20E-12 | 50.47308 |
| 59 | rs9302680 | A | G | 0.439272 | 0.012044 | 0.001611 | 7.60E-14 | 55.91176 |
| 60 | rs11643715 | G | C | 0.292625 | 0.010949 | 0.00176 | 5.00E-10 | 38.68786 |
| 61 | rs8047587 | T | G | 0.439514 | -0.01102 | 0.001613 | 8.30E-12 | 46.68423 |
| 62 | rs72771082 | G | A | 0.217832 | 0.010974 | 0.001936 | 1.40E-08 | 32.13451 |
| 63 | rs8074498 | A | T | 0.580951 | -0.00933 | 0.001634 | 1.10E-08 | 32.59478 |
| 64 | rs11650677 | A | G | 0.339185 | 0.01117 | 0.00169 | 3.80E-11 | 43.70498 |
| 65 | rs9903898 | T | C | 0.488883 | -0.00945 | 0.001601 | 3.60E-09 | 34.82573 |
| 66 | rs8072993 | G | T | 0.635677 | 0.010922 | 0.001992 | 4.20E-08 | 30.04916 |
| 67 | rs1348047 | T | G | 0.267253 | -0.01264 | 0.00182 | 3.80E-12 | 48.22308 |
| 68 | rs35126035 | C | A | 0.55833 | -0.0092 | 0.001644 | 2.20E-08 | 31.2943 |
| 69 | rs34786000 | T | G | 0.553361 | 0.010958 | 0.001628 | 1.70E-11 | 45.30973 |
| 70 | rs2072727 | C | T | 0.56459 | -0.00927 | 0.001614 | 9.30E-09 | 32.98056 |
| 71 | rs9611007 | T | C | 0.141673 | -0.01359 | 0.002297 | 3.30E-09 | 35.01711 |

SNP: Single nucleotide polymorphisms; EAF: Effect allele frequency; SE：Standard error. F-statistics= (Effect/SE)^2^

^a^ Effect size of each allele on genetically-determined sleep duration (hours per allele)

^b^ Standard error of the genetic association of each effect allele with genetically-determined sleep duration

## Table S10. Characteristics of SNPs used as instrumental variables for genetically-determined WHR.

|  | SNP | Effect allele | Other allele | EAF | Z_k_^a^ | σZ_k_^b^ | *P*-value | F-statistics |
| --- | --- | --- | --- | --- | --- | --- | --- | --- |
| 1 | rs4846565 | A | G | 0.3333 | -0.048 | 0.0046 | 3.70E-25 | 108.8847 |
| 2 | rs4471313 | T | G | 0.7232 | 0.03 | 0.0052 | 7.50E-09 | 33.28402 |
| 3 | rs2765539 | T | C | 0.7083 | 0.031 | 0.005 | 2.80E-10 | 38.44 |
| 4 | rs10195252 | C | T | 0.4417 | -0.044 | 0.0044 | 1.00E-23 | 100 |
| 5 | rs17819328 | G | T | 0.45 | 0.032 | 0.0046 | 1.80E-12 | 48.39319 |
| 6 | rs9860730 | G | A | 0.2333 | -0.042 | 0.0048 | 3.80E-18 | 76.5625 |
| 7 | rs10804591 | A | C | 0.85 | 0.035 | 0.0054 | 1.20E-10 | 42.0096 |
| 8 | rs9687846 | A | G | 0.1917 | 0.034 | 0.0057 | 3.40E-09 | 35.58018 |
| 9 | rs1045241 | T | C | 0.3083 | -0.027 | 0.0049 | 3.50E-08 | 30.36235 |
| 10 | rs1936805 | T | C | 0.55 | 0.044 | 0.0044 | 7.00E-24 | 100 |
| 11 | rs1358980 | T | C | 0.45 | 0.049 | 0.0048 | 2.40E-24 | 104.2101 |
| 12 | rs1294421 | G | T | 0.6 | 0.033 | 0.0046 | 8.30E-13 | 51.46503 |
| 13 | rs3902751 | A | G | 0.225 | 0.032 | 0.0051 | 6.40E-10 | 39.36947 |
| 14 | rs12679556 | G | T | 0.2083 | 0.03 | 0.0052 | 7.40E-09 | 33.28402 |
| 15 | rs11989744 | T | C | 0.2583 | -0.035 | 0.0061 | 5.40E-09 | 32.92126 |
| 16 | rs1394461 | C | G | 0.2583 | 0.035 | 0.0063 | 3.60E-08 | 30.8642 |
| 17 | rs863750 | T | C | 0.5333 | 0.027 | 0.0045 | 1.00E-09 | 36 |
| 18 | rs11048470 | T | G | 0.2333 | 0.033 | 0.0049 | 1.90E-11 | 45.3561 |
| 19 | rs1443512 | C | A | 0.8 | -0.037 | 0.0051 | 2.40E-13 | 52.6336 |
| 20 | rs11075985 | A | C | 0.4833 | 0.039 | 0.0045 | 5.00E-18 | 75.11111 |
| 21 | rs4646404 | A | G | 0.375 | -0.03 | 0.0051 | 3.10E-09 | 34.60208 |
| 22 | rs489693 | A | C | 0.4083 | 0.029 | 0.0047 | 1.00E-09 | 38.07153 |
| 23 | rs2179129 | G | A | 0.45 | -0.026 | 0.0045 | 1.20E-08 | 33.38272 |

SNPs: Single nucleotide polymorphisms; WHR: Waist-to-hip ratio; EAF: Effect allele frequency; SE：Standard error. F-statistics= (Effect/SE)^2^

^a^ Effect size of each allele on genetically-determined WHR (percentage point/ allele)

^b^ Standard error of the genetic association of each effect allele with genetically-determined WHR

## Table S11. Characteristics of SNPs used as instrumental variables for genetically-determined FBG.

|  | SNP | Effect allele | Other allele | EAF | Z_k_^a^ | σZ_k_^b^ | *P*-value | F-statistics |
| --- | --- | --- | --- | --- | --- | --- | --- | --- |
| 1 | rs780093 | C | T | 0.606 | 0.027 | 0.0037 | 2.91E-13 | 53.25055 |
| 2 | rs560887 | C | T | 0.674 | 0.075 | 0.0041 | 4.61E-75 | 334.6222 |
| 3 | rs11717195 | C | T | 0.77 | -0.029 | 0.0047 | 1.11E-09 | 38.07153 |
| 4 | rs2191349 | T | G | 0.467 | 0.03 | 0.0036 | 7.83E-17 | 69.44444 |
| 5 | rs10276674 | C | T | 0.836 | 0.036 | 0.0051 | 1.75E-12 | 49.82699 |
| 6 | rs6975024 | C | T | 0.8 | 0.062 | 0.0049 | 5.54E-37 | 160.1 |
| 7 | rs11558471 | G | A | 0.748 | -0.027 | 0.004 | 2.62E-11 | 45.5625 |
| 8 | rs4506565 | T | A | 0.704 | 0.023 | 0.004 | 1.24E-08 | 33.0625 |
| 9 | rs10787312 | A | G | 0.078 | 0.042 | 0.0062 | 1.15E-11 | 45.8897 |
| 10 | rs7944584 | T | A | 0.712 | -0.025 | 0.0041 | 1.47E-09 | 37.18025 |
| 11 | rs2524299 | T | A | 0.892 | -0.03 | 0.0052 | 3.60E-09 | 33.28402 |
| 12 | rs10830963 | G | C | 0.7 | 0.079 | 0.0045 | 1.26E-68 | 308.1975 |
| 13 | rs12805422 | A | G | 0.5 | -0.023 | 0.0037 | 1.18E-09 | 38.64134 |
| 14 | rs17390909 | G | C | 0.883 | -0.037 | 0.0067 | 3.40E-08 | 30.49677 |

SNP: Single nucleotide polymorphisms; FBG: Fasting blood glucose; EAF: Effect allele frequency; SE：Standard error. F-statistics= (Effect/SE)^2^

^a^ Effect size of each allele on genetically-determined FBG (mmol/l per allele)

^b^ Standard error of the genetic association of each effect allele with genetically-determined FBG

## Table S12. Characteristics of SNPs used as instrumental variables for genetically-determined HDL.

|  | SNP | Effect allele | Other allele | EAF | Z_k_^a^ | σZ_k_^b^ | *P*-value | F-statistics |
| --- | --- | --- | --- | --- | --- | --- | --- | --- |
| 1 | rs6544366 | T | G | 0.267874 | 0.064093 | 0.011001 | 8.14E-09 | 33.94352 |
| 2 | rs59347135 | G | C | 0.043114 | -0.16729 | 0.028587 | 6.99E-09 | 34.24585 |
| 3 | rs291 | C | T | 0.231014 | 0.10065 | 0.011676 | 1.45E-17 | 74.30865 |
| 4 | rs1883025 | T | C | 0.193654 | -0.07216 | 0.012455 | 9.85E-09 | 33.56274 |
| 5 | rs174583 | T | C | 0.392152 | -0.07783 | 0.010132 | 2.92E-14 | 59.00854 |
| 6 | rs964184 | C | G | 0.861774 | 0.081863 | 0.014048 | 8.07E-09 | 33.95833 |
| 7 | rs67053123 | A | T | 0.148501 | 0.092051 | 0.014556 | 3.90E-10 | 39.99198 |
| 8 | rs261291 | C | T | 0.373198 | 0.179384 | 0.010165 | 2.39E-68 | 311.4244 |
| 9 | rs60439253 | T | C | 0.035228 | 0.235306 | 0.028232 | 1.61E-16 | 69.46767 |
| 10 | rs11632618 | A | G | 0.059603 | 0.215139 | 0.023086 | 2.90E-20 | 86.84422 |
| 11 | rs111543310 | C | T | 0.015958 | 0.337634 | 0.048705 | 6.88E-12 | 48.05574 |
| 12 | rs247617 | A | C | 0.297513 | 0.209633 | 0.01079 | 1.93E-82 | 377.4648 |
| 13 | rs6507939 | C | A | 0.843394 | 0.094757 | 0.013468 | 3.34E-12 | 49.50128 |
| 14 | rs429358 | C | T | 0.169994 | -0.09155 | 0.013419 | 1.46E-11 | 46.54841 |
| 15 | rs6065904 | A | G | 0.228686 | -0.13534 | 0.011671 | 1.72E-30 | 134.4694 |

SNP: Single nucleotide polymorphisms; HDL: High-density lipoprotein; EAF: Effect allele frequency; SE：Standard error. F-statistics= (Effect/SE)^2^

^a^ Effect size of each allele on genetically-determined HDL (mmol/l per allele)

^b^ Standard error of the genetic association of each effect allele with genetically-determined HDL

## Table S13. Characteristics of SNPs used as instrumental variables for genetically-determined age that started HRT.

|  | SNP | Effect allele | Other allele | EAF | Z_k_^a^ | σZ_k_^b^ | *P*-value | F-statistics |
| --- | --- | --- | --- | --- | --- | --- | --- | --- |
| 1 | rs4532801 | G | T | 0.681815 | -0.03639 | 0.005081 | 8.00E-13 | 51.28849 |
| 2 | rs6760857 | A | G | 0.105071 | -0.04644 | 0.007732 | 1.90E-09 | 36.06509 |
| 3 | rs6760293 | T | A | 0.622675 | 0.028591 | 0.004889 | 5.00E-09 | 34.19953 |
| 4 | rs3774751 | T | G | 0.45985 | 0.029578 | 0.00477 | 5.60E-10 | 38.44701 |
| 5 | rs4235062 | A | G | 0.516176 | 0.033026 | 0.004755 | 3.80E-12 | 48.24565 |
| 6 | rs12503643 | T | G | 0.396197 | 0.028498 | 0.004856 | 4.40E-09 | 34.44714 |
| 7 | rs3765637 | T | A | 0.230577 | -0.03465 | 0.005628 | 7.40E-10 | 37.91105 |
| 8 | rs274721 | T | C | 0.379393 | -0.02823 | 0.004877 | 7.10E-09 | 33.51283 |
| 9 | rs251848 | A | G | 0.476717 | 0.031416 | 0.004847 | 9.10E-11 | 42.00133 |
| 10 | rs9348724 | G | C | 0.82855 | -0.03823 | 0.00636 | 1.80E-09 | 36.13356 |
| 11 | rs2844466 | C | T | 0.358737 | -0.03535 | 0.004944 | 8.60E-13 | 51.13631 |
| 12 | rs75779608 | T | C | 0.20262 | -0.03283 | 0.005893 | 2.50E-08 | 31.04451 |
| 13 | rs28797500 | C | T | 0.780783 | -0.04301 | 0.005733 | 6.30E-14 | 56.2833 |
| 14 | rs6980805 | C | G | 0.606067 | 0.028853 | 0.004865 | 3.00E-09 | 35.17361 |
| 15 | rs75770066 | G | A | 0.031184 | 0.103467 | 0.013594 | 2.70E-14 | 57.93161 |
| 16 | rs77100210 | C | A | 0.051122 | 0.059315 | 0.010869 | 4.80E-08 | 29.78357 |
| 17 | rs7994166 | T | C | 0.464875 | 0.028464 | 0.004768 | 2.40E-09 | 35.63745 |
| 18 | rs177404 | C | G | 0.687941 | -0.02959 | 0.005308 | 2.50E-08 | 31.0801 |
| 19 | rs6496571 | C | A | 0.382323 | -0.02835 | 0.004878 | 6.10E-09 | 33.78882 |
| 20 | rs3743590 | A | C | 0.62112 | -0.03746 | 0.004893 | 1.90E-14 | 58.61121 |
| 21 | rs732084 | C | A | 0.668218 | 0.030272 | 0.005055 | 2.10E-09 | 35.85862 |
| 22 | rs11668344 | G | A | 0.365523 | -0.05402 | 0.004908 | 3.60E-28 | 121.1351 |
| 23 | rs2013097 | C | T | 0.443981 | -0.02684 | 0.004801 | 2.30E-08 | 31.23801 |
| 24 | rs16991615 | A | G | 0.061184 | 0.105051 | 0.009857 | 1.60E-26 | 113.581 |

SNP: Single nucleotide polymorphisms; HRT: Hormone-replacement therapy; EAF: Effect allele frequency; SE：Standard error. F-statistics= (Effect/SE)^2^

^a^ Effect size of each allele on genetically-determined age that started HRT (years/allele)

^b^ Standard error of the genetic association of each effect allele with genetically-determined age that started HRT

Table S14. Genetic estimates for the association of genetically predicted AAM with genetically-determined current smoking behavior.

|  | SNPs | Effect allele | X_k_^a^ | σX_k_^b^ | Z_k_^c^ | σZ_k_^d^ |
| --- | --- | --- | --- | --- | --- | --- |
| 1 | rs10144321 | G | -0.042 | 0.006618 | -0.00073 | 0.001572 |
| 2 | rs10483727 | C | -0.037 | 0.005785 | 0.001966 | 0.001357 |
| 3 | rs1079866 | G | 0.072 | 0.008444 | -0.00341 | 0.001948 |
| 4 | rs10840031 | A | 0.038 | 0.006406 | 0.003858 | 0.001429 |
| 5 | rs10938397 | G | -0.038 | 0.005923 | -0.00247 | 0.001336 |
| 6 | rs11022756 | C | -0.048 | 0.006481 | -0.00104 | 0.001458 |
| 7 | rs11715566 | T | 0.052 | 0.005631 | -0.00053 | 0.001322 |
| 8 | rs11756454 | A | 0.034 | 0.005703 | 0.001462 | 0.00133 |
| 9 | rs11767400 | A | 0.035 | 0.006295 | 0.003383 | 0.001437 |
| 10 | rs12003641 | T | 0.082 | 0.010555 | 0.002771 | 0.002398 |
| 11 | rs12148769 | A | -0.055 | 0.009752 | 0.000276 | 0.002141 |
| 12 | rs12291726 | G | 0.057 | 0.008333 | 0.000222 | 0.001802 |
| 13 | rs12598642 | G | 0.044 | 0.005681 | -0.0031 | 0.001342 |
| 14 | rs12915845 | T | -0.035 | 0.005729 | 0.000366 | 0.001339 |
| 15 | rs13179411 | T | 0.06 | 0.007813 | -0.00316 | 0.00185 |
| 16 | rs13215865 | T | -0.042 | 0.007472 | 0.004965 | 0.00165 |
| 17 | rs1398217 | C | 0.046 | 0.005767 | -0.00093 | 0.001335 |
| 18 | rs1482853 | A | -0.038 | 0.006043 | 0.002708 | 0.001354 |
| 19 | rs1516883 | A | -0.091 | 0.002455 | 0.005914 | 0.001427 |
| 20 | rs1518080 | G | -0.051 | 0.006004 | 0.000337 | 0.001383 |
| 21 | rs1659127 | A | 0.044 | 0.006372 | -0.00197 | 0.001405 |
| 22 | rs16938437 | T | -0.067 | 0.010348 | -0.00034 | 0.002287 |
| 23 | rs17351680 | G | 0.044 | 0.007557 | 0.000494 | 0.001815 |
| 24 | rs1874984 | C | 0.037 | 0.006005 | 0.000137 | 0.00132 |
| 25 | rs2153127 | C | -0.077 | 0.002077 | -0.00094 | 0.001325 |
| 26 | rs2179786 | T | -0.039 | 0.00572 | -0.00119 | 0.001339 |
| 27 | rs2184968 | C | -0.036 | 0.005672 | -0.00099 | 0.001327 |
| 28 | rs2303100 | T | 0.038 | 0.005684 | 0.002517 | 0.001328 |
| 29 | rs2344508 | A | 0.034 | 0.005747 | -0.00417 | 0.001333 |
| 30 | rs2617056 | T | -0.036 | 0.005933 | 0.002932 | 0.001353 |
| 31 | rs2687729 | G | 0.044 | 0.006575 | -0.00239 | 0.001497 |
| 32 | rs2836950 | G | -0.035 | 0.006206 | -0.00043 | 0.00138 |
| 33 | rs2947411 | G | -0.052 | 0.007655 | 0.006326 | 0.00174 |
| 34 | rs3115627 | G | 0.038 | 0.006453 | 0.000256 | 0.001395 |
| 35 | rs3733632 | G | 0.049 | 0.007862 | -0.00221 | 0.001848 |
| 36 | rs3743266 | C | -0.045 | 0.006233 | -0.00038 | 0.001405 |
| 37 | rs3870341 | G | -0.043 | 0.006449 | -0.00524 | 0.001507 |
| 38 | rs3914188 | C | 0.044 | 0.006702 | 0.000146 | 0.001493 |
| 39 | rs4242496 | A | -0.033 | 0.005696 | 0.001219 | 0.001335 |
| 40 | rs4369815 | G | -0.08 | 0.011888 | 0.002678 | 0.002666 |
| 41 | rs466639 | C | 0.075 | 0.008706 | -0.00269 | 0.001996 |
| 42 | rs4801589 | G | 0.032 | 0.005628 | 0.000591 | 0.001324 |
| 43 | rs4840086 | G | -0.036 | 0.00562 | -0.0022 | 0.001333 |
| 44 | rs618678 | T | -0.034 | 0.006148 | -0.00491 | 0.001408 |
| 45 | rs633715 | C | -0.051 | 0.00725 | 0.001832 | 0.001612 |
| 46 | rs6694738 | A | -0.044 | 0.007885 | -0.00684 | 0.001892 |
| 47 | rs6747380 | A | 0.065 | 0.007658 | 0.003059 | 0.001769 |
| 48 | rs6758290 | C | -0.04 | 0.006322 | 0.000464 | 0.001326 |
| 49 | rs6770162 | A | 0.036 | 0.005733 | 0.000472 | 0.001326 |
| 50 | rs6933660 | A | -0.036 | 0.006331 | -0.00163 | 0.001428 |
| 51 | rs7103411 | T | -0.043 | 0.007056 | 0.00514 | 0.001625 |
| 52 | rs7119712 | A | -0.041 | 0.006445 | 0.001066 | 0.001514 |
| 53 | rs740077 | C | -0.046 | 0.007045 | -0.00319 | 0.001642 |
| 54 | rs7642134 | G | 0.038 | 0.005912 | -0.00157 | 0.001362 |
| 55 | rs7821178 | A | -0.045 | 0.006196 | 0.002476 | 0.001397 |
| 56 | rs7853970 | C | -0.037 | 0.00626 | 0.004275 | 0.001338 |
| 57 | rs7944630 | A | 0.047 | 0.005728 | -0.00108 | 0.001342 |
| 58 | rs852069 | G | 0.036 | 0.005907 | -0.0008 | 0.001378 |
| 59 | rs888345 | A | -0.044 | 0.007315 | 0.002803 | 0.001672 |
| 60 | rs895526 | C | 0.044 | 0.007553 | -0.00486 | 0.00169 |
| 61 | rs913588 | A | -0.034 | 0.005683 | 0.00114 | 0.001321 |
| 62 | rs9373571 | A | 0.034 | 0.005926 | -0.00191 | 0.001329 |
| 63 | rs9555810 | G | 0.047 | 0.006492 | -0.00154 | 0.001461 |
| 64 | rs9565073 | C | 0.034 | 0.005881 | 0.001269 | 0.001333 |
| 65 | rs9635759 | A | 0.058 | 0.006362 | -0.00123 | 0.001467 |
| 66 | rs9647570 | G | 0.046 | 0.008427 | -0.00137 | 0.001908 |
| 67 | rs9939609 | A | -0.042 | 0.00572 | -0.00053 | 0.001353 |
| 68 | rs9997604 | C | 0.039 | 0.006511 | -0.00132 | 0.001489 |

AAM: Age at menarche; SNPs: Single nucleotide polymorphisms.

^a^ Effect size per allele in AAM (in years)

^b^ Standard error of the genetic association of each effect allele with genetically predicted AAM

^c^ Effect size of each allele on genetically-determined current smoking behavior (pack-years per allele)

^d^ Standard error of the genetic association of each effect allele with genetically-determined current smoking behavior

Table S15. Genetic estimates for the association of genetically predicted AAM with genetically-determined HbA1c.

|  | SNPs | Effect allele | X_k_^a^ | σX_k_^b^ | Z_k_^c^ | σZ_k_^d^ |
| --- | --- | --- | --- | --- | --- | --- |
| 1 | rs10144321 | G | -0.042 | 0.006618 | 0.005119 | 0.018249 |
| 2 | rs10483727 | C | -0.037 | 0.005785 | 0.007035 | 0.015756 |
| 3 | rs1079866 | G | 0.072 | 0.008444 | 0.024514 | 0.022608 |
| 4 | rs10840031 | A | 0.038 | 0.006406 | -0.04251 | 0.016581 |
| 5 | rs10938397 | G | -0.038 | 0.005923 | 0.039826 | 0.015508 |
| 6 | rs11022756 | C | -0.048 | 0.006481 | 0.019009 | 0.016921 |
| 7 | rs11715566 | T | 0.052 | 0.005631 | 0.009609 | 0.015349 |
| 8 | rs11756454 | A | 0.034 | 0.005703 | -0.01029 | 0.015437 |
| 9 | rs11767400 | A | 0.035 | 0.006295 | 0.012447 | 0.016671 |
| 10 | rs12003641 | T | 0.082 | 0.010555 | 0.038036 | 0.02794 |
| 11 | rs12148769 | A | -0.055 | 0.009752 | -0.0097 | 0.024886 |
| 12 | rs12291726 | G | 0.057 | 0.008333 | -0.08928 | 0.020919 |
| 13 | rs12598642 | G | 0.044 | 0.005681 | -0.08606 | 0.015588 |
| 14 | rs12915845 | T | -0.035 | 0.005729 | -0.00497 | 0.015546 |
| 15 | rs13179411 | T | 0.06 | 0.007813 | 0.040774 | 0.021464 |
| 16 | rs13215865 | T | -0.042 | 0.007472 | -0.0073 | 0.019148 |
| 17 | rs1398217 | C | 0.046 | 0.005767 | -0.02294 | 0.015504 |
| 18 | rs1482853 | A | -0.038 | 0.006043 | -0.00617 | 0.015737 |
| 19 | rs1516883 | A | -0.091 | 0.002455 | 0.031453 | 0.016582 |
| 20 | rs1518080 | G | -0.051 | 0.006004 | -0.01873 | 0.016055 |
| 21 | rs1659127 | A | 0.044 | 0.006372 | -0.00959 | 0.016316 |
| 22 | rs16938437 | T | -0.067 | 0.010348 | -0.03637 | 0.026587 |
| 23 | rs17351680 | G | 0.044 | 0.007557 | -0.03783 | 0.021084 |
| 24 | rs1874984 | C | 0.037 | 0.006005 | 0.022379 | 0.015326 |
| 25 | rs2153127 | C | -0.077 | 0.002077 | -0.01583 | 0.015387 |
| 26 | rs2179786 | T | -0.039 | 0.00572 | -0.03001 | 0.015543 |
| 27 | rs2184968 | C | -0.036 | 0.005672 | 0.10548 | 0.015411 |
| 28 | rs2303100 | T | 0.038 | 0.005684 | 0.008501 | 0.015415 |
| 29 | rs2344508 | A | 0.034 | 0.005747 | 0.001825 | 0.015474 |
| 30 | rs2617056 | T | -0.036 | 0.005933 | 0.011943 | 0.015704 |
| 31 | rs2687729 | G | 0.044 | 0.006575 | 0.033207 | 0.017381 |
| 32 | rs2836950 | G | -0.035 | 0.006206 | 0.03456 | 0.016009 |
| 33 | rs2947411 | G | -0.052 | 0.007655 | 0.085049 | 0.02021 |
| 34 | rs3115627 | G | 0.038 | 0.006453 | 0.011476 | 0.016213 |
| 35 | rs3733632 | G | 0.049 | 0.007862 | -0.04022 | 0.021453 |
| 36 | rs3743266 | C | -0.045 | 0.006233 | -0.00682 | 0.016309 |
| 37 | rs3870341 | G | -0.043 | 0.006449 | 0.12777 | 0.017514 |
| 38 | rs3914188 | C | 0.044 | 0.006702 | -0.03788 | 0.017418 |
| 39 | rs4242496 | A | -0.033 | 0.005696 | -0.02322 | 0.015502 |
| 40 | rs4369815 | G | -0.08 | 0.011888 | -0.01436 | 0.030921 |
| 41 | rs466639 | C | 0.075 | 0.008706 | 0.002469 | 0.023231 |
| 42 | rs4801589 | G | 0.032 | 0.005628 | -0.03406 | 0.015372 |
| 43 | rs4840086 | G | -0.036 | 0.00562 | -0.00207 | 0.015488 |
| 44 | rs618678 | T | -0.034 | 0.006148 | -0.01053 | 0.016347 |
| 45 | rs633715 | C | -0.051 | 0.00725 | 0.047268 | 0.018726 |
| 46 | rs6694738 | A | -0.044 | 0.007885 | -0.00257 | 0.021988 |
| 47 | rs6747380 | A | 0.065 | 0.007658 | 0.036375 | 0.020554 |
| 48 | rs6758290 | C | -0.04 | 0.006322 | 0.016483 | 0.015393 |
| 49 | rs6770162 | A | 0.036 | 0.005733 | 0.010954 | 0.015394 |
| 50 | rs6933660 | A | -0.036 | 0.006331 | -0.00257 | 0.01657 |
| 51 | rs7103411 | T | -0.043 | 0.007056 | 0.059411 | 0.018892 |
| 52 | rs7119712 | A | -0.041 | 0.006445 | 0.007097 | 0.017618 |
| 53 | rs740077 | C | -0.046 | 0.007045 | 0.036143 | 0.019084 |
| 54 | rs7642134 | G | 0.038 | 0.005912 | 0.010299 | 0.015809 |
| 55 | rs7821178 | A | -0.045 | 0.006196 | -0.01094 | 0.016225 |
| 56 | rs7853970 | C | -0.037 | 0.00626 | 0.036013 | 0.015543 |
| 57 | rs7944630 | A | 0.047 | 0.005728 | 0.024113 | 0.015582 |
| 58 | rs852069 | G | 0.036 | 0.005907 | 0.000734 | 0.015994 |
| 59 | rs888345 | A | -0.044 | 0.007315 | 0.02875 | 0.01944 |
| 60 | rs895526 | C | 0.044 | 0.007553 | -0.04848 | 0.019653 |
| 61 | rs913588 | A | -0.034 | 0.005683 | 0.011924 | 0.015334 |
| 62 | rs9373571 | A | 0.034 | 0.005926 | -0.00597 | 0.015425 |
| 63 | rs9555810 | G | 0.047 | 0.006492 | -0.0225 | 0.016951 |
| 64 | rs9565073 | C | 0.034 | 0.005881 | -0.03196 | 0.01548 |
| 65 | rs9635759 | A | 0.058 | 0.006362 | 0.000303 | 0.017013 |
| 66 | rs9647570 | G | 0.046 | 0.008427 | -0.01043 | 0.022107 |
| 67 | rs9939609 | A | -0.042 | 0.00572 | 0.1502 | 0.015703 |
| 68 | rs9997604 | C | 0.039 | 0.006511 | -0.02668 | 0.017283 |

AAM: Age at menarche; HbA1c: Hemoglobin A1c; SNPs: Single nucleotide polymorphisms.

^a^ Effect size per allele in AAM (in years)

^b^ Standard error of the genetic association of each effect allele with genetically predicted AAM

^c^ Effect size of each allele on genetically-determined HbA1c (percentage point per allele)

^d^ Standard error of the genetic association of each effect allele with genetically-determined HbA1c

Table S16. Genetic estimates for the association of genetically predicted AAM with genetically-determined SBP.

|  | SNPs | Effect allele | X_k_^a^ | σX_k_^b^ | Z_k_^c^ | σZ_k_^d^ |
| --- | --- | --- | --- | --- | --- | --- |
| 1 | rs10144321 | G | -0.042 | 0.006618 | 0.000506 | 0.002924 |
| 2 | rs10483727 | C | -0.037 | 0.005785 | -0.00786 | 0.002527 |
| 3 | rs1079866 | G | 0.072 | 0.008444 | -0.00013 | 0.003629 |
| 4 | rs10840031 | A | 0.038 | 0.006406 | -0.01064 | 0.002659 |
| 5 | rs10938397 | G | -0.038 | 0.005923 | 0.004309 | 0.002487 |
| 6 | rs11022756 | C | -0.048 | 0.006481 | 0.011908 | 0.002715 |
| 7 | rs11715566 | T | 0.052 | 0.005631 | 0.005521 | 0.002462 |
| 8 | rs11756454 | A | 0.034 | 0.005703 | -0.00201 | 0.002475 |
| 9 | rs11767400 | A | 0.035 | 0.006295 | 0.001564 | 0.002675 |
| 10 | rs12003641 | T | 0.082 | 0.010555 | -0.0007 | 0.00446 |
| 11 | rs12148769 | A | -0.055 | 0.009752 | 0.001773 | 0.003987 |
| 12 | rs12291726 | G | 0.057 | 0.008333 | -0.00535 | 0.003358 |
| 13 | rs12598642 | G | 0.044 | 0.005681 | -0.00191 | 0.002497 |
| 14 | rs12915845 | T | -0.035 | 0.005729 | 0.00864 | 0.002494 |
| 15 | rs13179411 | T | 0.06 | 0.007813 | -0.00619 | 0.003447 |
| 16 | rs13215865 | T | -0.042 | 0.007472 | 0.002239 | 0.003071 |
| 17 | rs1398217 | C | 0.046 | 0.005767 | -0.0038 | 0.002486 |
| 18 | rs1482853 | A | -0.038 | 0.006043 | -0.00682 | 0.00252 |
| 19 | rs1516883 | A | -0.091 | 0.002455 | -0.00166 | 0.002655 |
| 20 | rs1518080 | G | -0.051 | 0.006004 | -0.00421 | 0.002574 |
| 21 | rs1659127 | A | 0.044 | 0.006372 | -0.00363 | 0.002616 |
| 22 | rs16938437 | T | -0.067 | 0.010348 | -0.01592 | 0.004254 |
| 23 | rs17351680 | G | 0.044 | 0.007557 | -0.00976 | 0.00338 |
| 24 | rs1874984 | C | 0.037 | 0.006005 | -0.00376 | 0.002456 |
| 25 | rs2153127 | C | -0.077 | 0.002077 | -0.00443 | 0.002465 |
| 26 | rs2179786 | T | -0.039 | 0.00572 | 0.00136 | 0.002491 |
| 27 | rs2184968 | C | -0.036 | 0.005672 | 0.002656 | 0.002472 |
| 28 | rs2303100 | T | 0.038 | 0.005684 | -0.0039 | 0.002472 |
| 29 | rs2344508 | A | 0.034 | 0.005747 | 0.002448 | 0.002483 |
| 30 | rs2617056 | T | -0.036 | 0.005933 | -0.00149 | 0.002518 |
| 31 | rs2687729 | G | 0.044 | 0.006575 | -0.00617 | 0.002787 |
| 32 | rs2836950 | G | -0.035 | 0.006206 | 0.001986 | 0.002569 |
| 33 | rs2947411 | G | -0.052 | 0.007655 | 0.016098 | 0.003239 |
| 34 | rs3115627 | G | 0.038 | 0.006453 | 0.002729 | 0.002596 |
| 35 | rs3733632 | G | 0.049 | 0.007862 | -0.00126 | 0.003438 |
| 36 | rs3743266 | C | -0.045 | 0.006233 | 0.001066 | 0.002616 |
| 37 | rs3870341 | G | -0.043 | 0.006449 | -0.00686 | 0.002805 |
| 38 | rs3914188 | C | 0.044 | 0.006702 | -0.00657 | 0.002779 |
| 39 | rs4242496 | A | -0.033 | 0.005696 | 0.002609 | 0.002485 |
| 40 | rs4369815 | G | -0.08 | 0.011888 | -0.00015 | 0.004961 |
| 41 | rs466639 | C | 0.075 | 0.008706 | -0.00325 | 0.003711 |
| 42 | rs4801589 | G | 0.032 | 0.005628 | 0.000434 | 0.002465 |
| 43 | rs4840086 | G | -0.036 | 0.00562 | 0.001213 | 0.002482 |
| 44 | rs618678 | T | -0.034 | 0.006148 | 0.00523 | 0.00262 |
| 45 | rs633715 | C | -0.051 | 0.00725 | 0.004275 | 0.003003 |
| 46 | rs6694738 | A | -0.044 | 0.007885 | -0.00408 | 0.003521 |
| 47 | rs6747380 | A | 0.065 | 0.007658 | -0.00011 | 0.003295 |
| 48 | rs6758290 | C | -0.04 | 0.006322 | -0.00453 | 0.002469 |
| 49 | rs6770162 | A | 0.036 | 0.005733 | 0.004957 | 0.002468 |
| 50 | rs6933660 | A | -0.036 | 0.006331 | 0.003538 | 0.002659 |
| 51 | rs7103411 | T | -0.043 | 0.007056 | -0.00342 | 0.003028 |
| 52 | rs7119712 | A | -0.041 | 0.006445 | 0.002661 | 0.002818 |
| 53 | rs740077 | C | -0.046 | 0.007045 | 0.005477 | 0.003052 |
| 54 | rs7642134 | G | 0.038 | 0.005912 | 0.000429 | 0.002535 |
| 55 | rs7821178 | A | -0.045 | 0.006196 | -0.00105 | 0.0026 |
| 56 | rs7853970 | C | -0.037 | 0.00626 | 0.000991 | 0.00249 |
| 57 | rs7944630 | A | 0.047 | 0.005728 | -0.00667 | 0.0025 |
| 58 | rs852069 | G | 0.036 | 0.005907 | -0.00174 | 0.002565 |
| 59 | rs888345 | A | -0.044 | 0.007315 | 0.00299 | 0.003109 |
| 60 | rs895526 | C | 0.044 | 0.007553 | -0.00118 | 0.003143 |
| 61 | rs913588 | A | -0.034 | 0.005683 | 0.00751 | 0.002457 |
| 62 | rs9373571 | A | 0.034 | 0.005926 | 0.001298 | 0.002475 |
| 63 | rs9555810 | G | 0.047 | 0.006492 | -0.00315 | 0.002719 |
| 64 | rs9565073 | C | 0.034 | 0.005881 | -0.00387 | 0.00248 |
| 65 | rs9635759 | A | 0.058 | 0.006362 | 0.002635 | 0.002731 |
| 66 | rs9647570 | G | 0.046 | 0.008427 | -0.00067 | 0.00355 |
| 67 | rs9939609 | A | -0.042 | 0.00572 | 0.009523 | 0.002519 |
| 68 | rs9997604 | C | 0.039 | 0.006511 | 0.006726 | 0.002772 |

AAM: Age at menarche; SBP: Systolic blood pressure; SNPs: Single nucleotide polymorphisms.

^a^ Effect size per allele in AAM (in years)

^b^ Standard error of the genetic association of each effect allele with genetically predicted AAM

^c^ Effect size of each allele on genetically-determined SBP (mmHg per allele)

^d^ Standard error of the genetic association of each effect allele with genetically-determined SBP

Table S17. Genetic estimates for the association of genetically predicted AAM with genetically-determined TG.

|  | SNPs | Effect allele | X_k_^a^ | σX_k_^b^ | Z_k_^c^ | σZ_k_^d^ |
| --- | --- | --- | --- | --- | --- | --- |
| 1 | rs10144321 | G | -0.042 | 0.006618 | -0.00456 | 0.00276 |
| 2 | rs10483727 | C | -0.037 | 0.005785 | 0.003553 | 0.002382 |
| 3 | rs1079866 | G | 0.072 | 0.008444 | -0.00722 | 0.003416 |
| 4 | rs10840031 | A | 0.038 | 0.006406 | -0.00351 | 0.002506 |
| 5 | rs10938397 | G | -0.038 | 0.005923 | -0.00175 | 0.002345 |
| 6 | rs11022756 | C | -0.048 | 0.006481 | 0.012954 | 0.002559 |
| 7 | rs11715566 | T | 0.052 | 0.005631 | -0.00635 | 0.002321 |
| 8 | rs11756454 | A | 0.034 | 0.005703 | 0.002398 | 0.002335 |
| 9 | rs11767400 | A | 0.035 | 0.006295 | 0.01044 | 0.00252 |
| 10 | rs12003641 | T | 0.082 | 0.010555 | -0.00542 | 0.004227 |
| 11 | rs12148769 | A | -0.055 | 0.009752 | -0.00087 | 0.003763 |
| 12 | rs12291726 | G | 0.057 | 0.008333 | -0.01792 | 0.003161 |
| 13 | rs12598642 | G | 0.044 | 0.005681 | -0.01762 | 0.002357 |
| 14 | rs12915845 | T | -0.035 | 0.005729 | 0.003798 | 0.002351 |
| 15 | rs13179411 | T | 0.06 | 0.007813 | 0.002173 | 0.003246 |
| 16 | rs13215865 | T | -0.042 | 0.007472 | 0.001691 | 0.002896 |
| 17 | rs1398217 | C | 0.046 | 0.005767 | -0.00047 | 0.002345 |
| 18 | rs1482853 | A | -0.038 | 0.006043 | -0.01618 | 0.002379 |
| 19 | rs1516883 | A | -0.091 | 0.002455 | 0.004665 | 0.002509 |
| 20 | rs1518080 | G | -0.051 | 0.006004 | 0.004652 | 0.002429 |
| 21 | rs1659127 | A | 0.044 | 0.006372 | 0.003358 | 0.002467 |
| 22 | rs16938437 | T | -0.067 | 0.010348 | -0.0053 | 0.004015 |
| 23 | rs17351680 | G | 0.044 | 0.007557 | -0.0078 | 0.003187 |
| 24 | rs1874984 | C | 0.037 | 0.006005 | -0.00106 | 0.002318 |
| 25 | rs2153127 | C | -0.077 | 0.002077 | -0.00173 | 0.002326 |
| 26 | rs2179786 | T | -0.039 | 0.00572 | -0.00283 | 0.002351 |
| 27 | rs2184968 | C | -0.036 | 0.005672 | -0.01373 | 0.002331 |
| 28 | rs2303100 | T | 0.038 | 0.005684 | 0.004141 | 0.002332 |
| 29 | rs2344508 | A | 0.034 | 0.005747 | 0.000764 | 0.00234 |
| 30 | rs2617056 | T | -0.036 | 0.005933 | 0.003369 | 0.002374 |
| 31 | rs2687729 | G | 0.044 | 0.006575 | -0.00016 | 0.00263 |
| 32 | rs2836950 | G | -0.035 | 0.006206 | 0.010947 | 0.002422 |
| 33 | rs2947411 | G | -0.052 | 0.007655 | 0.001788 | 0.003055 |
| 34 | rs3115627 | G | 0.038 | 0.006453 | 0.000873 | 0.002452 |
| 35 | rs3733632 | G | 0.049 | 0.007862 | 0.000262 | 0.003244 |
| 36 | rs3743266 | C | -0.045 | 0.006233 | 0.004181 | 0.002465 |
| 37 | rs3870341 | G | -0.043 | 0.006449 | 0.009642 | 0.00265 |
| 38 | rs3914188 | C | 0.044 | 0.006702 | -0.00458 | 0.002633 |
| 39 | rs4242496 | A | -0.033 | 0.005696 | -0.00443 | 0.002344 |
| 40 | rs4369815 | G | -0.08 | 0.011888 | 0.009008 | 0.004681 |
| 41 | rs466639 | C | 0.075 | 0.008706 | -0.00045 | 0.003512 |
| 42 | rs4801589 | G | 0.032 | 0.005628 | -0.0016 | 0.002325 |
| 43 | rs4840086 | G | -0.036 | 0.00562 | 0.00286 | 0.002344 |
| 44 | rs618678 | T | -0.034 | 0.006148 | -0.00079 | 0.002471 |
| 45 | rs633715 | C | -0.051 | 0.00725 | 0.000632 | 0.002832 |
| 46 | rs6694738 | A | -0.044 | 0.007885 | -0.00655 | 0.003326 |
| 47 | rs6747380 | A | 0.065 | 0.007658 | -0.00163 | 0.00311 |
| 48 | rs6758290 | C | -0.04 | 0.006322 | 0.000374 | 0.002328 |
| 49 | rs6770162 | A | 0.036 | 0.005733 | -0.00026 | 0.002327 |
| 50 | rs6933660 | A | -0.036 | 0.006331 | -0.00109 | 0.002505 |
| 51 | rs7103411 | T | -0.043 | 0.007056 | 0.010478 | 0.002858 |
| 52 | rs7119712 | A | -0.041 | 0.006445 | -0.00105 | 0.002663 |
| 53 | rs740077 | C | -0.046 | 0.007045 | -0.00765 | 0.002887 |
| 54 | rs7642134 | G | 0.038 | 0.005912 | 0.004661 | 0.00239 |
| 55 | rs7821178 | A | -0.045 | 0.006196 | 0.000554 | 0.002453 |
| 56 | rs7853970 | C | -0.037 | 0.00626 | 0.003595 | 0.00235 |
| 57 | rs7944630 | A | 0.047 | 0.005728 | 0.004215 | 0.002357 |
| 58 | rs852069 | G | 0.036 | 0.005907 | -0.00146 | 0.002418 |
| 59 | rs888345 | A | -0.044 | 0.007315 | 0.000747 | 0.002938 |
| 60 | rs895526 | C | 0.044 | 0.007553 | -0.0046 | 0.002972 |
| 61 | rs913588 | A | -0.034 | 0.005683 | 0.000591 | 0.002318 |
| 62 | rs9373571 | A | 0.034 | 0.005926 | -0.00332 | 0.002334 |
| 63 | rs9555810 | G | 0.047 | 0.006492 | -0.01011 | 0.002564 |
| 64 | rs9565073 | C | 0.034 | 0.005881 | -0.00352 | 0.002341 |
| 65 | rs9635759 | A | 0.058 | 0.006362 | -0.00389 | 0.002573 |
| 66 | rs9647570 | G | 0.046 | 0.008427 | -0.00321 | 0.003346 |
| 67 | rs9939609 | A | -0.042 | 0.00572 | 0.001694 | 0.002377 |
| 68 | rs9997604 | C | 0.039 | 0.006511 | -0.00176 | 0.002613 |

AAM: Age at menarche; TG: Triglycerides; SNPs: Single nucleotide polymorphisms.

^a^ Effect size per allele in AAM (in years)

^b^ Standard error of the genetic association of each effect allele with genetically predicted AAM

^c^ Effect size of each allele on genetically-determined TG (mmol/l per allele)

^d^ Standard error of the genetic association of each effect allele with genetically-determined TG

Table S18. Genetic estimates for the association of genetically predicted AAM with genetically-determined alcohol intake frequency.

|  | SNPs | Effect allele | X_k_^a^ | σX_k_^b^ | Z_k_^c^ | σZ_k_^d^ |
| --- | --- | --- | --- | --- | --- | --- |
| 1 | rs10144321 | G | -0.042 | 0.006618 | 0.000421 | 0.003597 |
| 2 | rs10483727 | C | -0.037 | 0.005785 | 0.002372 | 0.003104 |
| 3 | rs1079866 | G | 0.072 | 0.008444 | 0.013176 | 0.004453 |
| 4 | rs10840031 | A | 0.038 | 0.006406 | -0.00759 | 0.003255 |
| 5 | rs10938397 | G | -0.038 | 0.005923 | 0.012806 | 0.003059 |
| 6 | rs11022756 | C | -0.048 | 0.006481 | 0.005266 | 0.003334 |
| 7 | rs11715566 | T | 0.052 | 0.005631 | -0.01011 | 0.003027 |
| 8 | rs11756454 | A | 0.034 | 0.005703 | -0.0034 | 0.003045 |
| 9 | rs11767400 | A | 0.035 | 0.006295 | -0.01397 | 0.003293 |
| 10 | rs12003641 | T | 0.082 | 0.010555 | 0.004844 | 0.005496 |
| 11 | rs12148769 | A | -0.055 | 0.009752 | 0.00897 | 0.00493 |
| 12 | rs12291726 | G | 0.057 | 0.008333 | -0.00047 | 0.004103 |
| 13 | rs12598642 | G | 0.044 | 0.005681 | -0.00622 | 0.003075 |
| 14 | rs12915845 | T | -0.035 | 0.005729 | -0.00268 | 0.003068 |
| 15 | rs13179411 | T | 0.06 | 0.007813 | -0.00835 | 0.004235 |
| 16 | rs13215865 | T | -0.042 | 0.007472 | -0.00465 | 0.003782 |
| 17 | rs1398217 | C | 0.046 | 0.005767 | -0.00896 | 0.003061 |
| 18 | rs1482853 | A | -0.038 | 0.006043 | 0.002138 | 0.003097 |
| 19 | rs1516883 | A | -0.091 | 0.002455 | -0.00225 | 0.003268 |
| 20 | rs1518080 | G | -0.051 | 0.006004 | 0.001641 | 0.003163 |
| 21 | rs1659127 | A | 0.044 | 0.006372 | 0.000542 | 0.003221 |
| 22 | rs16938437 | T | -0.067 | 0.010348 | -0.00595 | 0.005241 |
| 23 | rs17351680 | G | 0.044 | 0.007557 | -0.00134 | 0.004156 |
| 24 | rs1874984 | C | 0.037 | 0.006005 | -0.00464 | 0.003025 |
| 25 | rs2153127 | C | -0.077 | 0.002077 | 0.001322 | 0.003034 |
| 26 | rs2179786 | T | -0.039 | 0.00572 | -0.00206 | 0.003068 |
| 27 | rs2184968 | C | -0.036 | 0.005672 | -0.00603 | 0.00304 |
| 28 | rs2303100 | T | 0.038 | 0.005684 | 0.001857 | 0.003044 |
| 29 | rs2344508 | A | 0.034 | 0.005747 | 0.003095 | 0.003052 |
| 30 | rs2617056 | T | -0.036 | 0.005933 | -0.00154 | 0.003102 |
| 31 | rs2687729 | G | 0.044 | 0.006575 | -0.00356 | 0.00343 |
| 32 | rs2836950 | G | -0.035 | 0.006206 | 0.005736 | 0.003166 |
| 33 | rs2947411 | G | -0.052 | 0.007655 | 0.008165 | 0.003978 |
| 34 | rs3115627 | G | 0.038 | 0.006453 | -0.00389 | 0.003205 |
| 35 | rs3733632 | G | 0.049 | 0.007862 | 0.009734 | 0.004216 |
| 36 | rs3743266 | C | -0.045 | 0.006233 | 0.009664 | 0.003212 |
| 37 | rs3870341 | G | -0.043 | 0.006449 | 0.005346 | 0.003457 |
| 38 | rs3914188 | C | 0.044 | 0.006702 | -0.01616 | 0.003424 |
| 39 | rs4242496 | A | -0.033 | 0.005696 | 0.00145 | 0.003061 |
| 40 | rs4369815 | G | -0.08 | 0.011888 | 0.002703 | 0.006113 |
| 41 | rs466639 | C | 0.075 | 0.008706 | -0.01188 | 0.004591 |
| 42 | rs4801589 | G | 0.032 | 0.005628 | -0.00215 | 0.003034 |
| 43 | rs4840086 | G | -0.036 | 0.00562 | -0.00171 | 0.003055 |
| 44 | rs618678 | T | -0.034 | 0.006148 | -0.00917 | 0.003222 |
| 45 | rs633715 | C | -0.051 | 0.00725 | 0.015628 | 0.003713 |
| 46 | rs6694738 | A | -0.044 | 0.007885 | -0.01442 | 0.004351 |
| 47 | rs6747380 | A | 0.065 | 0.007658 | 0.001162 | 0.004051 |
| 48 | rs6758290 | C | -0.04 | 0.006322 | 0.009138 | 0.003033 |
| 49 | rs6770162 | A | 0.036 | 0.005733 | -0.00371 | 0.003038 |
| 50 | rs6933660 | A | -0.036 | 0.006331 | 0.006975 | 0.003263 |
| 51 | rs7103411 | T | -0.043 | 0.007056 | -0.00305 | 0.003721 |
| 52 | rs7119712 | A | -0.041 | 0.006445 | 0.007824 | 0.003475 |
| 53 | rs740077 | C | -0.046 | 0.007045 | -0.00464 | 0.003746 |
| 54 | rs7642134 | G | 0.038 | 0.005912 | -0.00183 | 0.003121 |
| 55 | rs7821178 | A | -0.045 | 0.006196 | 0.001546 | 0.003202 |
| 56 | rs7853970 | C | -0.037 | 0.00626 | 0.006692 | 0.003071 |
| 57 | rs7944630 | A | 0.047 | 0.005728 | 0.001265 | 0.003074 |
| 58 | rs852069 | G | 0.036 | 0.005907 | 0.000125 | 0.003154 |
| 59 | rs888345 | A | -0.044 | 0.007315 | 0.002315 | 0.003843 |
| 60 | rs895526 | C | 0.044 | 0.007553 | 0.004627 | 0.003872 |
| 61 | rs913588 | A | -0.034 | 0.005683 | 0.006352 | 0.003025 |
| 62 | rs9373571 | A | 0.034 | 0.005926 | -0.00275 | 0.003043 |
| 63 | rs9555810 | G | 0.047 | 0.006492 | -0.0054 | 0.003347 |
| 64 | rs9565073 | C | 0.034 | 0.005881 | -0.00068 | 0.003058 |
| 65 | rs9635759 | A | 0.058 | 0.006362 | 0.00315 | 0.003363 |
| 66 | rs9647570 | G | 0.046 | 0.008427 | -0.00297 | 0.004373 |
| 67 | rs9939609 | A | -0.042 | 0.00572 | 0.018049 | 0.003096 |
| 68 | rs9997604 | C | 0.039 | 0.006511 | -0.00054 | 0.003412 |

AAM: Age at menarche; SNPs: Single nucleotide polymorphisms.

^a^ Effect size per allele in AAM (in years)

^b^ Standard error of the genetic association of each effect allele with genetically predicted AAM

^c^ Effect size of each allele on genetically-determined alcohol intake frequency (times per week/ allele)

^d^ Standard error of the genetic association of each effect allele with genetically-determined alcohol intake frequency

Table S19. Genetic estimates for the association of genetically predicted AAM with genetically-determined sleep duration.

|  | SNPs | Effect allele | X_k_^a^ | σX_k_^b^ | Z_k_^c^ | σZ_k_^d^ |
| --- | --- | --- | --- | --- | --- | --- |
| 1 | rs10144321 | G | -0.042 | 0.006618 | 0.001258 | 0.001899 |
| 2 | rs10483727 | C | -0.037 | 0.005785 | 0.001097 | 0.001639 |
| 3 | rs1079866 | G | 0.072 | 0.008444 | 0.001717 | 0.002352 |
| 4 | rs10840031 | A | 0.038 | 0.006406 | -0.00352 | 0.001719 |
| 5 | rs10938397 | G | -0.038 | 0.005923 | 0.003156 | 0.001616 |
| 6 | rs11022756 | C | -0.048 | 0.006481 | 0.002947 | 0.001761 |
| 7 | rs11715566 | T | 0.052 | 0.005631 | 0.002852 | 0.001599 |
| 8 | rs11756454 | A | 0.034 | 0.005703 | 0.005449 | 0.001608 |
| 9 | rs11767400 | A | 0.035 | 0.006295 | -0.00015 | 0.001739 |
| 10 | rs12003641 | T | 0.082 | 0.010555 | 0.000834 | 0.002903 |
| 11 | rs12148769 | A | -0.055 | 0.009752 | 0.000352 | 0.002603 |
| 12 | rs12291726 | G | 0.057 | 0.008333 | 0.00046 | 0.002167 |
| 13 | rs12598642 | G | 0.044 | 0.005681 | -0.00195 | 0.001624 |
| 14 | rs12915845 | T | -0.035 | 0.005729 | -0.00465 | 0.00162 |
| 15 | rs13179411 | T | 0.06 | 0.007813 | -0.00164 | 0.002236 |
| 16 | rs13215865 | T | -0.042 | 0.007472 | 0.001989 | 0.001997 |
| 17 | rs1398217 | C | 0.046 | 0.005767 | -0.00137 | 0.001617 |
| 18 | rs1482853 | A | -0.038 | 0.006043 | -0.00169 | 0.001636 |
| 19 | rs1516883 | A | -0.091 | 0.002455 | -0.00569 | 0.001726 |
| 20 | rs1518080 | G | -0.051 | 0.006004 | -0.00079 | 0.001671 |
| 21 | rs1659127 | A | 0.044 | 0.006372 | -0.00031 | 0.001701 |
| 22 | rs16938437 | T | -0.067 | 0.010348 | 0.001959 | 0.002767 |
| 23 | rs17351680 | G | 0.044 | 0.007557 | 0.001023 | 0.002195 |
| 24 | rs1874984 | C | 0.037 | 0.006005 | 0.000201 | 0.001598 |
| 25 | rs2153127 | C | -0.077 | 0.002077 | -0.00497 | 0.001602 |
| 26 | rs2179786 | T | -0.039 | 0.00572 | -0.00481 | 0.00162 |
| 27 | rs2184968 | C | -0.036 | 0.005672 | -0.00123 | 0.001605 |
| 28 | rs2303100 | T | 0.038 | 0.005684 | 0.010561 | 0.001607 |
| 29 | rs2344508 | A | 0.034 | 0.005747 | 0.001889 | 0.001612 |
| 30 | rs2617056 | T | -0.036 | 0.005933 | -0.00045 | 0.001638 |
| 31 | rs2687729 | G | 0.044 | 0.006575 | 0.003347 | 0.001812 |
| 32 | rs2836950 | G | -0.035 | 0.006206 | 0.000642 | 0.001672 |
| 33 | rs2947411 | G | -0.052 | 0.007655 | -0.00505 | 0.002102 |
| 34 | rs3115627 | G | 0.038 | 0.006453 | 0.007265 | 0.001692 |
| 35 | rs3733632 | G | 0.049 | 0.007862 | 0.002903 | 0.002226 |
| 36 | rs3743266 | C | -0.045 | 0.006233 | -0.00324 | 0.001696 |
| 37 | rs3870341 | G | -0.043 | 0.006449 | 0.001092 | 0.001826 |
| 38 | rs3914188 | C | 0.044 | 0.006702 | 0.001998 | 0.001808 |
| 39 | rs4242496 | A | -0.033 | 0.005696 | -0.00358 | 0.001616 |
| 40 | rs4369815 | G | -0.08 | 0.011888 | -0.00051 | 0.00323 |
| 41 | rs466639 | C | 0.075 | 0.008706 | 0.000849 | 0.002425 |
| 42 | rs4801589 | G | 0.032 | 0.005628 | 0.002599 | 0.001602 |
| 43 | rs4840086 | G | -0.036 | 0.00562 | -0.00154 | 0.001613 |
| 44 | rs618678 | T | -0.034 | 0.006148 | 0.001568 | 0.001702 |
| 45 | rs633715 | C | -0.051 | 0.00725 | -0.00337 | 0.001961 |
| 46 | rs6694738 | A | -0.044 | 0.007885 | 0.002811 | 0.002298 |
| 47 | rs6747380 | A | 0.065 | 0.007658 | -0.00076 | 0.00214 |
| 48 | rs6758290 | C | -0.04 | 0.006322 | 0.003024 | 0.001602 |
| 49 | rs6770162 | A | 0.036 | 0.005733 | -0.001 | 0.001605 |
| 50 | rs6933660 | A | -0.036 | 0.006331 | 0.000653 | 0.001723 |
| 51 | rs7103411 | T | -0.043 | 0.007056 | -0.00156 | 0.001965 |
| 52 | rs7119712 | A | -0.041 | 0.006445 | 0.001396 | 0.001836 |
| 53 | rs740077 | C | -0.046 | 0.007045 | -0.0026 | 0.001978 |
| 54 | rs7642134 | G | 0.038 | 0.005912 | -0.00245 | 0.001648 |
| 55 | rs7821178 | A | -0.045 | 0.006196 | -0.00376 | 0.001691 |
| 56 | rs7853970 | C | -0.037 | 0.00626 | 0.000524 | 0.001622 |
| 57 | rs7944630 | A | 0.047 | 0.005728 | 0.002612 | 0.001623 |
| 58 | rs852069 | G | 0.036 | 0.005907 | 0.000542 | 0.001666 |
| 59 | rs888345 | A | -0.044 | 0.007315 | -0.00365 | 0.002029 |
| 60 | rs895526 | C | 0.044 | 0.007553 | 0.003406 | 0.002045 |
| 61 | rs913588 | A | -0.034 | 0.005683 | -0.00501 | 0.001598 |
| 62 | rs9373571 | A | 0.034 | 0.005926 | 0.006868 | 0.001607 |
| 63 | rs9555810 | G | 0.047 | 0.006492 | -0.00176 | 0.001767 |
| 64 | rs9565073 | C | 0.034 | 0.005881 | -0.00317 | 0.001614 |
| 65 | rs9635759 | A | 0.058 | 0.006362 | -0.00503 | 0.001776 |
| 66 | rs9647570 | G | 0.046 | 0.008427 | 0.000228 | 0.00231 |
| 67 | rs9939609 | A | -0.042 | 0.00572 | -0.01049 | 0.001635 |
| 68 | rs9997604 | C | 0.039 | 0.006511 | 0.003536 | 0.001802 |

AAM: Age at menarche; SNPs: Single nucleotide polymorphisms.

^a^ Effect size per allele in AAM (in years)

^b^ Standard error of the genetic association of each effect allele with genetically predicted AAM

^c^ Effect size of each allele on genetically-determined sleep duration (hours per allele)

^d^ Standard error of the genetic association of each effect allele with genetically-determined sleep duration

Table S20. Genetic estimates for the association of genetically predicted AAM with genetically-determined WHR.

|  | SNPs | Effect allele | X_k_^a^ | σX_k_^b^ | Z_k_^c^ | σZ_k_^d^ |
| --- | --- | --- | --- | --- | --- | --- |
| 1 | rs10144321 | G | -0.042 | 0.006618 | 0.0013 | 0.006 |
| 2 | rs10483727 | C | -0.037 | 0.005785 | 0.0058 | 0.0045 |
| 3 | rs1079866 | G | 0.072 | 0.008444 | -0.006 | 0.0078 |
| 4 | rs10840031 | A | 0.038 | 0.006406 | -0.013 | 0.0048 |
| 5 | rs10938397 | G | -0.038 | 0.005923 | 0.013 | 0.0045 |
| 6 | rs11022756 | C | -0.048 | 0.006481 | 0.01 | 0.0058 |
| 7 | rs11715566 | T | 0.052 | 0.005631 | -0.0014 | 0.0051 |
| 8 | rs11756454 | A | 0.034 | 0.005703 | -0.0014 | 0.0052 |
| 9 | rs11767400 | A | 0.035 | 0.006295 | 0.0024 | 0.0058 |
| 10 | rs12003641 | T | 0.082 | 0.010555 | 0.0038 | 0.0095 |
| 11 | rs12148769 | A | -0.055 | 0.009752 | -0.0032 | 0.0075 |
| 12 | rs12291726 | G | 0.057 | 0.008333 | 0.0046 | 0.0076 |
| 13 | rs12598642 | G | 0.044 | 0.005681 | -0.0098 | 0.0052 |
| 14 | rs12915845 | T | -0.035 | 0.005729 | 0.0017 | 0.0053 |
| 15 | rs13179411 | T | 0.06 | 0.007813 | -0.0014 | 0.007 |
| 16 | rs13215865 | T | -0.042 | 0.007472 | 0.017 | 0.0066 |
| 17 | rs1398217 | C | 0.046 | 0.005767 | -8.00E-04 | 0.0052 |
| 18 | rs1482853 | A | -0.038 | 0.006043 | -0.019 | 0.0054 |
| 19 | rs1516883 | A | -0.091 | 0.002455 | 6.00E-04 | 0.0056 |
| 20 | rs1518080 | G | -0.051 | 0.006004 | 1.00E-04 | 0.0054 |
| 21 | rs1659127 | A | 0.044 | 0.006372 | 0.0046 | 0.0048 |
| 22 | rs16938437 | T | -0.067 | 0.010348 | -0.0019 | 0.0079 |
| 23 | rs17351680 | G | 0.044 | 0.007557 | -0.0065 | 0.0068 |
| 24 | rs1874984 | C | 0.037 | 0.006005 | -0.0076 | 0.0056 |
| 25 | rs2153127 | C | -0.077 | 0.002077 | 0.001 | 0.0052 |
| 26 | rs2179786 | T | -0.039 | 0.00572 | -0.0037 | 0.0052 |
| 27 | rs2184968 | C | -0.036 | 0.005672 | -0.012 | 0.0052 |
| 28 | rs2303100 | T | 0.038 | 0.005684 | 0.0059 | 0.0052 |
| 29 | rs2344508 | A | 0.034 | 0.005747 | -0.0068 | 0.0044 |
| 30 | rs2617056 | T | -0.036 | 0.005933 | -0.0055 | 0.0053 |
| 31 | rs2687729 | G | 0.044 | 0.006575 | -0.013 | 0.0058 |
| 32 | rs2836950 | G | -0.035 | 0.006206 | 0.0033 | 0.0057 |
| 33 | rs2947411 | G | -0.052 | 0.007655 | 0.015 | 0.0058 |
| 34 | rs3115627 | G | 0.038 | 0.006453 | -0.0078 | 0.0056 |
| 35 | rs3733632 | G | 0.049 | 0.007862 | 0.0022 | 0.0072 |
| 36 | rs3743266 | C | -0.045 | 0.006233 | -0.0016 | 0.0056 |
| 37 | rs3870341 | G | -0.043 | 0.006449 | 0.011 | 0.0059 |
| 38 | rs3914188 | C | 0.044 | 0.006702 | 0.0068 | 0.0061 |
| 39 | rs4242496 | A | -0.033 | 0.005696 | 1.00E-04 | 0.0052 |
| 40 | rs4369815 | G | -0.08 | 0.011888 | -0.0059 | 0.011 |
| 41 | rs466639 | C | 0.075 | 0.008706 | -0.002 | 0.0077 |
| 42 | rs4801589 | G | 0.032 | 0.005628 | -0.0045 | 0.0052 |
| 43 | rs4840086 | G | -0.036 | 0.00562 | 0.0045 | 0.0052 |
| 44 | rs618678 | T | -0.034 | 0.006148 | -0.008 | 0.0056 |
| 45 | rs633715 | C | -0.051 | 0.00725 | 0.02 | 0.0057 |
| 46 | rs6694738 | A | -0.044 | 0.007885 | 0.0043 | 0.0069 |
| 47 | rs6747380 | A | 0.065 | 0.007658 | 0.0074 | 0.0067 |
| 48 | rs6758290 | C | -0.04 | 0.006322 | 0.0097 | 0.0057 |
| 49 | rs6770162 | A | 0.036 | 0.005733 | 0.0052 | 0.0053 |
| 50 | rs6933660 | A | -0.036 | 0.006331 | 0.0057 | 0.0057 |
| 51 | rs7103411 | T | -0.043 | 0.007056 | 0.019 | 0.0053 |
| 52 | rs7119712 | A | -0.041 | 0.006445 | 0.0075 | 0.0059 |
| 53 | rs740077 | C | -0.046 | 0.007045 | -0.0014 | 0.0063 |
| 54 | rs7642134 | G | 0.038 | 0.005912 | 0.0014 | 0.0053 |
| 55 | rs7821178 | A | -0.045 | 0.006196 | 0.0069 | 0.0056 |
| 56 | rs7853970 | C | -0.037 | 0.00626 | -0.0079 | 0.0057 |
| 57 | rs7944630 | A | 0.047 | 0.005728 | 5.00E-04 | 0.0052 |
| 58 | rs852069 | G | 0.036 | 0.005907 | -3.00E-04 | 0.0054 |
| 59 | rs888345 | A | -0.044 | 0.007315 | -0.013 | 0.0066 |
| 60 | rs895526 | C | 0.044 | 0.007553 | 0.0064 | 0.0067 |
| 61 | rs913588 | A | -0.034 | 0.005683 | -0.0068 | 0.0051 |
| 62 | rs9373571 | A | 0.034 | 0.005926 | -0.0034 | 0.0044 |
| 63 | rs9555810 | G | 0.047 | 0.006492 | 0.0026 | 0.0059 |
| 64 | rs9565073 | C | 0.034 | 0.005881 | -0.0016 | 0.0056 |
| 65 | rs9635759 | A | 0.058 | 0.006362 | 0.0078 | 0.0059 |
| 66 | rs9647570 | G | 0.046 | 0.008427 | 0.006 | 0.0077 |
| 67 | rs9939609 | A | -0.042 | 0.00572 | 0.037 | 0.0044 |
| 68 | rs9997604 | C | 0.039 | 0.006511 | 9.00E-04 | 0.006 |

AAM: Age at menarche; WHR: Waist-hip-ratio; SNPs: Single nucleotide polymorphisms.

^a^ Effect size per allele in AAM (in years)

^b^ Standard error of the genetic association of each effect allele with genetically predicted AAM

^c^ Effect size of each allele on genetically-determined WHR (percentage point/ allele)

^d^ Standard error of the genetic association of each effect allele with genetically-determined WHR

Table S21. Genetic estimates for the association of genetically predicted AAM with genetically-determined FBG.

|  | SNPs | Effect allele | X_k_^a^ | σX_k_^b^ | Z_k_^c^ | σZ_k_^d^ |
| --- | --- | --- | --- | --- | --- | --- |
| 1 | rs10144321 | G | -0.042 | 0.006618 | 0.0098 | 0.0042 |
| 2 | rs10483727 | C | -0.037 | 0.005785 | -0.0067 | 0.0038 |
| 3 | rs1079866 | G | 0.072 | 0.008444 | 6.00E-04 | 0.0053 |
| 4 | rs10840031 | A | 0.038 | 0.006406 | -0.0047 | 0.0039 |
| 5 | rs10938397 | G | -0.038 | 0.005923 | -0.0019 | 0.0039 |
| 6 | rs11022756 | C | -0.048 | 0.006481 | -3.00E-04 | 0.0041 |
| 7 | rs11715566 | T | 0.052 | 0.005631 | -0.0045 | 0.0037 |
| 8 | rs11756454 | A | 0.034 | 0.005703 | -0.0062 | 0.0036 |
| 9 | rs11767400 | A | 0.035 | 0.006295 | 0.0046 | 0.004 |
| 10 | rs12003641 | T | 0.082 | 0.010555 | 0.0035 | 0.0066 |
| 11 | rs12148769 | A | -0.055 | 0.009752 | -0.014 | 0.0066 |
| 12 | rs12291726 | G | 0.057 | 0.008333 | 0.0049 | 0.005 |
| 13 | rs12598642 | G | 0.044 | 0.005681 | -0.0039 | 0.0037 |
| 14 | rs12915845 | T | -0.035 | 0.005729 | -0.0021 | 0.0037 |
| 15 | rs13179411 | T | 0.06 | 0.007813 | -2.00E-04 | 0.0048 |
| 16 | rs13215865 | T | -0.042 | 0.007472 | 0.002 | 0.0047 |
| 17 | rs1398217 | C | 0.046 | 0.005767 | 7.00E-04 | 0.0037 |
| 18 | rs1482853 | A | -0.038 | 0.006043 | 0.0038 | 0.004 |
| 19 | rs1516883 | A | -0.091 | 0.002455 | 0.0073 | 0.0039 |
| 20 | rs1518080 | G | -0.051 | 0.006004 | -4.00E-04 | 0.0038 |
| 21 | rs1659127 | A | 0.044 | 0.006372 | -0.0044 | 0.0042 |
| 22 | rs16938437 | T | -0.067 | 0.010348 | -0.0076 | 0.0068 |
| 23 | rs17351680 | G | 0.044 | 0.007557 | -0.0014 | 0.0047 |
| 24 | rs1874984 | C | 0.037 | 0.006005 | -0.0072 | 0.0039 |
| 25 | rs2153127 | C | -0.077 | 0.002077 | -0.0032 | 0.0039 |
| 26 | rs2179786 | T | -0.039 | 0.00572 | -0.0026 | 0.0037 |
| 27 | rs2184968 | C | -0.036 | 0.005672 | 0.0069 | 0.0036 |
| 28 | rs2303100 | T | 0.038 | 0.005684 | -0.001 | 0.0037 |
| 29 | rs2344508 | A | 0.034 | 0.005747 | -0.0077 | 0.0037 |
| 30 | rs2617056 | T | -0.036 | 0.005933 | 0.0073 | 0.0038 |
| 31 | rs2687729 | G | 0.044 | 0.006575 | -0.0071 | 0.0041 |
| 32 | rs2836950 | G | -0.035 | 0.006206 | 0.006 | 0.004 |
| 33 | rs2947411 | G | -0.052 | 0.007655 | 0.0038 | 0.0047 |
| 34 | rs3115627 | G | 0.038 | 0.006453 | -0.0022 | 0.0039 |
| 35 | rs3733632 | G | 0.049 | 0.007862 | 0.0037 | 0.0051 |
| 36 | rs3743266 | C | -0.045 | 0.006233 | 0.0093 | 0.004 |
| 37 | rs3870341 | G | -0.043 | 0.006449 | 0.0024 | 0.0041 |
| 38 | rs3914188 | C | 0.044 | 0.006702 | 2.00E-04 | 0.0043 |
| 39 | rs4242496 | A | -0.033 | 0.005696 | -0.0036 | 0.0037 |
| 40 | rs4369815 | G | -0.08 | 0.011888 | 0.015 | 0.0077 |
| 41 | rs466639 | C | 0.075 | 0.008706 | 0.0018 | 0.0055 |
| 42 | rs4801589 | G | 0.032 | 0.005628 | 0.0045 | 0.0038 |
| 43 | rs4840086 | G | -0.036 | 0.00562 | 6.00E-04 | 0.0036 |
| 44 | rs618678 | T | -0.034 | 0.006148 | 0.0049 | 0.0039 |
| 45 | rs633715 | C | -0.051 | 0.00725 | 0.0028 | 0.0048 |
| 46 | rs6694738 | A | -0.044 | 0.007885 | 0.005 | 0.0048 |
| 47 | rs6747380 | A | 0.065 | 0.007658 | 0.0089 | 0.0048 |
| 48 | rs6758290 | C | -0.04 | 0.006322 | 0.0025 | 0.004 |
| 49 | rs6770162 | A | 0.036 | 0.005733 | 0.0065 | 0.0037 |
| 50 | rs6933660 | A | -0.036 | 0.006331 | 0.0012 | 0.0041 |
| 51 | rs7103411 | T | -0.043 | 0.007056 | 0.0044 | 0.0045 |
| 52 | rs7119712 | A | -0.041 | 0.006445 | 0.0037 | 0.0041 |
| 53 | rs740077 | C | -0.046 | 0.007045 | -0.0019 | 0.0043 |
| 54 | rs7642134 | G | 0.038 | 0.005912 | -0.0025 | 0.0038 |
| 55 | rs7821178 | A | -0.045 | 0.006196 | 0.0022 | 0.0039 |
| 56 | rs7853970 | C | -0.037 | 0.00626 | -0.0042 | 0.0039 |
| 57 | rs7944630 | A | 0.047 | 0.005728 | 0.0029 | 0.0037 |
| 58 | rs852069 | G | 0.036 | 0.005907 | -0.0027 | 0.0038 |
| 59 | rs888345 | A | -0.044 | 0.007315 | -0.0032 | 0.0047 |
| 60 | rs895526 | C | 0.044 | 0.007553 | 0.0038 | 0.0048 |
| 61 | rs913588 | A | -0.034 | 0.005683 | 0.005 | 0.0036 |
| 62 | rs9373571 | A | 0.034 | 0.005926 | -0.0071 | 0.0037 |
| 63 | rs9555810 | G | 0.047 | 0.006492 | -0.0062 | 0.0042 |
| 64 | rs9565073 | C | 0.034 | 0.005881 | 6.00E-04 | 0.004 |
| 65 | rs9635759 | A | 0.058 | 0.006362 | 0.0043 | 0.0042 |
| 66 | rs9647570 | G | 0.046 | 0.008427 | -0.0022 | 0.0055 |
| 67 | rs9939609 | A | -0.042 | 0.00572 | 0.0061 | 0.0037 |
| 68 | rs9997604 | C | 0.039 | 0.006511 | 0.0093 | 0.0041 |

AAM: Age at menarche; FBG: Fasting blood glucose; SNPs: Single nucleotide polymorphisms.

^a^ Effect size per allele in AAM (in years)

^b^ Standard error of the genetic association of each effect allele with genetically predicted AAM

^c^ Effect size of each allele on genetically-determined FBG (mmol/l per allele))

^d^ Standard error of the genetic association of each effect allele with genetically-determined FBG

Table S22. Genetic estimates for the association of genetically predicted AAM with genetically-determined HDL.

|  | SNPs | Effect allele | X_k_^a^ | σX_k_^b^ | Z_k_^c^ | σZ_k_^d^ |
| --- | --- | --- | --- | --- | --- | --- |
| 1 | rs10144321 | G | -0.042 | 0.006618 | -0.00363 | 0.010835 |
| 2 | rs10483727 | C | -0.037 | 0.005785 | 0.002366 | 0.010814 |
| 3 | rs1079866 | G | 0.072 | 0.008444 | -0.00254 | 0.012928 |
| 4 | rs10840031 | A | 0.038 | 0.006406 | 0.011278 | 0.010841 |
| 5 | rs10938397 | G | -0.038 | 0.005923 | 0.001413 | 0.010476 |
| 6 | rs11022756 | C | -0.048 | 0.006481 | -0.01973 | 0.010929 |
| 7 | rs11715566 | T | 0.052 | 0.005631 | 0.002146 | 0.011338 |
| 8 | rs11756454 | A | 0.034 | 0.005703 | -0.00847 | 0.009815 |
| 9 | rs11767400 | A | 0.035 | 0.006295 | 0.005717 | 0.01169 |
| 10 | rs12003641 | T | 0.082 | 0.010555 | -0.00715 | 0.019154 |
| 11 | rs12148769 | A | -0.055 | 0.009752 | 0.004852 | 0.018009 |
| 12 | rs12291726 | G | 0.057 | 0.008333 | 0.025537 | 0.012745 |
| 13 | rs12598642 | G | 0.044 | 0.005681 | 0.011648 | 0.009952 |
| 14 | rs12915845 | T | -0.035 | 0.005729 | 0.005595 | 0.009967 |
| 15 | rs13179411 | T | 0.06 | 0.007813 | 0.011671 | 0.012466 |
| 16 | rs13215865 | T | -0.042 | 0.007472 | -0.00578 | 0.011967 |
| 17 | rs1398217 | C | 0.046 | 0.005767 | 0.002728 | 0.010044 |
| 18 | rs1482853 | A | -0.038 | 0.006043 | 0.027975 | 0.0117 |
| 19 | rs1516883 | A | -0.091 | 0.002455 | -0.0058 | 0.010343 |
| 20 | rs1518080 | G | -0.051 | 0.006004 | -0.0012 | 0.010936 |
| 21 | rs1659127 | A | 0.044 | 0.006372 | 0.007084 | 0.010645 |
| 22 | rs16938437 | T | -0.067 | 0.010348 | 0.014046 | 0.019003 |
| 23 | rs17351680 | G | 0.044 | 0.007557 | 0.005589 | 0.013525 |
| 24 | rs1874984 | C | 0.037 | 0.006005 | -0.01124 | 0.010396 |
| 25 | rs2153127 | C | -0.077 | 0.002077 | 0.010209 | 0.009999 |
| 26 | rs2179786 | T | -0.039 | 0.00572 | 0.001993 | 0.009875 |
| 27 | rs2184968 | C | -0.036 | 0.005672 | 0.004361 | 0.009917 |
| 28 | rs2303100 | T | 0.038 | 0.005684 | -0.0011 | 0.009902 |
| 29 | rs2344508 | A | 0.034 | 0.005747 | 0.012005 | 0.009943 |
| 30 | rs2617056 | T | -0.036 | 0.005933 | 0.00421 | 0.009974 |
| 31 | rs2687729 | G | 0.044 | 0.006575 | 0.034193 | 0.012373 |
| 32 | rs2836950 | G | -0.035 | 0.006206 | -0.01315 | 0.009906 |
| 33 | rs2947411 | G | -0.052 | 0.007655 | -0.02318 | 0.01319 |
| 34 | rs3115627 | G | 0.038 | 0.006453 | -0.0318 | 0.014852 |
| 35 | rs3733632 | G | 0.049 | 0.007862 | 0.002856 | 0.013438 |
| 36 | rs3743266 | C | -0.045 | 0.006233 | -0.00553 | 0.011427 |
| 37 | rs3870341 | G | -0.043 | 0.006449 | -0.01521 | 0.011948 |
| 38 | rs3914188 | C | 0.044 | 0.006702 | -0.00657 | 0.012053 |
| 39 | rs4242496 | A | -0.033 | 0.005696 | 0.020167 | 0.009931 |
| 40 | rs4369815 | G | -0.08 | 0.011888 | -0.04774 | 0.023133 |
| 41 | rs466639 | C | 0.075 | 0.008706 | 0.011467 | 0.014119 |
| 42 | rs4801589 | G | 0.032 | 0.005628 | -0.0029 | 0.009971 |
| 43 | rs4840086 | G | -0.036 | 0.00562 | -0.00181 | 0.010062 |
| 44 | rs618678 | T | -0.034 | 0.006148 | 0.014171 | 0.010433 |
| 45 | rs633715 | C | -0.051 | 0.00725 | -0.01849 | 0.01251 |
| 46 | rs6694738 | A | -0.044 | 0.007885 | -0.00363 | 0.013419 |
| 47 | rs6747380 | A | 0.065 | 0.007658 | 0.010016 | 0.011546 |
| 48 | rs6758290 | C | -0.04 | 0.006322 | -0.00762 | 0.010143 |
| 49 | rs6770162 | A | 0.036 | 0.005733 | 0.004844 | 0.010083 |
| 50 | rs6933660 | A | -0.036 | 0.006331 | -0.00301 | 0.010537 |
| 51 | rs7103411 | T | -0.043 | 0.007056 | -0.01549 | 0.01264 |
| 52 | rs7119712 | A | -0.041 | 0.006445 | -0.00874 | 0.011128 |
| 53 | rs740077 | C | -0.046 | 0.007045 | -0.00051 | 0.01159 |
| 54 | rs7642134 | G | 0.038 | 0.005912 | -0.01493 | 0.010116 |
| 55 | rs7821178 | A | -0.045 | 0.006196 | -0.01279 | 0.010858 |
| 56 | rs7853970 | C | -0.037 | 0.00626 | 0.007862 | 0.010177 |
| 57 | rs7944630 | A | 0.047 | 0.005728 | 0.009528 | 0.010113 |
| 58 | rs852069 | G | 0.036 | 0.005907 | 0.000662 | 0.01023 |
| 59 | rs888345 | A | -0.044 | 0.007315 | 0.012617 | 0.011198 |
| 60 | rs895526 | C | 0.044 | 0.007553 | 0.008882 | 0.015036 |
| 61 | rs913588 | A | -0.034 | 0.005683 | -0.01291 | 0.009809 |
| 62 | rs9373571 | A | 0.034 | 0.005926 | 0.007057 | 0.009913 |
| 63 | rs9555810 | G | 0.047 | 0.006492 | -0.00281 | 0.011337 |
| 64 | rs9565073 | C | 0.034 | 0.005881 | 0.016453 | 0.010241 |
| 65 | rs9635759 | A | 0.058 | 0.006362 | 0.025096 | 0.010472 |
| 66 | rs9647570 | G | 0.046 | 0.008427 | 0.022038 | 0.014112 |
| 67 | rs9939609 | A | -0.042 | 0.00572 | -0.00943 | 0.009934 |
| 68 | rs9997604 | C | 0.039 | 0.006511 | -0.00883 | 0.011265 |

AAM: Age at menarche; HDL: high-density lipoprotein; SNPs: Single nucleotide polymorphisms.

^a^ Effect size per allele in AAM (in years)

^b^ Standard error of the genetic association of each effect allele with genetically predicted AAM

^c^ Effect size of each allele on genetically-determined HDL (mmol/l per allele)

^d^ Standard error of the genetic association of each effect allele with genetically-determined HDL

Table S23. Genetic estimates for the association of genetically predicted AAM with genetically-determined age that started HRT.

|  | SNPs | Effect allele | X_k_^a^ | σX_k_^b^ | Z_k_^c^ | σZ_k_^d^ |
| --- | --- | --- | --- | --- | --- | --- |
| 1 | rs10144321 | G | -0.042 | 0.006618 | 0.001214 | 0.005646 |
| 2 | rs10483727 | C | -0.037 | 0.005785 | -0.01343 | 0.004862 |
| 3 | rs1079866 | G | 0.072 | 0.008444 | 0.004572 | 0.006963 |
| 4 | rs10840031 | A | 0.038 | 0.006406 | 0.001882 | 0.00512 |
| 5 | rs10938397 | G | -0.038 | 0.005923 | 0.000645 | 0.004806 |
| 6 | rs11022756 | C | -0.048 | 0.006481 | -0.00798 | 0.005231 |
| 7 | rs11715566 | T | 0.052 | 0.005631 | -0.0077 | 0.004748 |
| 8 | rs11756454 | A | 0.034 | 0.005703 | -0.00129 | 0.004776 |
| 9 | rs11767400 | A | 0.035 | 0.006295 | -0.00543 | 0.00518 |
| 10 | rs12003641 | T | 0.082 | 0.010555 | 0.014261 | 0.008581 |
| 11 | rs12148769 | A | -0.055 | 0.009752 | -0.0133 | 0.007724 |
| 12 | rs12291726 | G | 0.057 | 0.008333 | 0.013324 | 0.006438 |
| 13 | rs12598642 | G | 0.044 | 0.005681 | -0.003 | 0.004826 |
| 14 | rs12915845 | T | -0.035 | 0.005729 | 0.007579 | 0.004811 |
| 15 | rs13179411 | T | 0.06 | 0.007813 | 0.015425 | 0.006658 |
| 16 | rs13215865 | T | -0.042 | 0.007472 | -0.01063 | 0.005923 |
| 17 | rs1398217 | C | 0.046 | 0.005767 | 0.002898 | 0.004806 |
| 18 | rs1482853 | A | -0.038 | 0.006043 | -0.00893 | 0.004865 |
| 19 | rs1516883 | A | -0.091 | 0.002455 | 0.001067 | 0.005119 |
| 20 | rs1518080 | G | -0.051 | 0.006004 | -0.0097 | 0.004942 |
| 21 | rs1659127 | A | 0.044 | 0.006372 | -0.00057 | 0.005043 |
| 22 | rs16938437 | T | -0.067 | 0.010348 | 0.028112 | 0.008233 |
| 23 | rs17351680 | G | 0.044 | 0.007557 | 0.01231 | 0.006507 |
| 24 | rs1874984 | C | 0.037 | 0.006005 | -0.0029 | 0.004749 |
| 25 | rs2153127 | C | -0.077 | 0.002077 | -0.00738 | 0.00476 |
| 26 | rs2179786 | T | -0.039 | 0.00572 | 0.003704 | 0.004811 |
| 27 | rs2184968 | C | -0.036 | 0.005672 | -0.01345 | 0.004777 |
| 28 | rs2303100 | T | 0.038 | 0.005684 | 0.009931 | 0.004765 |
| 29 | rs2344508 | A | 0.034 | 0.005747 | 0.001994 | 0.004792 |
| 30 | rs2617056 | T | -0.036 | 0.005933 | 0.002716 | 0.004867 |
| 31 | rs2687729 | G | 0.044 | 0.006575 | 0.009244 | 0.005369 |
| 32 | rs2836950 | G | -0.035 | 0.006206 | 0.013467 | 0.004962 |
| 33 | rs2947411 | G | -0.052 | 0.007655 | 0.000248 | 0.006248 |
| 34 | rs3115627 | G | 0.038 | 0.006453 | 0.005002 | 0.005004 |
| 35 | rs3733632 | G | 0.049 | 0.007862 | -0.00285 | 0.006645 |
| 36 | rs3743266 | C | -0.045 | 0.006233 | -0.0067 | 0.005043 |
| 37 | rs3870341 | G | -0.043 | 0.006449 | -0.00141 | 0.005402 |
| 38 | rs3914188 | C | 0.044 | 0.006702 | 0.007573 | 0.005371 |
| 39 | rs4242496 | A | -0.033 | 0.005696 | -0.00383 | 0.004811 |
| 40 | rs4369815 | G | -0.08 | 0.011888 | 0.009636 | 0.009512 |
| 41 | rs466639 | C | 0.075 | 0.008706 | 0.005154 | 0.007208 |
| 42 | rs4801589 | G | 0.032 | 0.005628 | -0.00159 | 0.004749 |
| 43 | rs4840086 | G | -0.036 | 0.00562 | 0.002784 | 0.004783 |
| 44 | rs618678 | T | -0.034 | 0.006148 | -0.00201 | 0.005057 |
| 45 | rs633715 | C | -0.051 | 0.00725 | -0.00774 | 0.005824 |
| 46 | rs6694738 | A | -0.044 | 0.007885 | 0.003537 | 0.006862 |
| 47 | rs6747380 | A | 0.065 | 0.007658 | 0.007066 | 0.006349 |
| 48 | rs6758290 | C | -0.04 | 0.006322 | 0.00759 | 0.004753 |
| 49 | rs6770162 | A | 0.036 | 0.005733 | 0.007469 | 0.004763 |
| 50 | rs6933660 | A | -0.036 | 0.006331 | -0.00868 | 0.005114 |
| 51 | rs7103411 | T | -0.043 | 0.007056 | 0.003662 | 0.005828 |
| 52 | rs7119712 | A | -0.041 | 0.006445 | -0.00053 | 0.005455 |
| 53 | rs740077 | C | -0.046 | 0.007045 | -0.00566 | 0.005894 |
| 54 | rs7642134 | G | 0.038 | 0.005912 | 0.009112 | 0.004899 |
| 55 | rs7821178 | A | -0.045 | 0.006196 | -0.00394 | 0.005015 |
| 56 | rs7853970 | C | -0.037 | 0.00626 | -0.00848 | 0.004815 |
| 57 | rs7944630 | A | 0.047 | 0.005728 | -0.00049 | 0.004819 |
| 58 | rs852069 | G | 0.036 | 0.005907 | 0.001682 | 0.004943 |
| 59 | rs888345 | A | -0.044 | 0.007315 | -0.00744 | 0.006041 |
| 60 | rs895526 | C | 0.044 | 0.007553 | -0.00069 | 0.006067 |
| 61 | rs913588 | A | -0.034 | 0.005683 | 0.000423 | 0.004743 |
| 62 | rs9373571 | A | 0.034 | 0.005926 | 0.007432 | 0.004766 |
| 63 | rs9555810 | G | 0.047 | 0.006492 | 0.014442 | 0.005257 |
| 64 | rs9565073 | C | 0.034 | 0.005881 | -0.01103 | 0.004785 |
| 65 | rs9635759 | A | 0.058 | 0.006362 | -0.00115 | 0.005274 |
| 66 | rs9647570 | G | 0.046 | 0.008427 | 0.011441 | 0.006814 |
| 67 | rs9939609 | A | -0.042 | 0.00572 | 0.010907 | 0.004864 |
| 68 | rs9997604 | C | 0.039 | 0.006511 | -0.00483 | 0.005348 |

AAM: Age at menarche; HRT: Hormone-replacement therapy; SNPs: Single nucleotide polymorphisms.

^a^ Effect size per allele in AAM (in years)

^b^ Standard error of the genetic association of each effect allele with genetically predicted AAM

^c^ Effect size of each allele on genetically-determined age that started HRT (years/allele)

^d^ Standard error of the genetic association of each effect allele with genetically-determined age that started HR

Table S24. MR estimates of associations of genetically predicted AAM with each genetically-determined risk factor.

|  | MR Method | OR | 95% CI | p-value |
| --- | --- | --- | --- | --- |
| Current smoking behavior | Simple Median | 0.99 | 0.98-0.99 | 0.078 |
|  | Weighted Median | 0.98 | 0.97-0.99 | 0.007 |
|  | MR Egger | 0.95 | 0.91-0.99 | 0.066 |
|  | Inverse variance weighted | 0.99 | 0.97-0.99 | 0.049 |
| HbA1c | Simple Median | 0.86 | 0.74-1.01 | 0.059 |
|  | Weighted Median | 1.01 | 0.85-1.19 | 0.950 |
|  | MR Egger | 1.34 | 0.62-2.91 | 0.460 |
|  | Inverse variance weighted | 0.75 | 0.61-0.92 | 0.005 |
| SBP | Simple Median | 0.97 | 0.94-0.99 | 0.015 |
|  | Weighted Median | 0.97 | 0.95-1.00 | 0.063 |
|  | MR Egger | 1.05 | 0.95-1.16 | 0.345 |
|  | Inverse variance weighted | 0.97 | 0.95-0.99 | 0.048 |
| TG | Simple Median | 0.97 | 0.95-0.99 | 0.017 |
|  | Weighted Median | 0.97 | 0.94-0.99 | 0.006 |
|  | MR Egger | 0.90 | 0.80-1.02 | 0.102 |
|  | Inverse variance weighted | 0.97 | 0.94-0.99 | 0.041 |
| alcohol intake frequency | Simple Median | 0.95 | 0.93-0.98 | 0 |
|  | Weighted Median | 0.97 | 0.94-1.00 | 0.026 |
|  | MR Egger | 1.04 | 0.92-1.18 | 0.504 |
|  | Inverse variance weighted | 0.95 | 0.92-0.98 | 0.001 |
| sleep duration | Simple Median | 1.01 | 1.00-1.03 | 0.127 |
|  | Weighted Median | 1.02 | 1.01-1.04 | 0.013 |
|  | MR Egger | 1.01 | 0.94-1.08 | 0.734 |
|  | Inverse variance weighted | 1.03 | 1.01-1.05 | 0.001 |
| WHR | Simple Median | 0.99 | 0.94-1.03 | 0.538 |
|  | Weighted Median | 0.99 | 0.94-1.04 | 0.613 |
|  | MR Egger | 1.03 | 0.84-1.25 | 0.800 |
|  | Inverse variance weighted | 0.95 | 0.90-0.99 | 0.035 |
| FBG | Simple Median | 0.96 | 0.93-1.00 | 0.026 |
|  | Weighted Median | 0.97 | 0.93-1.01 | 0.108 |
|  | MR Egger | 1.04 | 0.94-1.15 | 0.445 |
|  | Inverse variance weighted | 0.97 | 0.95-0.99 | 0.048 |
| HDL | Simple Median | 1.09 | 0.99-1.19 | 0.066 |
|  | Weighted Median | 1.08 | 0.98-1.18 | 0.113 |
|  | MR Egger | 1.22 | 0.97-1.55 | 0.091 |
|  | Inverse variance weighted | 1.08 | 1.02-1.15 | 0.011 |
| Age that started HRT | Simple Median | 1.06 | 1.02-1.10 | 0.003 |
|  | Weighted Median | 1.06 | 1.03-1.10 | 0.001 |
|  | MR Egger | 1.06 | 0.92-1.23 | 0.411 |
|  | Inverse variance weighted | 1.05 | 1.01-1.09 | 0.008 |

MR: Mendelian randomization; AAM: Age at menarche; OR: Odds ratio; CI: Confidence interval; HbA1c: Hemoglobin A1c; SBP: Systolic blood pressure; TG: Triglycerides; WHR: Waist-hip-ratio; HDL: High-density lipoprotein; HRT: Hormone-replacement therapy.

Table S25. Genetic estimates for the association of genetically-determined current smoking behavior with genetically predicted MI after adjusting for genetically predicted AAM.

|  | SNPs | Effect allele | Z_k_^a^ | σZ_k_^b^ | Y_k_ ^c^ | σY_k_ ^d^ | X_k_^e^ | σX_k_^f^ |
| --- | --- | --- | --- | --- | --- | --- | --- | --- |
| 1 | rs10891481 | G | 0.008446 | 0.001363 | -9.32E-05 | 0.010418 | -0.0011 | 0.005448 |
| 2 | rs11096777 | C | 0.009903 | 0.001713 | 0.0031645 | 0.013598 | 3.00E-04 | 0.007977 |
| 3 | rs11210887 | A | -0.00955 | 0.001441 | -0.020575 | 0.010868 | 0.028 | 0.0063 |
| 4 | rs113382419 | A | 0.021268 | 0.002099 | 0.0165489 | 0.020816 | 0.027 | 0.013147 |
| 5 | rs17730481 | A | 0.008563 | 0.001422 | 0.0035701 | 0.01142 | 0.0048 | 0.006354 |
| 6 | rs3773814 | C | 0.010087 | 0.001838 | 0.0334613 | 0.014526 | -0.0025 | 0.007533 |
| 7 | rs62474713 | A | -0.00868 | 0.001324 | -0.006412 | 0.010216 | -0.003 | 0.005568 |
| 8 | rs73227362 | C | -0.00982 | 0.001665 | -0.018759 | 0.013009 | 0.0015 | 0.00743 |
| 9 | rs7726560 | T | 0.007457 | 0.001333 | 0.0018321 | 0.010475 | -4.00E-04 | 0.005314 |
| 10 | rs7807019 | G | 0.007598 | 0.001327 | 0.0210339 | 0.010215 | 0.0089 | 0.005724 |
| 11 | rs8033799 | C | 0.008913 | 0.001622 | -0.008355 | 0.013341 | -0.014 | 0.006918 |

MI: Myocardial infarction; AAM: Age at menarche; SNPs: Single nucleotide polymorphisms.

^a^ Effect size of each allele on genetically-determined current smoking behavior (pack-years per allele)

^b^ Standard error of the genetic association of each effect allele with genetically-determined current smoking behavior

^c^ Effect size per allele in the log-odds or the log probability of MI after adjusting for genetically predicted AAM

^d^ Standard error of the genetic association of each effect allele with genetically predicted MI after adjusting for genetically predicted AAM

^e^ Effect size per allele in AAM (in years)

^f^ Standard error of the genetic association of each effect allele with genetically predicted AAM

Table S26. Genetic estimates for the association of genetically-determined HbA1c with genetically predicted MI after adjusting for genetically predicted AAM.

|  | SNPs | Effect allele | Z_k_^a^ | σZ_k_^b^ | Y_k_ ^c^ | σY_k_ ^d^ | X_k_^e^ | σX_k_^f^ |
| --- | --- | --- | --- | --- | --- | --- | --- | --- |
| 1 | rs1004558 | T | 0.44709 | 0.020003 | 0.010855 | 0.0137 | 0.0046 | 0.008098 |
| 2 | rs1044145 | C | 0.085473 | 0.015431 | -0.00946 | 0.010435 | 0.0066 | 0.005859 |
| 3 | rs10487796 | A | -0.16103 | 0.015411 | -0.00675 | 0.010297 | 0.0033 | 0.005662 |
| 4 | rs10733564 | G | -0.12746 | 0.022684 | -0.04637 | 0.016097 | 0.01 | 0.00985 |
| 5 | rs10758658 | A | -0.13066 | 0.019186 | -0.01796 | 0.013112 | -0.012 | 0.007508 |
| 6 | rs10784889 | T | -0.08716 | 0.015611 | -0.00292 | 0.010553 | 0.0041 | 0.005675 |
| 7 | rs10811660 | A | -0.30869 | 0.020265 | -0.002 | 0.013723 | 0.0084 | 0.008105 |
| 8 | rs10830963 | G | 0.29735 | 0.017141 | 0.022073 | 0.011736 | 0.012 | 0.007252 |
| 9 | rs10842987 | C | -0.1624 | 0.019136 | 0.005159 | 0.013354 | 0.0047 | 0.007134 |
| 10 | rs10873398 | A | -0.09381 | 0.016088 | -0.01834 | 0.01101 | 0.0014 | 0.006153 |
| 11 | rs11039165 | G | -0.13326 | 0.017253 | 0.015414 | 0.012448 | -0.0089 | 0.006486 |
| 12 | rs11065979 | T | -0.10915 | 0.015494 | 0.072748 | 0.01193 | -0.0066 | 0.005983 |
| 13 | rs11204535 | G | -0.10312 | 0.017401 | 0.026661 | 0.011664 | 0.0024 | 0.008209 |
| 14 | rs1121980 | A | 0.15492 | 0.015526 | 0.022913 | 0.010506 | 0.041 | 0.005868 |
| 15 | rs11257655 | T | 0.21882 | 0.018898 | 0.021494 | 0.012199 | 0.0036 | 0.00726 |
| 16 | rs11580608 | T | 0.12292 | 0.016481 | 0.016919 | 0.011414 | -0.0027 | 0.006138 |
| 17 | rs11602873 | T | -0.18717 | 0.021037 | -0.02511 | 0.014153 | 0.0017 | 0.007918 |
| 18 | rs11708067 | G | -0.26157 | 0.01782 | -0.00718 | 0.012893 | -0.0047 | 0.007134 |
| 19 | rs1175550 | G | -0.17719 | 0.01848 | -0.01056 | 0.013118 | -0.013 | 0.007326 |
| 20 | rs117721418 | T | -0.1915 | 0.028111 | -0.01715 | 0.021988 | -0.014 | 0.010682 |
| 21 | rs1215468 | G | -0.13265 | 0.016939 | -0.00862 | 0.011851 | 0.0037 | 0.006348 |
| 22 | rs12210538 | G | -0.12788 | 0.018075 | -0.00353 | 0.013675 | 0.032 | 0.011088 |
| 23 | rs12365580 | A | -0.12988 | 0.021566 | -0.03285 | 0.014002 | 0.0023 | 0.008234 |
| 24 | rs12448999 | A | -0.08609 | 0.01556 | 0.000906 | 0.010328 | 0.01 | 0.005655 |
| 25 | rs12451511 | G | 0.10704 | 0.019364 | 0.033819 | 0.01235 | 0.0054 | 0.007474 |
| 26 | rs12600858 | A | -0.1311 | 0.01843 | -0.01144 | 0.011564 | -0.0024 | 0.006695 |
| 27 | rs12633493 | T | 0.11715 | 0.015626 | 0.017504 | 0.010503 | 5.00E-04 | 0.005692 |
| 28 | rs12668254 | G | -0.20599 | 0.028435 | 0.015258 | 0.019054 | -0.013 | 0.01083 |
| 29 | rs12910361 | G | 0.1614 | 0.016961 | 0.016738 | 0.010837 | 0.019 | 0.00638 |
| 30 | rs12912009 | A | -0.14568 | 0.017227 | -0.00429 | 0.011712 | -0.0085 | 0.006486 |
| 31 | rs12984096 | C | 0.21315 | 0.015735 | -0.00755 | 0.010706 | 0.0081 | 0.00632 |
| 32 | rs13019832 | A | -0.12765 | 0.015588 | -0.01056 | 0.01104 | -0.013 | 0.005718 |
| 33 | rs13042148 | T | 0.11857 | 0.021217 | 0.005818 | 0.015932 | 6.00E-04 | 0.007971 |
| 34 | rs147208676 | T | 0.15993 | 0.028231 | -0.0096 | 0.020457 | -0.012 | 0.009997 |
| 35 | rs1496653 | G | -0.13138 | 0.019011 | 0.005735 | 0.01254 | 1.00E-04 | 0.007979 |
| 36 | rs1550026 | T | 0.10777 | 0.015667 | 0.000626 | 0.010979 | 0.0045 | 0.005702 |
| 37 | rs1574285 | T | -0.09232 | 0.01569 | 0.009377 | 0.010494 | 0.0075 | 0.005852 |
| 38 | rs1641523 | T | -0.12694 | 0.015571 | -0.00533 | 0.010974 | 0.0038 | 0.006201 |
| 39 | rs17168486 | T | 0.16157 | 0.020347 | 0.00717 | 0.012723 | -0.012 | 0.007508 |
| 40 | rs174549 | A | -0.10537 | 0.016576 | -0.02401 | 0.011688 | 5.00E-04 | 0.006643 |
| 41 | rs1787663 | A | 0.10095 | 0.016889 | -0.01311 | 0.012145 | -0.0015 | 0.006592 |
| 42 | rs1800562 | A | -0.5108 | 0.028482 | -0.04874 | 0.026825 | -5.00E-04 | 0.013295 |
| 43 | rs181204 | G | 0.11418 | 0.016399 | 0.018162 | 0.012227 | 0.0047 | 0.005828 |
| 44 | rs1905505 | A | -0.19336 | 0.017013 | -0.02975 | 0.011266 | 0.0052 | 0.006311 |
| 45 | rs1929915 | A | 0.14594 | 0.018823 | -0.03006 | 0.012682 | 0.014 | 0.007356 |
| 46 | rs198325 | T | -0.12324 | 0.01847 | 0.020015 | 0.0137 | -0.0051 | 0.006903 |
| 47 | rs1995138 | A | 0.17294 | 0.028937 | 0.033269 | 0.019955 | -0.0046 | 0.011153 |
| 48 | rs201886961 | T | -0.11206 | 0.01727 | 0.016909 | 0.01117 | -0.0078 | 0.006222 |
| 49 | rs229587 | T | 0.12193 | 0.016377 | 0.006077 | 0.010916 | -0.0015 | 0.006239 |
| 50 | rs2305196 | A | -0.15241 | 0.017432 | 0.010936 | 0.011669 | -0.0045 | 0.006672 |
| 51 | rs2487569 | T | 0.15133 | 0.024673 | 0.00647 | 0.019166 | -0.0021 | 0.009229 |
| 52 | rs2737263 | T | -0.11708 | 0.01701 | 0.005037 | 0.011656 | -0.0015 | 0.006592 |
| 53 | rs2796441 | A | -0.09636 | 0.015513 | -0.01063 | 0.010769 | 0.018 | 0.006219 |
| 54 | rs28504375 | A | -0.23087 | 0.016693 | 0.010487 | 0.011768 | -0.003 | 0.006414 |
| 55 | rs2925979 | C | -0.11956 | 0.016725 | -0.01205 | 0.011783 | 0.0057 | 0.006227 |
| 56 | rs2943640 | C | 0.1491 | 0.016089 | 0.041048 | 0.011261 | 0.0044 | 0.005955 |
| 57 | rs2954021 | G | 0.095504 | 0.015352 | -0.04696 | 0.010153 | -2.00E-04 | 0.005318 |
| 58 | rs3020069 | A | 0.093396 | 0.01649 | -0.01247 | 0.010945 | 0.0012 | 0.006345 |
| 59 | rs33978622 | C | -0.11579 | 0.016391 | 0.00058 | 0.01206 | -0.0073 | 0.006346 |
| 60 | rs340882 | G | 0.18618 | 0.015886 | -0.00296 | 0.010429 | 0.011 | 0.005709 |
| 61 | rs34872471 | C | 0.47526 | 0.016882 | 0.024941 | 0.011353 | -0.009 | 0.006405 |
| 62 | rs368865 | G | 0.1045 | 0.017151 | -0.01961 | 0.012584 | 0.0058 | 0.006747 |
| 63 | rs376563 | C | 0.096419 | 0.01539 | 0.030295 | 0.010203 | 0.0068 | 0.005665 |
| 64 | rs3927482 | G | 0.12184 | 0.020197 | 0.007952 | 0.016458 | -0.013 | 0.009252 |
| 65 | rs415895 | G | -0.13765 | 0.016036 | 0.033314 | 0.010754 | -0.0092 | 0.005917 |
| 66 | rs45504994 | G | -0.09728 | 0.015389 | -0.027 | 0.01023 | -0.0094 | 0.005681 |
| 67 | rs4611812 | T | -0.0987 | 0.015682 | 0.009565 | 0.01068 | -0.0047 | 0.006087 |
| 68 | rs4689394 | G | 0.14488 | 0.015673 | 0.001311 | 0.010998 | 0.0074 | 0.005774 |
| 69 | rs4698874 | C | -0.10502 | 0.015359 | 0.003994 | 0.010085 | 0.0037 | 0.005751 |
| 70 | rs4745982 | G | -1.0375 | 0.029209 | -0.04505 | 0.025543 | -0.03 | 0.016498 |
| 71 | rs4809556 | A | -0.08715 | 0.015637 | -0.00969 | 0.011792 | -0.0067 | 0.006074 |
| 72 | rs4889490 | T | -0.09002 | 0.015729 | 0.000858 | 0.011696 | -0.0075 | 0.005983 |
| 73 | rs4905989 | A | -0.14001 | 0.020779 | -0.01599 | 0.012767 | 0.025 | 0.008424 |
| 74 | rs4972439 | C | -0.14035 | 0.018551 | 0.015943 | 0.012356 | -0.0054 | 0.006993 |
| 75 | rs555895 | G | 0.10458 | 0.016063 | -0.00191 | 0.010944 | 0.0063 | 0.006079 |
| 76 | rs55966194 | G | -0.13601 | 0.017023 | -0.00998 | 0.011996 | -0.004 | 0.006863 |
| 77 | rs560887 | C | 0.40038 | 0.016716 | -0.0051 | 0.012225 | -0.014 | 0.006246 |
| 78 | rs56397034 | C | -0.11569 | 0.015683 | -0.02313 | 0.011139 | -0.021 | 0.00621 |
| 79 | rs594398 | C | 0.086064 | 0.015375 | 0.031198 | 0.010363 | -0.021 | 0.005718 |
| 80 | rs60238952 | G | -0.10404 | 0.018626 | -0.0377 | 0.011781 | 0.0082 | 0.007434 |
| 81 | rs61750929 | T | -0.43693 | 0.033677 | -0.02645 | 0.024136 | -0.014 | 0.012959 |
| 82 | rs61850681 | A | -0.16614 | 0.018084 | -0.0158 | 0.013305 | -0.011 | 0.007075 |
| 83 | rs6602900 | G | -0.16892 | 0.018688 | -0.03959 | 0.017037 | 0.004 | 0.007228 |
| 84 | rs66593272 | T | -0.54055 | 0.040328 | 0.025364 | 0.029517 | 0.012 | 0.017791 |
| 85 | rs67131976 | T | 0.28395 | 0.020297 | 0.013158 | 0.012656 | -0.015 | 0.007653 |
| 86 | rs6726007 | T | -0.10831 | 0.015378 | 0.011612 | 0.010614 | 0.0062 | 0.005739 |
| 87 | rs6784925 | T | -0.0934 | 0.015409 | 0.002767 | 0.010283 | -0.0094 | 0.005715 |
| 88 | rs6785881 | T | -0.10646 | 0.015374 | -0.00584 | 0.010353 | -0.0015 | 0.005633 |
| 89 | rs6878122 | A | -0.13225 | 0.016459 | -0.0212 | 0.012493 | -0.0021 | 0.006874 |
| 90 | rs6920313 | C | 0.095312 | 0.016651 | 0.004249 | 0.010788 | -0.0032 | 0.006102 |
| 91 | rs6953344 | G | 0.10983 | 0.017821 | 0.008858 | 0.011559 | -0.01 | 0.006605 |
| 92 | rs7077479 | A | 0.10373 | 0.016555 | -0.00394 | 0.01103 | 0.0012 | 0.005944 |
| 93 | rs7105853 | A | 0.13366 | 0.023019 | 0.00454 | 0.017195 | 0.014 | 0.009486 |
| 94 | rs7124355 | G | -0.1 | 0.016451 | -0.01545 | 0.01089 | 5.00E-04 | 0.006643 |
| 95 | rs7151822 | C | -0.15893 | 0.017827 | -0.00488 | 0.011241 | -0.016 | 0.006442 |
| 96 | rs7196917 | G | 0.095553 | 0.015504 | 0.01486 | 0.010277 | 0.029 | 0.005901 |
| 97 | rs7206953 | C | -0.1082 | 0.017398 | -0.00188 | 0.011732 | 0.01 | 0.006432 |
| 98 | rs72781658 | G | 0.20186 | 0.022514 | 0.021922 | 0.015396 | -0.0047 | 0.008723 |
| 99 | rs72802358 | C | -0.15063 | 0.025538 | -0.04437 | 0.017895 | -0.0056 | 0.008917 |
| 100 | rs7513688 | A | 0.10972 | 0.016014 | -0.00115 | 0.01079 | 0.014 | 0.006246 |
| 101 | rs7528296 | C | -0.09937 | 0.015818 | -0.0011 | 0.010716 | 0.0059 | 0.005812 |
| 102 | rs75372982 | G | -0.10827 | 0.017165 | -0.01184 | 0.011265 | -0.0041 | 0.006529 |
| 103 | rs76702117 | G | 0.2271 | 0.039114 | 0.009499 | 0.026201 | -0.0067 | 0.014326 |
| 104 | rs7720275 | C | 0.12178 | 0.02043 | 0.000342 | 0.0142 | -0.018 | 0.008244 |
| 105 | rs780093 | C | 0.15748 | 0.015777 | 0.002801 | 0.010608 | 0.017 | 0.005938 |
| 106 | rs78588343 | A | -0.1647 | 0.020022 | -0.02175 | 0.015066 | 0.014 | 0.007782 |
| 107 | rs7898054 | T | -0.18945 | 0.015808 | -0.0187 | 0.010595 | -0.013 | 0.005991 |
| 108 | rs8000868 | T | 0.16963 | 0.020974 | 0.012937 | 0.015122 | 0.0011 | 0.007953 |
| 109 | rs8002606 | G | 0.17541 | 0.020599 | 0.009351 | 0.014098 | -0.0012 | 0.007949 |
| 110 | rs8068844 | C | 0.097508 | 0.016183 | 0.044662 | 0.010687 | -0.016 | 0.006152 |
| 111 | rs838717 | A | 0.10064 | 0.0155 | -0.01014 | 0.010217 | 0.0049 | 0.0057 |
| 112 | rs855791 | G | -0.25462 | 0.015509 | -0.01081 | 0.010579 | 0.0029 | 0.005849 |
| 113 | rs857721 | A | 0.37763 | 0.017303 | -0.01473 | 0.011711 | 0.0026 | 0.006748 |
| 114 | rs9376091 | T | -0.17628 | 0.01755 | -0.01829 | 0.012377 | -0.0044 | 0.006374 |
| 115 | rs9701805 | C | -0.22065 | 0.026826 | -0.04789 | 0.018521 | -0.0014 | 0.010123 |
| 116 | rs9900803 | T | -0.16892 | 0.016657 | -0.03279 | 0.010984 | 0.0032 | 0.006102 |
| 117 | rs9901806 | T | -0.08609 | 0.01568 | 0.01983 | 0.010824 | 0.0019 | 0.005963 |
| 118 | rs998584 | A | 0.12424 | 0.015384 | 0.047772 | 0.011036 | 0.0012 | 0.007332 |

HbA1c: Hemoglobin A1c; MI: Myocardial infarction; AAM: Age at menarche; SNPs: Single nucleotide polymorphisms.

^a^ Effect size of each allele on genetically-determined HbA1c (percentage point per allele)

^b^ Standard error of the genetic association of each effect allele with genetically-determined HbA1c

^c^ Effect size per allele in the log-odds or the log probability of MI after adjusting for genetically predicted AAM

^d^ Standard error of the genetic association of each effect allele with genetically predicted MI after adjusting for genetically predicted AAM

^e^ Effect size per allele in AAM (in years)

^f^ Standard error of the genetic association of each effect allele with genetically predicted AAM

Table S27. Genetic estimates for the association of genetically-determined SBP with genetically predicted MI after adjusting for genetically predicted AAM.

|  | SNPs | Effect allele | Z_k_^a^ | σZ_k_^b^ | Y_k_ ^c^ | σY_k_ ^d^ | X_k_^e^ | σX_k_^f^ |
| --- | --- | --- | --- | --- | --- | --- | --- | --- |
| 1 | rs1000423 | T | 0.017808 | 0.002788 | 0.020222 | 0.011662 | -5.00E-04 | 0.007974 |
| 2 | rs10029530 | T | -0.01903 | 0.002597 | -0.03797 | 0.010547 | -0.0036 | 0.006177 |
| 3 | rs10048404 | T | -0.01493 | 0.002547 | -0.00679 | 0.011059 | 0.0056 | 0.007096 |
| 4 | rs10269774 | A | -0.02078 | 0.002631 | -0.01531 | 0.011469 | -0.0096 | 0.006007 |
| 5 | rs10409243 | T | -0.01536 | 0.002544 | -0.01297 | 0.011004 | 0.0032 | 0.005939 |
| 6 | rs1047891 | A | -0.01612 | 0.002646 | -0.01106 | 0.011567 | 0.0016 | 0.00846 |
| 7 | rs10769253 | A | -0.02394 | 0.003231 | 0.010299 | 0.012985 | -0.023 | 0.007638 |
| 8 | rs10817007 | G | 0.022057 | 0.00368 | 0.031747 | 0.014635 | -0.016 | 0.014505 |
| 9 | rs10853912 | T | 0.015436 | 0.00252 | 0.01658 | 0.01041 | -0.0022 | 0.005916 |
| 10 | rs10857147 | T | 0.037123 | 0.002713 | 0.055613 | 0.012145 | -0.014 | 0.007699 |
| 11 | rs10883543 | T | 0.031002 | 0.003921 | -0.01934 | 0.018306 | 7.00E-04 | 0.0093 |
| 12 | rs10883948 | T | -0.01354 | 0.00246 | 0.02497 | 0.010288 | 0.0063 | 0.005593 |
| 13 | rs11070245 | G | 0.016523 | 0.002471 | -0.0075 | 0.010316 | -0.006 | 0.005789 |
| 14 | rs11191580 | C | -0.0511 | 0.004608 | -0.07286 | 0.015849 | -0.01 | 0.00985 |
| 15 | rs11214436 | T | -0.0155 | 0.002538 | 0.000329 | 0.01046 | -0.0018 | 0.005892 |
| 16 | rs11629120 | C | 0.013985 | 0.002508 | -0.02032 | 0.010888 | 0.0082 | 0.005836 |
| 17 | rs11874246 | T | 0.016884 | 0.0027 | 0.017607 | 0.010925 | 0.0039 | 0.006364 |
| 18 | rs12258967 | G | -0.03144 | 0.002693 | -0.02937 | 0.012205 | 0.0077 | 0.006278 |
| 19 | rs1250259 | A | -0.01885 | 0.002795 | -0.03203 | 0.011472 | -0.0092 | 0.006705 |
| 20 | rs12656497 | C | 0.031728 | 0.002508 | 0.005251 | 0.010358 | -0.007 | 0.005707 |
| 21 | rs12694861 | A | 0.013713 | 0.002511 | -0.01718 | 0.010496 | 0.0018 | 0.005892 |
| 22 | rs12714414 | C | -0.01966 | 0.00356 | -0.02139 | 0.013882 | -0.05 | 0.007749 |
| 23 | rs12967060 | T | 0.016679 | 0.002789 | -0.00894 | 0.012036 | 0.0047 | 0.006968 |
| 24 | rs13016772 | T | 0.016147 | 0.002892 | 0.010487 | 0.011436 | 0.0066 | 0.006775 |
| 25 | rs13154549 | G | 0.030453 | 0.004909 | 0.02766 | 0.020666 | -0.033 | 0.011267 |
| 26 | rs13436194 | G | -0.01855 | 0.002486 | -0.02872 | 0.010244 | -0.01 | 0.005712 |
| 27 | rs138110118 | G | -0.0239 | 0.003908 | 0.02855 | 0.022275 | -0.0024 | 0.008592 |
| 28 | rs1438898 | C | 0.015766 | 0.002848 | -0.0186 | 0.012141 | 0.0076 | 0.006607 |
| 29 | rs144356415 | G | 0.036359 | 0.00629 | -0.06472 | 0.025153 | 0.0099 | 0.012821 |
| 30 | rs1543270 | T | -0.01586 | 0.002475 | 0.013875 | 0.010423 | -0.0087 | 0.005746 |
| 31 | rs1644318 | C | 0.019146 | 0.002539 | 0.015745 | 0.010606 | -0.0021 | 0.005858 |
| 32 | rs167479 | T | -0.02702 | 0.002461 | -0.01281 | 0.015817 | 0.0078 | 0.013383 |
| 33 | rs17056301 | C | 0.018768 | 0.002833 | 0.01665 | 0.011163 | 0.007 | 0.006616 |
| 34 | rs17173238 | G | 0.015442 | 0.002721 | 0.00912 | 0.012513 | 0.0056 | 0.007929 |
| 35 | rs17535443 | A | -0.02169 | 0.002754 | -0.0307 | 0.012275 | -0.017 | 0.006347 |
| 36 | rs17677603 | G | 0.017989 | 0.002528 | 0.023991 | 0.01062 | -0.014 | 0.005969 |
| 37 | rs17759661 | A | 0.017636 | 0.002461 | 0.014768 | 0.010351 | -0.0048 | 0.005703 |
| 38 | rs1887320 | A | 0.018943 | 0.002466 | 0.011519 | 0.010154 | 8.00E-04 | 0.005299 |
| 39 | rs1925148 | G | -0.01397 | 0.00248 | -0.01322 | 0.010537 | 1.00E-04 | 0.007979 |
| 40 | rs2004283 | G | -0.01367 | 0.00249 | -0.00442 | 0.011156 | 0.0038 | 0.005768 |
| 41 | rs2004776 | T | 0.019916 | 0.002882 | 0.034543 | 0.011395 | -0.0027 | 0.006769 |
| 42 | rs2017199 | A | -0.01472 | 0.002696 | 0.004334 | 0.011666 | -0.011 | 0.006178 |
| 43 | rs2052691 | A | 0.018941 | 0.002717 | -0.02453 | 0.012537 | -0.0028 | 0.00657 |
| 44 | rs2076328 | T | -0.01405 | 0.002466 | -0.00399 | 0.011429 | -0.0048 | 0.005703 |
| 45 | rs2102397 | C | -0.0178 | 0.002464 | 0.006497 | 0.011214 | 0.0026 | 0.005559 |
| 46 | rs210630 | G | -0.01553 | 0.002462 | -0.00918 | 0.010279 | -0.0068 | 0.005665 |
| 47 | rs2177843 | T | 0.023575 | 0.003496 | 0.007311 | 0.013957 | -0.0076 | 0.008303 |
| 48 | rs2249105 | G | -0.01803 | 0.002555 | 0.000646 | 0.010467 | -0.017 | 0.005767 |
| 49 | rs2274224 | C | -0.02466 | 0.002482 | 0.019105 | 0.010364 | 0.0057 | 0.005732 |
| 50 | rs2301597 | C | -0.023 | 0.002494 | -0.00103 | 0.010311 | -0.015 | 0.005792 |
| 51 | rs2303083 | A | -0.02418 | 0.003111 | -0.01388 | 0.013807 | 0.012 | 0.007718 |
| 52 | rs2306526 | T | -0.0157 | 0.002462 | -0.0135 | 0.010418 | 0.0072 | 0.005618 |
| 53 | rs2379829 | C | -0.01678 | 0.002777 | -0.0205 | 0.011531 | 0.011 | 0.007075 |
| 54 | rs2447607 | T | 0.017354 | 0.002538 | 0.011312 | 0.010436 | 0.008 | 0.006104 |
| 55 | rs2469997 | C | -0.0196 | 0.003172 | 0.003502 | 0.013987 | -0.0094 | 0.007664 |
| 56 | rs2472299 | G | -0.02167 | 0.002763 | -0.03656 | 0.010953 | -0.0048 | 0.006354 |
| 57 | rs2493296 | T | 0.024408 | 0.003582 | 0.059564 | 0.016231 | 0.0033 | 0.009944 |
| 58 | rs2627316 | G | 0.019349 | 0.002465 | 0.007339 | 0.011051 | 0.0079 | 0.005757 |
| 59 | rs2721800 | C | -0.01812 | 0.003224 | -0.00384 | 0.014497 | 0.0056 | 0.007751 |
| 60 | rs2782980 | C | 0.020191 | 0.002741 | 0.021764 | 0.01179 | 8.00E-04 | 0.007077 |
| 61 | rs28667801 | T | 0.013983 | 0.002515 | 0.00662 | 0.010732 | -0.0057 | 0.005974 |
| 62 | rs2892796 | A | -0.027 | 0.0046 | -0.01111 | 0.018003 | -0.0035 | 0.010984 |
| 63 | rs2971608 | C | 0.020858 | 0.002975 | -0.05119 | 0.012289 | -0.0047 | 0.007306 |
| 64 | rs2977334 | T | 0.016376 | 0.002508 | 0.016531 | 0.010257 | 0.018 | 0.005825 |
| 65 | rs3184504 | C | -0.02148 | 0.002461 | -0.07334 | 0.01166 | 0.0092 | 0.005593 |
| 66 | rs3211995 | A | -0.01992 | 0.003378 | 0.007416 | 0.015773 | -8.00E-04 | 0.009107 |
| 67 | rs34727427 | C | 0.01478 | 0.00264 | -0.0059 | 0.011887 | 0.0083 | 0.006333 |
| 68 | rs34896506 | C | 0.021064 | 0.003223 | 0.01556 | 0.01242 | 0.0015 | 0.00743 |
| 69 | rs35021474 | G | -0.0224 | 0.002536 | 0.013394 | 0.011498 | -0.0065 | 0.006403 |
| 70 | rs35224044 | T | 0.014976 | 0.002497 | -0.00422 | 0.01114 | -0.0077 | 0.005875 |
| 71 | rs35443 | C | -0.01831 | 0.002525 | -0.00083 | 0.010666 | -0.0059 | 0.005933 |
| 72 | rs365990 | G | -0.01665 | 0.002544 | -0.01691 | 0.011411 | 0.0036 | 0.006022 |
| 73 | rs3781885 | T | -0.01468 | 0.002654 | -0.00524 | 0.011544 | 0.015 | 0.006227 |
| 74 | rs3817581 | T | 0.018524 | 0.002463 | 0.022416 | 0.010397 | -0.0033 | 0.005662 |
| 75 | rs3826537 | G | 0.014316 | 0.002485 | -0.01445 | 0.011079 | -3.00E-04 | 0.005982 |
| 76 | rs4480845 | C | -0.01994 | 0.002578 | 0.016401 | 0.010842 | -0.0013 | 0.005713 |
| 77 | rs4639796 | A | 0.019328 | 0.003356 | -0.01412 | 0.013055 | 0.0019 | 0.0075 |
| 78 | rs4736135 | T | -0.0169 | 0.002751 | -0.00917 | 0.011809 | 0.0085 | 0.006633 |
| 79 | rs4759062 | T | -0.01527 | 0.002697 | -0.00997 | 0.011349 | 0.0036 | 0.006177 |
| 80 | rs4767332 | A | 0.015379 | 0.002499 | 0.009437 | 0.010633 | -6.00E-04 | 0.005308 |
| 81 | rs4843748 | A | -0.01549 | 0.002619 | -0.005 | 0.010832 | -0.016 | 0.006293 |
| 82 | rs488834 | T | -0.01687 | 0.002904 | -0.00695 | 0.013374 | 0.0044 | 0.007006 |
| 83 | rs4930676 | T | -0.02433 | 0.003963 | -0.02381 | 0.015099 | 0.015 | 0.009907 |
| 84 | rs4937515 | C | -0.02023 | 0.002523 | 0.00175 | 0.011296 | -4.00E-04 | 0.006379 |
| 85 | rs55925664 | A | 0.025681 | 0.00316 | 0.020079 | 0.013365 | 0.0099 | 0.007554 |
| 86 | rs56092448 | T | 0.022238 | 0.003859 | 0.020666 | 0.018356 | 0.01 | 0.009257 |
| 87 | rs6026742 | A | 0.031569 | 0.003833 | 0.05273 | 0.016076 | -0.02 | 0.008984 |
| 88 | rs604723 | C | 0.033938 | 0.00277 | 0.02883 | 0.01118 | -0.0029 | 0.006391 |
| 89 | rs62368019 | C | 0.015966 | 0.002714 | 0.006424 | 0.011634 | 0.0059 | 0.006446 |
| 90 | rs62481856 | A | 0.043879 | 0.00309 | -0.00108 | 0.012319 | -0.013 | 0.009252 |
| 91 | rs6271 | T | -0.02886 | 0.004699 | -0.05477 | 0.029424 | 0.025 | 0.015643 |
| 92 | rs6461992 | G | 0.035501 | 0.00473 | -0.00945 | 0.019709 | -0.015 | 0.011966 |
| 93 | rs6504213 | C | 0.01545 | 0.002507 | 0.039933 | 0.011078 | 0.0084 | 0.005978 |
| 94 | rs6544667 | T | -0.01393 | 0.002532 | 0.007121 | 0.010739 | 0.0024 | 0.006017 |
| 95 | rs6690557 | G | -0.01511 | 0.002728 | -0.01619 | 0.011082 | -0.0066 | 0.006501 |
| 96 | rs6733889 | C | -0.01505 | 0.002537 | 0.00069 | 0.010398 | 0.0024 | 0.006017 |
| 97 | rs6734118 | A | -0.01896 | 0.002993 | -0.01433 | 0.011673 | -0.0062 | 0.007213 |
| 98 | rs6768542 | A | -0.02054 | 0.003395 | 0.009474 | 0.014044 | -0.0094 | 0.008171 |
| 99 | rs6911827 | T | 0.014685 | 0.00247 | 0.006126 | 0.010522 | -0.0046 | 0.005829 |
| 100 | rs691830 | G | 0.016941 | 0.002463 | 0.015436 | 0.010333 | -0.001 | 0.00567 |
| 101 | rs6983129 | A | -0.0198 | 0.002479 | 0.009201 | 0.010806 | -4.00E-04 | 0.005314 |
| 102 | rs709668 | G | 0.01783 | 0.003072 | 0.031998 | 0.012417 | 0.0018 | 0.007487 |
| 103 | rs7200432 | A | -0.01907 | 0.002693 | -0.01682 | 0.011248 | -0.0046 | 0.006367 |
| 104 | rs73437338 | C | -0.03765 | 0.003308 | 0.063906 | 0.012972 | 0.016 | 0.007751 |
| 105 | rs73563812 | T | -0.02106 | 0.0029 | -0.01153 | 0.011874 | -0.0066 | 0.006637 |
| 106 | rs743395 | T | 0.014613 | 0.002585 | 0.003474 | 0.011113 | 0.0019 | 0.006499 |
| 107 | rs7439366 | C | 0.015315 | 0.002469 | 0.000427 | 0.011315 | -9.00E-04 | 0.005961 |
| 108 | rs7753358 | A | 0.014439 | 0.002503 | 0.027802 | 0.010591 | 0.0078 | 0.005551 |
| 109 | rs7765526 | G | -0.01419 | 0.002496 | 0.013543 | 0.010692 | 3.00E-04 | 0.004784 |
| 110 | rs7798991 | C | 0.0157 | 0.002799 | 0.013946 | 0.011483 | -0.0086 | 0.006562 |
| 111 | rs78473917 | C | -0.01901 | 0.003462 | -0.03471 | 0.014571 | -0.01 | 0.008153 |
| 112 | rs786910 | G | -0.01633 | 0.002499 | -0.00148 | 0.010556 | -0.012 | 0.00566 |
| 113 | rs8121509 | C | -0.01567 | 0.002471 | -0.03186 | 0.011588 | 0.0055 | 0.007123 |
| 114 | rs9294987 | C | 0.013856 | 0.002479 | -0.0146 | 0.011145 | -1.00E-04 | 0.007979 |
| 115 | rs9349379 | G | -0.01381 | 0.002505 | 0.130965 | 0.01065 | -0.0095 | 0.006599 |
| 116 | rs9385405 | C | 0.022515 | 0.002475 | 0.00177 | 0.010247 | 0.0065 | 0.005893 |
| 117 | rs9476307 | G | -0.0144 | 0.002503 | -0.00825 | 0.010659 | -0.0019 | 0.005963 |
| 118 | rs9888615 | C | 0.016954 | 0.002723 | 0.008668 | 0.011376 | -0.003 | 0.006414 |

SBP: Systolic blood pressure; MI: Myocardial infarction; AAM: Age at menarche; SNPs: Single nucleotide polymorphisms.

^a^ Effect size of each allele on genetically-determined SBP (mmHg per allele)

^b^ Standard error of the genetic association of each effect allele with genetically-determined SBP

^c^ Effect size per allele in the log-odds or the log probability of MI after adjusting for genetically predicted AAM

^d^ Standard error of the genetic association of each effect allele with genetically predicted MI after adjusting for genetically predicted AAM

^e^ Effect size per allele in AAM (in years)

^f^ Standard error of the genetic association of each effect allele with genetically predicted AAM

Table S28. Genetic estimates for the association of genetically-determined TG with genetically predicted MI after adjusting for genetically predicted AAM.

|  | SNPs | Effect allele | Z_k_^a^ | σZ_k_^b^ | Y_k_ ^c^ | σY_k_ ^d^ | X_k_^e^ | σX_k_^f^ |
| --- | --- | --- | --- | --- | --- | --- | --- | --- |
| 1 | rs10040328 | A | 0.015304 | 0.002638 | 0.00986 | 0.011728 | -0.013 | 0.006662 |
| 2 | rs1009590 | C | 0.024721 | 0.004177 | -0.00944 | 0.018658 | 0.0071 | 0.011306 |
| 3 | rs10405944 | C | -0.01303 | 0.002358 | -0.0083 | 0.011835 | -0.012 | 0.00674 |
| 4 | rs1044808 | C | -0.02819 | 0.004206 | -0.00662 | 0.024555 | -0.0059 | 0.013002 |
| 5 | rs1045241 | T | -0.02232 | 0.002625 | -0.02081 | 0.011704 | -0.0078 | 0.006638 |
| 6 | rs10772947 | G | -0.01368 | 0.002324 | 0.000284 | 0.010512 | 0.0085 | 0.005905 |
| 7 | rs10797119 | C | 0.017236 | 0.002338 | 0.022744 | 0.010392 | 0.02 | 0.0057 |
| 8 | rs10838681 | A | -0.02629 | 0.002614 | 0.019958 | 0.011544 | -0.0089 | 0.006334 |
| 9 | rs10851698 | T | 0.016157 | 0.002648 | 0.005462 | 0.011544 | 0.008 | 0.006522 |
| 10 | rs10872003 | A | -0.01308 | 0.002337 | 0.002575 | 0.010771 | -0.0039 | 0.00565 |
| 11 | rs10957299 | G | -0.01303 | 0.002342 | -0.01072 | 0.010365 | 0.0091 | 0.005694 |
| 12 | rs11030107 | G | 0.016587 | 0.00263 | 0.020543 | 0.01291 | 0.034 | 0.006644 |
| 13 | rs11045171 | G | -0.0286 | 0.002927 | -0.00077 | 0.013901 | -0.0046 | 0.008098 |
| 14 | rs11078597 | C | 0.020328 | 0.002976 | -0.00798 | 0.013707 | 0.0068 | 0.010082 |
| 15 | rs11122450 | G | -0.04754 | 0.002383 | -0.02424 | 0.01046 | -0.0082 | 0.006116 |
| 16 | rs11206374 | A | 0.025156 | 0.002781 | 0.018384 | 0.013282 | 0.0026 | 0.006992 |
| 17 | rs1128249 | T | -0.03706 | 0.002373 | -0.01074 | 0.010841 | -0.0034 | 0.005688 |
| 18 | rs11631625 | G | 0.014743 | 0.00265 | 0.014244 | 0.011717 | 0.0034 | 0.006484 |
| 19 | rs11705483 | A | 0.023944 | 0.00369 | 0.040333 | 0.017673 | 0.014 | 0.009246 |
| 20 | rs11722924 | C | 0.013532 | 0.002324 | -0.01181 | 0.010278 | 0.0027 | 0.005773 |
| 21 | rs11752394 | G | 0.017287 | 0.002735 | -0.00045 | 0.011768 | 0.011 | 0.007265 |
| 22 | rs12088739 | G | -0.02732 | 0.00404 | -0.05375 | 0.018939 | 0.001 | 0.009957 |
| 23 | rs12119979 | G | 0.020736 | 0.002328 | 0.005646 | 0.01028 | 0.0041 | 0.005675 |
| 24 | rs12281051 | C | 0.070032 | 0.010053 | 0.018099 | 0.035391 | -0.0049 | 0.022822 |
| 25 | rs12424054 | A | 0.019019 | 0.002739 | -0.00174 | 0.012248 | 6.00E-04 | 0.00683 |
| 26 | rs12446515 | T | -0.03551 | 0.002486 | -0.02643 | 0.011528 | 0.0024 | 0.006229 |
| 27 | rs12480662 | T | -0.01547 | 0.002687 | -0.01623 | 0.01284 | 0.0024 | 0.006695 |
| 28 | rs12541912 | C | -0.09644 | 0.002547 | -0.02582 | 0.011951 | -0.0038 | 0.006201 |
| 29 | rs12686780 | T | 0.016979 | 0.003061 | 0.001049 | 0.012392 | -0.009 | 0.007824 |
| 30 | rs1279840 | C | 0.028006 | 0.002684 | 0.025146 | 0.012329 | 0.0028 | 0.00702 |
| 31 | rs12868517 | G | -0.01482 | 0.002714 | 0.01268 | 0.011256 | -0.0054 | 0.006554 |
| 32 | rs13234131 | G | -0.12951 | 0.003448 | 0.010422 | 0.016854 | 0.0056 | 0.009608 |
| 33 | rs13269725 | G | 0.03532 | 0.004292 | 0.007305 | 0.017975 | -0.0069 | 0.010987 |
| 34 | rs13273454 | T | -0.05507 | 0.002327 | -0.02886 | 0.011231 | -0.0095 | 0.005944 |
| 35 | rs1340819 | C | -0.01346 | 0.002439 | -0.00054 | 0.011448 | 0.0058 | 0.006079 |
| 36 | rs1420384 | T | -0.01438 | 0.002465 | -0.00105 | 0.010796 | 0.01 | 0.00608 |
| 37 | rs142047875 | T | -0.01339 | 0.002372 | -0.02349 | 0.010403 | 0.0047 | 0.005955 |
| 38 | rs1471251 | T | 0.038485 | 0.002374 | 0.013554 | 0.011014 | -0.0013 | 0.005713 |
| 39 | rs1473886 | T | -0.01856 | 0.002323 | 0.022777 | 0.010236 | -0.0058 | 0.005596 |
| 40 | rs149142833 | T | 0.01933 | 0.003222 | 0.009455 | 0.01579 | 6.00E-04 | 0.007971 |
| 41 | rs154735 | A | 0.028025 | 0.004793 | -0.00897 | 0.019926 | -0.012 | 0.01182 |
| 42 | rs1549293 | T | -0.01572 | 0.002426 | -0.00568 | 0.011303 | -0.014 | 0.005637 |
| 43 | rs1644005 | C | -0.01437 | 0.002413 | -0.00524 | 0.011322 | 0.0086 | 0.005974 |
| 44 | rs1684608 | A | 0.01824 | 0.002945 | 0.003262 | 0.013177 | 0.002 | 0.00751 |
| 45 | rs1688043 | T | 0.028631 | 0.004665 | -0.02746 | 0.017899 | -0.018 | 0.011577 |
| 46 | rs17052058 | G | -0.03187 | 0.003006 | -0.00823 | 0.014401 | -0.027 | 0.007836 |
| 47 | rs17138358 | C | 0.018621 | 0.002374 | -0.01526 | 0.01036 | -0.012 | 0.005692 |
| 48 | rs17311740 | T | -0.02642 | 0.004631 | 0.012304 | 0.024673 | 0.0088 | 0.01218 |
| 49 | rs17326656 | T | 0.016275 | 0.002733 | 0.005844 | 0.013143 | 0.0064 | 0.006848 |
| 50 | rs174574 | C | -0.0512 | 0.002429 | 0.021068 | 0.011221 | 0.002 | 0.006841 |
| 51 | rs17496249 | G | -0.01379 | 0.002344 | -0.02543 | 0.010243 | 0.0062 | 0.005621 |
| 52 | rs17699425 | A | -0.028 | 0.004949 | 0.047418 | 0.025724 | 0.015 | 0.01223 |
| 53 | rs1790099 | T | 0.016651 | 0.002564 | 0.010012 | 0.011853 | -0.018 | 0.006524 |
| 54 | rs1800978 | G | -0.02552 | 0.003533 | -0.04313 | 0.014765 | 0.001 | 0.009957 |
| 55 | rs1853413 | G | -0.01411 | 0.002446 | -0.00038 | 0.011 | 0.012 | 0.006355 |
| 56 | rs1924485 | T | -0.0205 | 0.003431 | -0.00092 | 0.013581 | -0.011 | 0.008393 |
| 57 | rs1928496 | T | 0.01667 | 0.002647 | -0.00677 | 0.012339 | -0.0036 | 0.006505 |
| 58 | rs199607859 | T | -0.02937 | 0.002368 | -0.00462 | 0.010708 | -0.012 | 0.006254 |
| 59 | rs199795230 | T | 0.026592 | 0.003184 | -0.03396 | 0.013887 | -0.002 | 0.00751 |
| 60 | rs2068888 | A | -0.03081 | 0.002329 | -0.0433 | 0.01085 | 0.0073 | 0.005823 |
| 61 | rs2070971 | T | 0.024664 | 0.00338 | -0.00293 | 0.015149 | 0.0058 | 0.009236 |
| 62 | rs2071887 | A | 0.016832 | 0.002444 | -0.00807 | 0.010761 | 0.0089 | 0.006486 |
| 63 | rs2081687 | C | -0.02602 | 0.002453 | -0.00518 | 0.010852 | -0.0088 | 0.006113 |
| 64 | rs2114273 | C | 0.017083 | 0.002366 | -0.00021 | 0.010548 | 0.005 | 0.005941 |
| 65 | rs213479 | T | -0.01504 | 0.002336 | 0.001476 | 0.010611 | -9.00E-04 | 0.007162 |
| 66 | rs2139980 | A | -0.01488 | 0.00242 | -0.01075 | 0.010691 | 0.0031 | 0.006435 |
| 67 | rs2267373 | T | 0.022467 | 0.002352 | 0.006407 | 0.010386 | -0.011 | 0.005733 |
| 68 | rs2510344 | C | -0.01706 | 0.002323 | -0.01128 | 0.010216 | -0.0094 | 0.005647 |
| 69 | rs261342 | C | -0.04704 | 0.002812 | -0.03282 | 0.012069 | 0.0068 | 0.007276 |
| 70 | rs2652812 | T | -0.01825 | 0.002759 | -0.02542 | 0.011916 | 3.00E-04 | 0.007977 |
| 71 | rs2694913 | C | 0.014079 | 0.002425 | -0.00231 | 0.011013 | -0.0019 | 0.005725 |
| 72 | rs2699805 | A | -0.01851 | 0.002381 | -0.02707 | 0.010975 | 0.0037 | 0.006038 |
| 73 | rs2723067 | G | -0.01981 | 0.002359 | 0.009884 | 0.010994 | -0.021 | 0.005954 |
| 74 | rs2925979 | C | -0.03151 | 0.002528 | -0.01205 | 0.011783 | 0.0057 | 0.006227 |
| 75 | rs2943645 | T | 0.040295 | 0.002424 | 0.034922 | 0.010952 | 0.0047 | 0.005955 |
| 76 | rs2954017 | C | -0.08935 | 0.002321 | -0.04722 | 0.010186 | -2.00E-04 | 0.005318 |
| 77 | rs296884 | T | -0.02353 | 0.002666 | -0.01298 | 0.011369 | -0.019 | 0.006466 |
| 78 | rs2971669 | T | 0.016266 | 0.002813 | 0.008124 | 0.012636 | 0.0047 | 0.00767 |
| 79 | rs34682685 | A | 0.033067 | 0.003795 | 0.024773 | 0.020342 | 0.0068 | 0.009628 |
| 80 | rs35225200 | C | 0.031323 | 0.004333 | 0.000105 | 0.022909 | 0.023 | 0.013138 |
| 81 | rs3731696 | G | 0.020535 | 0.003546 | -0.02183 | 0.014759 | 0.039 | 0.008945 |
| 82 | rs38205 | C | -0.01486 | 0.002411 | -0.01649 | 0.010566 | 0.0092 | 0.006076 |
| 83 | rs3822072 | A | 0.016627 | 0.002331 | 0.02104 | 0.01047 | -0.0072 | 0.00587 |
| 84 | rs4410790 | C | 0.016122 | 0.00241 | 0.027445 | 0.010773 | -0.0013 | 0.006055 |
| 85 | rs45487899 | T | -0.03648 | 0.005453 | -0.06403 | 0.027764 | 0.0056 | 0.01506 |
| 86 | rs4646246 | G | 0.030376 | 0.002987 | 0.044027 | 0.013483 | -0.0037 | 0.007681 |
| 87 | rs4675812 | A | -0.01314 | 0.002355 | 0.019916 | 0.010273 | 6.00E-04 | 0.005308 |
| 88 | rs4710938 | G | -0.0144 | 0.002328 | -0.00091 | 0.01034 | 0.01 | 0.005499 |
| 89 | rs4722551 | C | -0.03714 | 0.00318 | 0.005813 | 0.015045 | -0.012 | 0.007925 |
| 90 | rs4731701 | T | -0.03368 | 0.002324 | -0.01366 | 0.010515 | -0.003 | 0.005721 |
| 91 | rs4760254 | C | -0.02841 | 0.002705 | 0.002159 | 0.013582 | -0.0031 | 0.006628 |
| 92 | rs4761234 | C | -0.01633 | 0.002328 | -0.01603 | 0.010397 | 8.00E-04 | 0.005784 |
| 93 | rs4789182 | A | 0.015239 | 0.002622 | -0.01802 | 0.01146 | -0.0039 | 0.006364 |
| 94 | rs4841580 | C | -0.02396 | 0.002347 | 0.006038 | 0.011849 | 0.0075 | 0.006248 |
| 95 | rs4843754 | G | 0.014204 | 0.002326 | 0.000927 | 0.011676 | -0.0053 | 0.006166 |
| 96 | rs4930352 | T | -0.01377 | 0.002361 | 0.001998 | 0.010561 | -0.0034 | 0.005688 |
| 97 | rs4930724 | C | -0.02658 | 0.002467 | -0.02917 | 0.011325 | 0.0013 | 0.006055 |
| 98 | rs4969179 | G | -0.01847 | 0.002374 | -0.00775 | 0.010613 | -0.0012 | 0.005944 |
| 99 | rs4976033 | G | 0.018832 | 0.002398 | 0.013976 | 0.011365 | 0.0063 | 0.007028 |
| 100 | rs5005705 | A | -0.0205 | 0.003117 | -0.02083 | 0.013931 | 0.034 | 0.007645 |
| 101 | rs5112 | G | 0.068337 | 0.002486 | 0.012207 | 0.015682 | -0.019 | 0.010142 |
| 102 | rs56030759 | C | 0.040682 | 0.00486 | -0.00464 | 0.022632 | -0.018 | 0.011888 |
| 103 | rs57232565 | T | 0.19444 | 0.005073 | 0.049875 | 0.024193 | -0.012 | 0.01182 |
| 104 | rs5755799 | G | 0.013109 | 0.002333 | 0.014633 | 0.010357 | 0.0082 | 0.005836 |
| 105 | rs57994353 | C | 0.014533 | 0.002529 | -0.03012 | 0.01236 | 0.018 | 0.006237 |
| 106 | rs58284370 | A | 0.026881 | 0.003959 | -0.02543 | 0.017354 | 0.035 | 0.013765 |
| 107 | rs58542926 | T | -0.10552 | 0.004391 | -0.04597 | 0.020412 | 0.0034 | 0.01067 |
| 108 | rs591939 | G | 0.020518 | 0.002681 | 0.036376 | 0.012141 | -0.014 | 0.006203 |
| 109 | rs593979 | C | -0.01954 | 0.002496 | -0.02336 | 0.010656 | -0.0057 | 0.005974 |
| 110 | rs6066138 | A | -0.01977 | 0.002577 | -0.00966 | 0.01199 | -0.0051 | 0.006462 |
| 111 | rs6073958 | C | 0.055687 | 0.002908 | -0.02901 | 0.012879 | 0.008 | 0.007405 |
| 112 | rs60856912 | T | 0.025468 | 0.003162 | 0.034769 | 0.013325 | -0.0036 | 0.00726 |
| 113 | rs6093446 | A | 0.014235 | 0.002566 | 0.042337 | 0.011223 | -0.0027 | 0.006546 |
| 114 | rs62112763 | G | 0.020615 | 0.002346 | 0.000347 | 0.010447 | 0.017 | 0.006014 |
| 115 | rs631106 | A | -0.08064 | 0.002427 | -0.00486 | 0.010817 | 0.0044 | 0.00623 |
| 116 | rs6486122 | T | 0.019909 | 0.002508 | 0.025128 | 0.011003 | 0.028 | 0.006282 |
| 117 | rs6492721 | C | -0.01482 | 0.002496 | -0.0112 | 0.010752 | -0.016 | 0.006143 |
| 118 | rs6506033 | T | -0.02948 | 0.004505 | -0.00948 | 0.016939 | -0.0013 | 0.011501 |
| 119 | rs6547692 | A | -0.08511 | 0.00233 | 0.000114 | 0.010438 | 0.017 | 0.005857 |
| 120 | rs6554198 | A | -0.01327 | 0.002358 | -0.01508 | 0.010255 | -0.0082 | 0.005836 |
| 121 | rs6708784 | G | -0.0131 | 0.002324 | 0.013639 | 0.010531 | 0.0082 | 0.005696 |
| 122 | rs676210 | A | -0.07658 | 0.002874 | -0.00689 | 0.011577 | -0.0043 | 0.006847 |
| 123 | rs6792725 | G | -0.01609 | 0.002592 | -0.01394 | 0.011565 | -0.0052 | 0.006448 |
| 124 | rs6882076 | C | 0.036111 | 0.002407 | 0.008908 | 0.010648 | -0.0028 | 0.005987 |
| 125 | rs6916318 | T | 0.02615 | 0.002329 | 0.006035 | 0.01019 | 0.0029 | 0.005685 |
| 126 | rs7117238 | A | -0.01894 | 0.003174 | -0.03563 | 0.01283 | -0.053 | 0.007885 |
| 127 | rs7140110 | C | 0.029279 | 0.002539 | 0.019787 | 0.013647 | -5.00E-04 | 0.006643 |
| 128 | rs71603401 | G | 0.026641 | 0.003424 | 0.031051 | 0.01475 | 0.014 | 0.00876 |
| 129 | rs7167078 | G | -0.01537 | 0.002505 | 0.019181 | 0.011257 | 0.016 | 0.006293 |
| 130 | rs7274718 | A | 0.015787 | 0.002364 | -0.01136 | 0.010382 | -0.003 | 0.005721 |
| 131 | rs729761 | G | 0.017988 | 0.002583 | 0.018271 | 0.012483 | 0.011 | 0.006468 |
| 132 | rs7298844 | G | 0.01571 | 0.002852 | -0.02912 | 0.013199 | -0.0029 | 0.007526 |
| 133 | rs73243877 | G | 0.027698 | 0.003101 | 0.019885 | 0.014781 | 0.0038 | 0.007664 |
| 134 | rs7529073 | C | -0.01299 | 0.002329 | -0.00757 | 0.010184 | -0.0086 | 0.00568 |
| 135 | rs76669111 | T | -0.02411 | 0.003198 | 0.009926 | 0.015002 | 0.0087 | 0.007887 |
| 136 | rs7735249 | G | 0.026359 | 0.003688 | 0.062762 | 0.017423 | -0.022 | 0.012855 |
| 137 | rs7826687 | G | 0.032527 | 0.00257 | 0.000613 | 0.011143 | -0.0044 | 0.006523 |
| 138 | rs7896783 | A | -0.03407 | 0.002324 | 0.010857 | 0.010164 | -0.014 | 0.005506 |
| 139 | rs7952521 | A | -0.02914 | 0.003902 | -0.01733 | 0.018497 | 0.016 | 0.011387 |
| 140 | rs8025505 | T | 0.022162 | 0.002668 | -0.01397 | 0.011814 | 0.0057 | 0.008257 |
| 141 | rs8126001 | T | -0.01806 | 0.002327 | -0.03583 | 0.012048 | 0.0055 | 0.007123 |
| 142 | rs852392 | A | 0.01607 | 0.002811 | 0.010967 | 0.013627 | -0.0072 | 0.007092 |
| 143 | rs863750 | T | 0.029472 | 0.002377 | 0.022875 | 0.01059 | -0.0035 | 0.005711 |
| 144 | rs867939 | A | -0.01551 | 0.00236 | -0.00686 | 0.010357 | -0.0094 | 0.005715 |
| 145 | rs9425589 | A | -0.01359 | 0.002341 | -0.01667 | 0.010385 | 0.0025 | 0.005683 |
| 146 | rs9692598 | G | -0.01368 | 0.002322 | 0.000666 | 0.010793 | -0.0052 | 0.005923 |
| 147 | rs9836434 | T | 0.01498 | 0.00272 | 0.007008 | 0.012694 | -0.0028 | 0.00657 |
| 148 | rs9844972 | C | 0.04177 | 0.004587 | 0.042728 | 0.023228 | 0.005 | 0.011733 |
| 149 | rs998584 | A | 0.03985 | 0.002326 | 0.047772 | 0.011036 | 0.0012 | 0.007332 |

TG: Triglycerides; MI: Myocardial infarction; AAM: Age at menarche; SNPs: Single nucleotide polymorphisms.

^a^ Effect size of each allele on genetically-determined TG (mmol/l per allele)

^b^ Standard error of the genetic association of each effect allele with genetically-determined TG

^c^ Effect size per allele in the log-odds or the log probability of MI after adjusting for genetically predicted AAM

^d^ Standard error of the genetic association of each effect allele with genetically predicted MI after adjusting for genetically predicted AAM

^e^ Effect size per allele in AAM (in years)

^f^ Standard error of the genetic association of each effect allele with genetically predicted AAM

Table S29. Genetic estimates for the association of genetically-determined alcohol intake frequency with genetically predicted MI after adjusting for genetically predicted AAM.

|  | SNPs | Effect allele | Z_k_^a^ | σZ_k_^b^ | Y_k_ ^c^ | σY_k_ ^d^ | X_k_^e^ | σX_k_^f^ |
| --- | --- | --- | --- | --- | --- | --- | --- | --- |
| 1 | rs10188314 | T | -0.01979 | 0.003036 | 0.003268 | 0.010284 | -0.0038 | 0.005907 |
| 2 | rs10792669 | G | 0.017432 | 0.003041 | 0.013323 | 0.010399 | 0.0012 | 0.005589 |
| 3 | rs11039429 | T | -0.02356 | 0.003037 | 0.000949 | 0.010414 | -0.0054 | 0.005659 |
| 4 | rs1104608 | C | 0.017421 | 0.003088 | 0.00124 | 0.011252 | 0.0075 | 0.006942 |
| 5 | rs11223617 | A | 0.025091 | 0.003754 | -0.00501 | 0.012093 | -0.01 | 0.006947 |
| 6 | rs11700855 | G | -0.0298 | 0.005233 | 0.004728 | 0.017028 | -0.002 | 0.009906 |
| 7 | rs11750777 | A | -0.02049 | 0.003726 | 0.004334 | 0.01381 | 0.0024 | 0.007856 |
| 8 | rs11787216 | T | 0.024416 | 0.003201 | -0.01385 | 0.011616 | -0.011 | 0.006114 |
| 9 | rs11940694 | G | -0.04371 | 0.003116 | 0.010302 | 0.010436 | -0.0021 | 0.005858 |
| 10 | rs12153855 | C | 0.029444 | 0.004935 | -0.00767 | 0.017252 | -0.0023 | 0.010108 |
| 11 | rs1228589 | A | 0.02107 | 0.003528 | 0.019834 | 0.012453 | 0.015 | 0.006692 |
| 12 | rs12312693 | C | -0.01768 | 0.00305 | 0.021055 | 0.010599 | 0.011 | 0.005939 |
| 13 | rs13102973 | C | -0.01941 | 0.003119 | 0.001013 | 0.01033 | -0.0042 | 0.006528 |
| 14 | rs13178443 | T | -0.01865 | 0.00339 | -0.0066 | 0.01199 | 0.0033 | 0.00647 |
| 15 | rs13390019 | C | 0.029612 | 0.004492 | 0.009628 | 0.018067 | -3.00E-04 | 0.011967 |
| 16 | rs1421085 | C | 0.019939 | 0.003085 | 0.026556 | 0.010737 | 0.041 | 0.006057 |
| 17 | rs1666658 | C | 0.017967 | 0.003099 | 0.024276 | 0.011623 | 0.0068 | 0.006037 |
| 18 | rs17690703 | T | 0.025034 | 0.00343 | 0.041524 | 0.015104 | -0.0048 | 0.006796 |
| 19 | rs186347 | T | 0.017949 | 0.003051 | -0.00343 | 0.010934 | -0.0022 | 0.005916 |
| 20 | rs1893659 | A | -0.02933 | 0.003053 | -0.01191 | 0.010389 | -0.0093 | 0.005637 |
| 21 | rs1937522 | G | 0.016898 | 0.003032 | 0.011899 | 0.010349 | 5.00E-04 | 0.006643 |
| 22 | rs1991083 | T | -0.02239 | 0.003258 | -0.03144 | 0.011705 | 0.022 | 0.006527 |
| 23 | rs2043677 | T | 0.026113 | 0.004327 | 0.006193 | 0.014304 | 0.0024 | 0.007856 |
| 24 | rs2159935 | A | -0.01857 | 0.003026 | -0.02236 | 0.010156 | -0.0061 | 0.005646 |
| 25 | rs2160935 | T | -0.01872 | 0.003091 | -0.0148 | 0.010584 | -0.0087 | 0.005895 |
| 26 | rs2411453 | G | -0.03508 | 0.00309 | -0.01763 | 0.012324 | -0.0029 | 0.005849 |
| 27 | rs2535911 | T | -0.01885 | 0.003168 | -0.00348 | 0.011333 | -0.013 | 0.00584 |
| 28 | rs2622167 | A | -0.01912 | 0.003067 | 0.017052 | 0.01334 | 0.0095 | 0.006923 |
| 29 | rs262240 | T | -0.01721 | 0.003035 | 0.004332 | 0.010455 | 0.01 | 0.005693 |
| 30 | rs2717063 | A | -0.02037 | 0.003085 | 0.005304 | 0.010379 | -0.014 | 0.005918 |
| 31 | rs28622224 | T | -0.01862 | 0.003368 | -0.0159 | 0.011567 | -0.0012 | 0.007949 |
| 32 | rs28787109 | A | 0.017811 | 0.003085 | -0.00787 | 0.010546 | 0.0011 | 0.005816 |
| 33 | rs2924321 | A | -0.01951 | 0.00305 | -0.00343 | 0.010505 | -0.014 | 0.005918 |
| 34 | rs34440851 | T | -0.02268 | 0.004151 | 0.024004 | 0.013686 | -5.00E-04 | 0.007974 |
| 35 | rs34631026 | T | -0.01691 | 0.003048 | -0.00286 | 0.010726 | -0.016 | 0.00589 |
| 36 | rs35105141 | T | 0.026345 | 0.003088 | -0.0095 | 0.010609 | 0.019 | 0.00569 |
| 37 | rs4241258 | T | 0.025064 | 0.004403 | 0.014614 | 0.015727 | -0.014 | 0.00876 |
| 38 | rs4242715 | A | -0.01865 | 0.003248 | 0.002657 | 0.011466 | 2.00E-04 | 0.007978 |
| 39 | rs4417025 | A | -0.01884 | 0.003165 | -0.01488 | 0.012436 | 0.0045 | 0.006995 |
| 40 | rs461599 | C | -0.01919 | 0.00304 | -0.01571 | 0.010341 | -0.0051 | 0.005689 |
| 41 | rs4726481 | T | 0.021761 | 0.003102 | 0.014595 | 0.011769 | -0.0037 | 0.006038 |
| 42 | rs473098 | T | -0.02174 | 0.003043 | -0.00088 | 0.011353 | -0.0045 | 0.005702 |
| 43 | rs489062 | A | 0.01665 | 0.003053 | 0.025068 | 0.010519 | 0.0076 | 0.005799 |
| 44 | rs4916723 | C | 0.023948 | 0.0031 | -0.00105 | 0.010684 | 0.0099 | 0.005803 |
| 45 | rs4940926 | C | -0.0191 | 0.003441 | -0.04481 | 0.011597 | -0.0084 | 0.006701 |
| 46 | rs5022348 | T | 0.020264 | 0.00357 | 0.009518 | 0.010396 | 0.0045 | 0.005702 |
| 47 | rs550942 | T | 0.022401 | 0.003989 | 0.01433 | 0.015068 | 0.019 | 0.008101 |
| 48 | rs58905411 | A | -0.02663 | 0.003078 | -0.01717 | 0.010709 | -0.007 | 0.005832 |
| 49 | rs6030200 | A | -0.01953 | 0.003271 | -0.01285 | 0.011116 | -0.0073 | 0.006213 |
| 50 | rs62097995 | A | 0.020002 | 0.003067 | 0.00775 | 0.01098 | -0.0046 | 0.005704 |
| 51 | rs650558 | T | 0.020736 | 0.003508 | 0.036907 | 0.012125 | -0.017 | 0.007585 |
| 52 | rs6727281 | T | -0.02432 | 0.00392 | -0.00971 | 0.014591 | -0.016 | 0.007328 |
| 53 | rs71651683 | T | -0.07046 | 0.012791 | 0.040066 | 0.046175 | 0.0015 | 0.023921 |
| 54 | rs72769229 | T | -0.02314 | 0.004192 | -0.01338 | 0.015303 | -0.001 | 0.007958 |
| 55 | rs728538 | G | 0.022875 | 0.004063 | 0.036543 | 0.014908 | 0.0056 | 0.007413 |
| 56 | rs7302200 | A | -0.01842 | 0.003198 | -0.00659 | 0.012174 | 0.017 | 0.006042 |
| 57 | rs73050128 | A | -0.026 | 0.004091 | -0.02821 | 0.012936 | -0.007 | 0.007336 |
| 58 | rs7330939 | T | -0.02133 | 0.003405 | -0.01382 | 0.011489 | -0.014 | 0.006604 |
| 59 | rs7610856 | A | -0.02386 | 0.00307 | -0.01066 | 0.011061 | -0.0055 | 0.005764 |
| 60 | rs780094 | C | -0.05099 | 0.003105 | 0.003123 | 0.010684 | 0.017 | 0.005857 |
| 61 | rs780569 | A | 0.019803 | 0.003365 | 0.014066 | 0.011415 | 0.0063 | 0.006335 |
| 62 | rs838145 | A | 0.021955 | 0.003055 | 0.000915 | 0.011417 | 0.013 | 0.006027 |
| 63 | rs8614 | A | 0.024781 | 0.003925 | 0.018184 | 0.012998 | -0.015 | 0.00762 |
| 64 | rs9349379 | G | -0.01935 | 0.003082 | 0.130965 | 0.01065 | -0.0095 | 0.006599 |
| 65 | rs9372625 | A | -0.02556 | 0.003125 | -0.00808 | 0.010989 | 0.0043 | 0.005952 |
| 66 | rs9403297 | A | 0.018823 | 0.00313 | 0.007726 | 0.010939 | 0.023 | 0.006141 |
| 67 | rs9648478 | A | 0.01686 | 0.003029 | 0.001866 | 0.010147 | 0.0041 | 0.005549 |
| 68 | rs9814516 | T | -0.02511 | 0.003556 | -0.00168 | 0.011646 | -0.017 | 0.006686 |
| 69 | rs9829192 | T | 0.016932 | 0.00305 | 0.003832 | 0.010321 | -0.0027 | 0.00595 |
| 70 | rs9958320 | C | 0.024855 | 0.004271 | -0.01883 | 0.014217 | -0.012 | 0.009997 |

MI: Myocardial infarction; AAM: Age at menarche; SNPs: Single nucleotide polymorphisms.

^a^ Effect size of each allele on genetically-determined alcohol intake frequency (times per week/ allele)

^b^ Standard error of the genetic association of each effect allele with genetically-determined alcohol intake frequency

^c^ Effect size per allele in the log-odds or the log probability of MI after adjusting for genetically predicted AAM

^d^ Standard error of the genetic association of each effect allele with genetically predicted MI after adjusting for genetically predicted AAM

^e^ Effect size per allele in AAM (in years)

^f^ Standard error of the genetic association of each effect allele with genetically predicted AAM

Table S30. Genetic estimates for the association of genetically-determined sleep duration with genetically predicted MI after adjusting for genetically predicted AAM.

|  | SNPs | Effect allele | Z_k_^a^ | σZ_k_^b^ | Y_k_ ^c^ | σY_k_ ^d^ | X_k_^e^ | σX_k_^f^ |
| --- | --- | --- | --- | --- | --- | --- | --- | --- |
| 1 | rs10510128 | A | 0.011403 | 0.001974 | -0.00524 | 0.013645 | 0.0046 | 0.00715 |
| 2 | rs113021516 | C | 0.011481 | 0.001697 | 0.033967 | 0.011038 | -0.01 | 0.005825 |
| 3 | rs113113059 | C | -0.01113 | 0.001933 | 0.017486 | 0.012096 | 0.017 | 0.007057 |
| 4 | rs11621908 | T | -0.01999 | 0.002943 | -0.02854 | 0.019506 | -0.018 | 0.01065 |
| 5 | rs11650677 | A | 0.01117 | 0.00169 | -0.01451 | 0.010945 | -0.0095 | 0.005944 |
| 6 | rs11982852 | T | -0.01173 | 0.001862 | 0.011026 | 0.012267 | 0.02 | 0.006739 |
| 7 | rs12518468 | C | -0.01064 | 0.001703 | 0.005875 | 0.011222 | 0.014 | 0.006203 |
| 8 | rs12567114 | A | 0.012338 | 0.001794 | 0.014131 | 0.011675 | -0.0081 | 0.00632 |
| 9 | rs13107325 | T | -0.02427 | 0.003039 | -0.00152 | 0.024398 | 0.023 | 0.013138 |
| 10 | rs1348047 | T | -0.01264 | 0.00182 | 0.014916 | 0.011858 | 0.011 | 0.006883 |
| 11 | rs1463053 | A | 0.00927 | 0.001661 | 0.011519 | 0.010594 | -0.019 | 0.005908 |
| 12 | rs1517572 | C | 0.011659 | 0.001622 | 0.010476 | 0.010609 | -0.0092 | 0.005917 |
| 13 | rs1553132 | G | 0.010526 | 0.001825 | 0.025485 | 0.012102 | 2.00E-04 | 0.005318 |
| 14 | rs1611719 | A | -0.01321 | 0.002027 | -0.00998 | 0.01706 | 0.02 | 0.019297 |
| 15 | rs17391944 | G | 0.021852 | 0.003724 | 0.049681 | 0.025341 | 0.014 | 0.013508 |
| 16 | rs174564 | G | 0.009745 | 0.001678 | -0.02368 | 0.011343 | 0.0021 | 0.006591 |
| 17 | rs17732997 | G | -0.00884 | 0.001618 | 0.013097 | 0.010382 | -0.0073 | 0.005696 |
| 18 | rs1972712 | C | 0.011796 | 0.001848 | -0.00262 | 0.011972 | -0.0013 | 0.006874 |
| 19 | rs2079070 | G | -0.01344 | 0.001811 | 0.01181 | 0.012717 | 0.011 | 0.006668 |
| 20 | rs2186122 | T | -0.01083 | 0.001621 | 0.015033 | 0.010285 | 0.01 | 0.005655 |
| 21 | rs2192528 | G | -0.00981 | 0.001601 | -0.00237 | 0.010383 | -0.004 | 0.005663 |
| 22 | rs2279681 | G | 0.009299 | 0.001685 | -0.01558 | 0.010865 | -0.0058 | 0.007192 |
| 23 | rs2683630 | G | 0.014951 | 0.001656 | 0.002142 | 0.010428 | -0.014 | 0.005969 |
| 24 | rs2734831 | G | -0.0098 | 0.001639 | -0.01839 | 0.010569 | -0.0071 | 0.005789 |
| 25 | rs2748809 | C | -0.00925 | 0.001645 | -0.02721 | 0.011564 | -0.0096 | 0.006669 |
| 26 | rs2839753 | C | -0.01064 | 0.001812 | 0.011885 | 0.011672 | -0.011 | 0.007075 |
| 27 | rs2863957 | A | 0.028904 | 0.001929 | 0.004794 | 0.012032 | -0.0029 | 0.007031 |
| 28 | rs34354917 | A | -0.01002 | 0.001768 | 0.031316 | 0.013561 | -0.016 | 0.007138 |
| 29 | rs34786000 | T | 0.010958 | 0.001628 | -0.00691 | 0.011109 | -0.038 | 0.005737 |
| 30 | rs35662245 | A | 0.010157 | 0.001691 | -0.00372 | 0.01076 | -0.0058 | 0.005954 |
| 31 | rs365663 | G | -0.00928 | 0.00161 | 0.002434 | 0.010422 | -0.0035 | 0.006005 |
| 32 | rs374153 | T | -0.0131 | 0.002197 | 0.008428 | 0.01526 | 0.0048 | 0.00803 |
| 33 | rs4767550 | G | 0.010873 | 0.001633 | -0.0215 | 0.010692 | -0.0089 | 0.005878 |
| 34 | rs55658675 | T | -0.00969 | 0.001675 | 0.009931 | 0.011278 | -0.0049 | 0.006076 |
| 35 | rs56367859 | G | 0.011622 | 0.001636 | -0.00886 | 0.011296 | -0.0072 | 0.005744 |
| 36 | rs62444917 | C | 0.012963 | 0.001926 | -0.02676 | 0.011822 | 0.011 | 0.008016 |
| 37 | rs6783516 | T | -0.00984 | 0.001631 | 0.002242 | 0.01034 | -0.0068 | 0.005665 |
| 38 | rs6889592 | A | 0.011766 | 0.001697 | -0.01283 | 0.01136 | -0.011 | 0.006049 |
| 39 | rs7016314 | C | 0.010001 | 0.001688 | -0.00521 | 0.011777 | 7.00E-04 | 0.006193 |
| 40 | rs7115856 | C | 0.010819 | 0.001603 | -0.01153 | 0.010444 | -0.012 | 0.006014 |
| 41 | rs72771082 | G | 0.010974 | 0.001936 | -0.00135 | 0.012236 | 0.027 | 0.006687 |
| 42 | rs7517981 | C | -0.00997 | 0.001634 | -0.00121 | 0.010948 | 0.017 | 0.005923 |
| 43 | rs7644809 | C | -0.01015 | 0.001625 | 0.003904 | 0.010729 | 0.0022 | 0.005916 |
| 44 | rs7711696 | T | -0.00987 | 0.001735 | 0.017925 | 0.011299 | -0.0075 | 0.006383 |
| 45 | rs7740402 | G | -0.00951 | 0.001735 | -0.00579 | 0.010715 | -3.00E-04 | 0.005982 |
| 46 | rs7831557 | A | -0.01057 | 0.001601 | 0.004658 | 0.011079 | -0.0098 | 0.006132 |
| 47 | rs8038326 | G | -0.01338 | 0.001793 | 0.009168 | 0.01203 | 0.007 | 0.006346 |
| 48 | rs8047587 | T | -0.01102 | 0.001613 | 0.017929 | 0.0105 | 0.042 | 0.005799 |
| 49 | rs8074498 | A | -0.00933 | 0.001634 | -0.01236 | 0.012737 | 0.0059 | 0.006581 |
| 50 | rs915416 | G | -0.01273 | 0.00176 | -0.00862 | 0.010967 | 0.0076 | 0.006196 |
| 51 | rs9302680 | A | 0.012044 | 0.001611 | -0.00232 | 0.010218 | -0.011 | 0.005588 |
| 52 | rs9345234 | C | 0.009192 | 0.001624 | 0.005039 | 0.010214 | -0.0064 | 0.005802 |
| 53 | rs9382445 | C | -0.00948 | 0.001649 | 0.010108 | 0.010622 | 0.029 | 0.005914 |
| 54 | rs9611007 | T | -0.01359 | 0.002297 | -0.00804 | 0.015008 | 0.026 | 0.010793 |
| 55 | rs9810474 | T | -0.01115 | 0.001894 | 0.020453 | 0.011846 | 0.028 | 0.006797 |
| 56 | rs9903898 | T | -0.00945 | 0.001601 | 0.004277 | 0.010835 | -0.012 | 0.005872 |

MI: Myocardial infarction; AAM: Age at menarche; SNPs: Single nucleotide polymorphisms.

^a^ Effect size of each allele on genetically-determined sleep duration (hours per allele)

^b^ Standard error of the genetic association of each effect allele with genetically-determined sleep duration

^c^ Effect size per allele in the log-odds or the log probability of MI after adjusting for genetically predicted AAM

^d^ Standard error of the genetic association of each effect allele with genetically predicted MI after adjusting for genetically predicted AAM

^e^ Effect size per allele in AAM (in years)

^f^ Standard error of the genetic association of each effect allele with genetically predicted AAM

Table S31. Genetic estimates for the association of genetically-determined WHR with genetically predicted MI after adjusting for genetically predicted AAM.

|  | SNPs | Effect allele | Z_k_^a^ | σZ_k_^b^ | Y_k_ ^c^ | σY_k_ ^d^ | X_k_^e^ | σX_k_^f^ |
| --- | --- | --- | --- | --- | --- | --- | --- | --- |
| 1 | rs10195252 | C | -0.044 | 0.0044 | -0.01132 | 0.010766 | -0.0035 | 0.005855 |
| 2 | rs1045241 | T | -0.027 | 0.0049 | -0.02081 | 0.011704 | -0.0078 | 0.006638 |
| 3 | rs10804591 | A | 0.035 | 0.0054 | 0.016073 | 0.011969 | 0.013 | 0.007019 |
| 4 | rs11048470 | T | 0.033 | 0.0049 | -0.00787 | 0.01175 | -0.0071 | 0.006437 |
| 5 | rs11075985 | A | 0.039 | 0.0045 | 0.021167 | 0.01041 | 0.041 | 0.005927 |
| 6 | rs11989744 | T | -0.035 | 0.0061 | 0.023125 | 0.011953 | -0.0058 | 0.006891 |
| 7 | rs12679556 | G | 0.03 | 0.0052 | 0.015351 | 0.011247 | -0.0063 | 0.006603 |
| 8 | rs1294421 | G | 0.033 | 0.0046 | -0.0218 | 0.010584 | -2.00E-04 | 0.005318 |
| 9 | rs1358980 | T | 0.049 | 0.0048 | 0.039235 | 0.010749 | 0.0028 | 0.00657 |
| 10 | rs1394461 | C | 0.035 | 0.0063 | -0.01281 | 0.012121 | -0.0071 | 0.006994 |
| 11 | rs1443512 | C | -0.037 | 0.0051 | -0.00387 | 0.011656 | -9.00E-04 | 0.007162 |
| 12 | rs17819328 | G | 0.032 | 0.0046 | 0.042399 | 0.010594 | 0.0014 | 0.006153 |
| 13 | rs1936805 | T | 0.044 | 0.0044 | 0.004434 | 0.010234 | 0.002 | 0.005795 |
| 14 | rs2179129 | G | -0.026 | 0.0045 | -0.014 | 0.010352 | -0.0091 | 0.005853 |
| 15 | rs2765539 | T | 0.031 | 0.005 | -0.00924 | 0.011289 | 0.005 | 0.006475 |
| 16 | rs3902751 | A | 0.032 | 0.0051 | 0.002669 | 0.011379 | 0.0088 | 0.006715 |
| 17 | rs4471313 | T | 0.03 | 0.0052 | 0.005467 | 0.011357 | 0.0063 | 0.006335 |
| 18 | rs4646404 | A | -0.03 | 0.0051 | -0.0036 | 0.012258 | 0.0091 | 0.006787 |
| 19 | rs4846565 | A | -0.048 | 0.0046 | -0.00013 | 0.011062 | -0.0077 | 0.006008 |
| 20 | rs489693 | A | 0.029 | 0.0047 | 0.040496 | 0.01092 | -0.0065 | 0.006143 |
| 21 | rs863750 | T | 0.027 | 0.0045 | 0.022875 | 0.01059 | -0.0035 | 0.005711 |
| 22 | rs9687846 | A | 0.034 | 0.0057 | 0.028097 | 0.013418 | 0.0082 | 0.00759 |
| 23 | rs9860730 | G | -0.042 | 0.0048 | 0.000509 | 0.011159 | -0.0073 | 0.006346 |

WHR: Waist-hip-ratio; MI: Myocardial infarction; AAM: Age at menarche; SNPs: Single nucleotide polymorphisms.

^a^ Effect size of each allele on genetically-determined WHR (percentage point/ allele)

^b^ Standard error of the genetic association of each effect allele with genetically-determined WHR

^c^ Effect size per allele in the log-odds or the log probability of MI after adjusting for genetically predicted AAM

^d^ Standard error of the genetic association of each effect allele with genetically predicted MI after adjusting for genetically predicted AAM

^e^ Effect size per allele in AAM (in years)

^f^ Standard error of the genetic association of each effect allele with genetically predicted AAM

Table S32. Genetic estimates for the association of genetically-determined FBG with genetically predicted MI after adjusting for genetically predicted AAM.

|  | SNPs | Effect allele | Z_k_^a^ | σZ_k_^b^ | Y_k_ ^c^ | σY_k_ ^d^ | X_k_^e^ | σX_k_^f^ |
| --- | --- | --- | --- | --- | --- | --- | --- | --- |
| 1 | rs10276674 | C | 0.036 | 0.0051 | 0.007199 | 0.012756 | -0.013 | 0.007903 |
| 2 | rs10787312 | A | 0.042 | 0.0062 | 0.010518 | 0.015461 | 0.0061 | 0.009954 |
| 3 | rs10830963 | G | 0.079 | 0.0045 | 0.022073 | 0.011736 | 0.012 | 0.007252 |
| 4 | rs11558471 | G | -0.027 | 0.004 | -0.00417 | 0.011393 | -0.0068 | 0.006294 |
| 5 | rs11717195 | C | -0.029 | 0.0047 | -0.01019 | 0.012765 | -0.006 | 0.006835 |
| 6 | rs12805422 | A | -0.023 | 0.0037 | 0.00419 | 0.010498 | 9.00E-04 | 0.005499 |
| 7 | rs17390909 | G | -0.037 | 0.0067 | -0.01799 | 0.01733 | -0.016 | 0.010291 |
| 8 | rs2191349 | T | 0.03 | 0.0036 | 0.007486 | 0.0103 | -0.0029 | 0.005685 |
| 9 | rs2524299 | T | -0.03 | 0.0052 | -0.02819 | 0.01585 | -0.005 | 0.008579 |
| 10 | rs4506565 | T | 0.023 | 0.004 | 0.02087 | 0.011236 | -0.01 | 0.006432 |
| 11 | rs560887 | C | 0.075 | 0.0041 | -0.0051 | 0.012225 | -0.014 | 0.006246 |
| 12 | rs6975024 | C | 0.062 | 0.0049 | 0.00916 | 0.013856 | 0.0028 | 0.007811 |
| 13 | rs780093 | C | 0.027 | 0.0037 | 0.002801 | 0.010608 | 0.017 | 0.005938 |
| 14 | rs7944584 | T | -0.025 | 0.0041 | 0.015569 | 0.012314 | -0.0074 | 0.00657 |

FBG: Fasting blood glucose; MI: Myocardial infarction; AAM: Age at menarche; SNPs: Single nucleotide polymorphisms.

^a^ Effect size of each allele on genetically-determined FBG (mmol/l per allele)

^b^ Standard error of the genetic association of each effect allele with genetically-determined FBG

^c^ Effect size per allele in the log-odds or the log probability of MI after adjusting for genetically predicted AAM

^d^ Standard error of the genetic association of each effect allele with genetically predicted MI after adjusting for genetically predicted AAM

^e^ Effect size per allele in AAM (in years)

^f^ Standard error of the genetic association of each effect allele with genetically predicted AAM

Table S33. Genetic estimates for the association of genetically-determined HDL with genetically predicted MI after adjusting for genetically predicted AAM.

|  | SNPs | Effect allele | Z_k_^a^ | σZ_k_^b^ | Y_k_ ^c^ | σY_k_ ^d^ | X_k_^e^ | σX_k_^f^ |
| --- | --- | --- | --- | --- | --- | --- | --- | --- |
| 1 | rs11632618 | A | 0.215139 | 0.023086 | 0.038836 | 0.02434 | -0.0014 | 0.01394 |
| 2 | rs174583 | T | -0.07783 | 0.010132 | -0.02396 | 0.01139 | 0.0019 | 0.00622 |
| 3 | rs1883025 | T | -0.07216 | 0.012455 | -0.02581 | 0.011584 | -0.012 | 0.0069 |
| 4 | rs247617 | A | 0.209633 | 0.01079 | -0.02614 | 0.011415 | 0.0024 | 0.006229 |
| 5 | rs261291 | C | 0.179384 | 0.010165 | 0.005162 | 0.010716 | 0.0039 | 0.00592 |
| 6 | rs291 | C | 0.10065 | 0.011676 | -0.03487 | 0.012907 | 0.0012 | 0.006804 |
| 7 | rs60439253 | T | 0.235306 | 0.028232 | 0.017901 | 0.030954 | -0.016 | 0.017848 |
| 8 | rs6065904 | A | -0.13534 | 0.011671 | -0.02312 | 0.01205 | 0.011 | 0.007075 |
| 9 | rs6507939 | C | 0.094757 | 0.013468 | -0.01747 | 0.015114 | 0.0049 | 0.008197 |
| 10 | rs6544366 | T | 0.064093 | 0.011001 | -0.01162 | 0.01142 | -4.00E-04 | 0.007976 |
| 11 | rs67053123 | A | 0.092051 | 0.014556 | 0.02784 | 0.015985 | 0.012 | 0.009573 |
| 12 | rs964184 | C | 0.081863 | 0.014048 | -0.04877 | 0.013873 | 0.0095 | 0.008794 |

HDL: High-density lipoprotein; MI: Myocardial infarction; AAM: Age at menarche; SNPs: Single nucleotide polymorphisms.

^a^ Effect size of each allele on genetically-determined HDL (mmol/l per allele)

^b^ Standard error of the genetic association of each effect allele with genetically-determined HDL

^c^ Effect size per allele in the log-odds or the log probability of MI after adjusting for genetically predicted AAM

^d^ Standard error of the genetic association of each effect allele with genetically predicted MI after adjusting for genetically predicted AAM

^e^ Effect size per allele in AAM (in years)

^f^ Standard error of the genetic association of each effect allele with genetically predicted AAM

Table S34. Genetic estimates for the association of genetically-determined age that started HRT with genetically predicted MI after adjusting for genetically predicted AAM.

|  | SNPs | Effect allele | | Z_k_^a^ | σZ_k_^b^ | Y_k_ ^c^ | σY_k_ ^d^ | X_k_^e^ | σX_k_^f^ |
| --- | --- | --- | --- | --- | --- | --- | --- | --- | --- |
| 1 | rs11668344 | G | -0.05402 | | 0.004908 | 0.017417 | 0.010759 | 0.0071 | 0.005915 |
| 2 | rs12503643 | T | 0.028498 | | 0.004856 | 0.010543 | 0.010424 | 0.004 | 0.00593 |
| 3 | rs16991615 | A | 0.105051 | | 0.009857 | -0.02119 | 0.023848 | 0.0053 | 0.013288 |
| 4 | rs177404 | C | -0.02959 | | 0.005308 | 0.00685 | 0.012926 | 0.018 | 0.007609 |
| 5 | rs2013097 | C | -0.02684 | | 0.004801 | -0.00082 | 0.010506 | -0.0036 | 0.006865 |
| 6 | rs251848 | A | 0.031416 | | 0.004847 | 0.012346 | 0.010705 | 0.0075 | 0.006383 |
| 7 | rs274721 | T | -0.02823 | | 0.004877 | 0.019009 | 0.010359 | 0.011 | 0.005983 |
| 8 | rs3743590 | A | -0.03746 | | 0.004893 | -0.01015 | 0.01062 | 3.00E-04 | 0.005982 |
| 9 | rs3765637 | T | -0.03465 | | 0.005628 | 0.009092 | 0.011791 | -0.0078 | 0.006781 |
| 10 | rs4235062 | A | 0.033026 | | 0.004755 | 0.014673 | 0.010449 | -0.017 | 0.005688 |
| 11 | rs4532801 | G | -0.03639 | | 0.005081 | 0.000455 | 0.010576 | 0.0071 | 0.006172 |
| 12 | rs6496571 | C | -0.02835 | | 0.004878 | 0.000483 | 0.010578 | 0.0028 | 0.005812 |
| 13 | rs6760293 | T | 0.028591 | | 0.004889 | 0.007413 | 0.010951 | 0.0082 | 0.005976 |
| 14 | rs6760857 | A | -0.04644 | | 0.007732 | 0.014559 | 0.018579 | -0.022 | 0.009218 |
| 15 | rs6980805 | C | 0.028853 | | 0.004865 | -0.0081 | 0.010862 | -0.0018 | 0.005892 |
| 16 | rs732084 | C | 0.030272 | | 0.005055 | -0.01931 | 0.010905 | -3.00E-04 | 0.005982 |
| 17 | rs75779608 | T | -0.03283 | | 0.005893 | -0.01191 | 0.013632 | 0.018 | 0.006871 |
| 18 | rs7994166 | T | 0.028464 | | 0.004768 | -0.00872 | 0.01046 | -0.0036 | 0.006505 |
| 19 | rs9348724 | G | -0.03823 | | 0.00636 | 0.026852 | 0.0132 | -0.015 | 0.007881 |

HRT: hormone-replacement therapy; MI: Myocardial infarction; AAM: Age at menarche; SNPs: Single nucleotide polymorphisms.

^a^ Effect size of each allele on genetically-determined age that started HRT (years/allele)

^b^ Standard error of the genetic association of each effect allele with genetically-determined age that started HRT

^c^ Effect size per allele in the log-odds or the log probability of MI after adjusting for genetically predicted AAM

^d^ Standard error of the genetic association of each effect allele with genetically predicted MI after adjusting for genetically predicted AAM

^e^ Effect size per allele in AAM (in years)

^f^ Standard error of the genetic association of each effect allele with genetically predicted AAM

Table S35. Single SNP analysis for estimate of the association of genetically predicted AAM with genetically predicted MI.

| SNPs | Beta | SE | *p*-value |
| --- | --- | --- | --- |
| rs10144321 | -0.206 | 0.288 | 0.473 |
| rs10483727 | -0.563 | 0.286 | 0.050 |
| rs1079866 | 0.140 | 0.204 | 0.492 |
| rs10840031 | -0.219 | 0.290 | 0.452 |
| rs10938397 | -0.591 | 0.272 | 0.030 |
| rs11022756 | -0.711 | 0.236 | 0.003 |
| rs11715566 | -0.217 | 0.196 | 0.270 |
| rs11756454 | -0.333 | 0.299 | 0.267 |
| rs11767400 | -0.218 | 0.353 | 0.536 |
| rs12003641 | -0.199 | 0.209 | 0.339 |
| rs12148769 | 0.234 | 0.302 | 0.439 |
| rs12291726 | -0.628 | 0.227 | 0.006 |
| rs12598642 | -0.499 | 0.242 | 0.040 |
| rs12915845 | -0.105 | 0.303 | 0.730 |
| rs13179411 | 0.122 | 0.256 | 0.635 |
| rs13215865 | 0.140 | 0.343 | 0.683 |
| rs1398217 | -0.059 | 0.224 | 0.792 |
| rs1482853 | -0.411 | 0.281 | 0.144 |
| rs1516883 | -0.037 | 0.123 | 0.760 |
| rs1518080 | -0.210 | 0.208 | 0.311 |
| rs1659127 | -0.097 | 0.258 | 0.706 |
| rs16938437 | -0.348 | 0.268 | 0.194 |
| rs17351680 | -0.737 | 0.325 | 0.023 |
| rs1874984 | 0.170 | 0.287 | 0.554 |
| rs2153127 | -0.209 | 0.136 | 0.126 |
| rs2179786 | -0.199 | 0.265 | 0.452 |
| rs2184968 | 0.942 | 0.296 | 0.001 |
| rs2303100 | -0.126 | 0.275 | 0.649 |
| rs2344508 | 0.123 | 0.305 | 0.687 |
| rs2617056 | -0.139 | 0.317 | 0.662 |
| rs2687729 | 0.354 | 0.260 | 0.173 |
| rs2836950 | 0.297 | 0.306 | 0.331 |
| rs2947411 | -0.160 | 0.256 | 0.531 |
| rs3733632 | -0.194 | 0.258 | 0.451 |
| rs3743266 | -0.319 | 0.256 | 0.213 |
| rs3870341 | 0.299 | 0.296 | 0.311 |
| rs3914188 | -0.017 | 0.266 | 0.950 |
| rs4242496 | -0.498 | 0.339 | 0.142 |
| rs4369815 | -0.418 | 0.286 | 0.144 |
| rs466639 | 0.023 | 0.195 | 0.904 |
| rs4801589 | 0.152 | 0.321 | 0.635 |
| rs4840086 | -0.369 | 0.287 | 0.200 |
| rs618678 | 0.167 | 0.333 | 0.616 |
| rs633715 | -0.564 | 0.254 | 0.026 |
| rs6694738 | 0.155 | 0.316 | 0.624 |
| rs6747380 | 0.448 | 0.204 | 0.028 |
| rs6758290 | -0.029 | 0.263 | 0.912 |
| rs6770162 | 0.110 | 0.297 | 0.711 |
| rs6933660 | 0.131 | 0.301 | 0.664 |
| rs7103411 | -0.632 | 0.281 | 0.025 |
| rs7119712 | -0.310 | 0.308 | 0.315 |
| rs740077 | 0.092 | 0.258 | 0.721 |
| rs7642134 | 0.227 | 0.275 | 0.409 |
| rs7821178 | 0.256 | 0.235 | 0.277 |
| rs7853970 | -0.550 | 0.288 | 0.056 |
| rs7944630 | 0.250 | 0.223 | 0.262 |
| rs852069 | -0.171 | 0.295 | 0.562 |
| rs888345 | 0.263 | 0.291 | 0.367 |
| rs895526 | -0.202 | 0.306 | 0.509 |
| rs913588 | -0.408 | 0.305 | 0.182 |
| rs9373571 | 0.244 | 0.313 | 0.436 |
| rs9555810 | -0.039 | 0.245 | 0.873 |
| rs9565073 | 0.262 | 0.312 | 0.402 |
| rs9635759 | -0.238 | 0.210 | 0.258 |
| rs9647570 | 0.376 | 0.301 | 0.211 |
| rs9939609 | -0.572 | 0.255 | 0.025 |
| rs9997604 | 0.589 | 0.303 | 0.052 |

AAM: Age at menarche; MI: Myocardial infarction; SNPs: Single nucleotide polymorphisms.SE: Standard deviation.

Table S36. Leave-one-out analysis for estimate of the association of genetically predicted AAM with genetically predicted MI.

| SNPs | Beta | SE | *p*-value |
| --- | --- | --- | --- |
| rs10144321 | -0.098 | 0.039 | 0.012 |
| rs10483727 | -0.094 | 0.039 | 0.016 |
| rs1079866 | -0.105 | 0.039 | 0.007 |
| rs10840031 | -0.098 | 0.039 | 0.013 |
| rs10938397 | -0.093 | 0.039 | 0.016 |
| rs11022756 | -0.089 | 0.038 | 0.020 |
| rs11715566 | -0.096 | 0.039 | 0.015 |
| rs11756454 | -0.097 | 0.039 | 0.013 |
| rs11767400 | -0.098 | 0.039 | 0.012 |
| rs12003641 | -0.097 | 0.039 | 0.014 |
| rs12148769 | -0.103 | 0.039 | 0.008 |
| rs12291726 | -0.089 | 0.038 | 0.020 |
| rs12598642 | -0.093 | 0.039 | 0.017 |
| rs12915845 | -0.099 | 0.039 | 0.011 |
| rs13179411 | -0.103 | 0.039 | 0.009 |
| rs13215865 | -0.101 | 0.039 | 0.010 |
| rs1398217 | -0.100 | 0.039 | 0.011 |
| rs1482853 | -0.096 | 0.039 | 0.014 |
| rs1516883 | -0.104 | 0.040 | 0.010 |
| rs1518080 | -0.097 | 0.039 | 0.014 |
| rs1659127 | -0.099 | 0.039 | 0.012 |
| rs16938437 | -0.096 | 0.039 | 0.014 |
| rs17351680 | -0.093 | 0.038 | 0.015 |
| rs1874984 | -0.103 | 0.039 | 0.009 |
| rs2153127 | -0.093 | 0.040 | 0.020 |
| rs2179786 | -0.098 | 0.039 | 0.013 |
| rs2184968 | -0.111 | 0.037 | 0.003 |
| rs2303100 | -0.099 | 0.039 | 0.012 |
| rs2344508 | -0.102 | 0.039 | 0.009 |
| rs2617056 | -0.099 | 0.039 | 0.012 |
| rs2687729 | -0.106 | 0.039 | 0.006 |
| rs2836950 | -0.104 | 0.039 | 0.008 |
| rs2947411 | -0.098 | 0.039 | 0.012 |
| rs3733632 | -0.098 | 0.039 | 0.013 |
| rs3743266 | -0.096 | 0.039 | 0.014 |
| rs3870341 | -0.104 | 0.039 | 0.008 |
| rs3914188 | -0.101 | 0.039 | 0.011 |
| rs4242496 | -0.096 | 0.039 | 0.014 |
| rs4369815 | -0.096 | 0.039 | 0.014 |
| rs466639 | -0.103 | 0.039 | 0.009 |
| rs4801589 | -0.102 | 0.039 | 0.009 |
| rs4840086 | -0.096 | 0.039 | 0.014 |
| rs618678 | -0.102 | 0.039 | 0.009 |
| rs633715 | -0.092 | 0.039 | 0.017 |
| rs6694738 | -0.102 | 0.039 | 0.009 |
| rs6747380 | -0.112 | 0.038 | 0.003 |
| rs6758290 | -0.100 | 0.039 | 0.011 |
| rs6770162 | -0.102 | 0.039 | 0.009 |
| rs6933660 | -0.102 | 0.039 | 0.009 |
| rs7103411 | -0.093 | 0.039 | 0.016 |
| rs7119712 | -0.097 | 0.039 | 0.013 |
| rs740077 | -0.102 | 0.039 | 0.009 |
| rs7642134 | -0.104 | 0.039 | 0.008 |
| rs7821178 | -0.106 | 0.039 | 0.007 |
| rs7853970 | -0.094 | 0.039 | 0.015 |
| rs7944630 | -0.106 | 0.039 | 0.006 |
| rs852069 | -0.099 | 0.039 | 0.012 |
| rs888345 | -0.104 | 0.039 | 0.008 |
| rs895526 | -0.098 | 0.039 | 0.012 |
| rs913588 | -0.096 | 0.039 | 0.014 |
| rs9373571 | -0.103 | 0.039 | 0.008 |
| rs9555810 | -0.100 | 0.039 | 0.011 |
| rs9565073 | -0.103 | 0.039 | 0.008 |
| rs9635759 | -0.096 | 0.039 | 0.015 |
| rs9647570 | -0.105 | 0.039 | 0.007 |
| rs9939609 | -0.092 | 0.039 | 0.017 |
| rs9997604 | -0.107 | 0.038 | 0.005 |

AAM: Age at menarche; MI: Myocardial infarction; SNPs: Single nucleotide polymorphisms; SE: Standard deviation.

Table S37. MR-PRESSO analysis for estimate of the association of genetically predicted AAM with genetically predicted MI.

| MR. Analysis | Estimate | Sd | T.stat | *P*.value |
| --- | --- | --- | --- | --- |
| Raw | -0.099 | 0.039 | -2.564 | 1.26E-02 |
| Outlier-corrected^*^ | -0.111 | 0.037 | -3.018 | 3.63E-03 |

AAM: Age at menarche; MI: Myocardial infarction. ^*^Number of outliers: 1
